# *Isaria fumosorosea* KCh J2 Entomopathogenic Strain as an Effective Biocatalyst for Steroid Compound Transformations

**DOI:** 10.3390/molecules22091511

**Published:** 2017-09-09

**Authors:** Ewa Kozłowska, Monika Dymarska, Edyta Kostrzewa-Susłow, Tomasz Janeczko

**Affiliations:** Department of Chemistry, Wrocław University of Environmental and Life Sciences, Norwida 25, 50-375 Wrocław, Poland; monika.dymarska@gmail.com (M.D.); ekostrzew@gmail.com (E.K.-S.)

**Keywords:** *Isaria fumosorosea*, biotransformation, dehydroepiandrosterone, DHEA, steroid lactones

## Abstract

The catalytic activity of enzymes produced by an entomopathogenic filamentous fungus (*Isaria fumosorosea* KCh J2) towards selected steroid compounds (androstenedione, adrenosterone, progesterone, 17α-methyltestosterone and dehydroepiandrosterone) was investigated. All tested substrates were efficiently transformed. The structure of the substrate has a crucial impact on regio- and stereoselectivity of hydroxylation since it affects binding to the active site of the enzyme. Androstenedione was hydroxylated in the 7α-position to give a key intermediate in the synthesis of the diuretic-7α-hydroxyandrost-4-ene-3,17-dione with 82% conversion. Adrenosterone and 17α-methyltestosterone were hydroxylated in the 6β-position. Hydroxylated derivatives such as 15β-hydroxy-17α-methyltestosterone and 6β,12β-dihydroxy-17α-methyltestosterone were also observed. In the culture of *Isaria fumosorosea* KCh J2, DHEA was effectively hydroxylated in the C-7 position and then oxidized to give 7-oxo-DHEA, 3β,7α- and 3β,7β-dihydroxy-17a-oxa-d-homo-androst-5-ene-17-one. We obtained 7β-OH-DHEA lactone with 82% yield during 3 days transformation of highly concentrated (5 g/L) DHEA.

## 1. Introduction

Steroid compounds are very common in pharmacy and medicine because of their variety of biological activities [1,2,3]. Hydroxylation of steroid compounds by microbial monooxygenases, which are similar to mammalian cytochromes P450, is a source of molecules of high biological activity and key intermediates in chemical synthesis [4,5,6,7]. The first known hydroxylated corticosteroid is cortisol (hydrocortisone), which is produced by the adrenal glands from cortisone by the 11β-hydroxysteroid dehydrogenase type 1 (11β-HSD1) in response to stress and low blood glucose concentrations. Reduction of the carbonyl group attached to C-11 increases the anti-inflammatory properties relative to cortisone and allows it to be used in dermatology as a topical steroid [8]. These findings caused increased demand for this drug and hence the need to improve the process for obtaining it. Hydroxylation of Reichstein’s S substance in the 11β-position in a culture of *Curvularia lanata* significantly decreased the number of chemical steps (from 36 to 11) and the price of hydrocortisone to less than 1 $ [9] An additional hydroxyl group in the 16α-position eliminates the salt-retaining activity—the most common adverse effect of steroids. These discoveries resulted in obtaining chloro and fluoro derivatives, with much stronger anti-inflammatory activity and fewer side effects, which are used nowadays in medicine [10]. In many cases, biotransformation products have higher biological activity than their precursors. The enzyme selectivity and mild biotransformation conditions contributed to the development of this field of research [11].

Dehydroepiandrosterone (DHEA) is a natural steroid hormone produced by the adrenal cortex. Its hydroxylated derivatives have immunomodulatory, antiproliferative, neuroprotective and anti-inflammatory properties. They are responsible for anti-glucocorticoid action [12,13,14,15,16]. 7α- and 7β-hydroxy derivatives have neuroprotective effects in an animal model of Alzheimer’s disease, improve memory in old mice and affect the mood during the menstrual cycle [15,17,18,19]. 7α-Hydroxy-DHEA extends its antioxidant effect to oxidative damage in the liver earlier than DHEA [13]. 7α-Hydroxyepiandrosterone, another DHEA derivative, is also a very potent anti-inflammatory agent in colitis as well as neuroprotective in cerebral ischemia [20]. Due to high biological activity of 7-hydroxy derivatives of DHEA, 481 strains from different genera were tested for 7-hydroxylase activity by Lobastova et al. [21]. Strains from 64 tested genera were able to perform hydroxylation in this position, the majority giving products with the 7α-configuration. Methods for achieving high yields of 7α-hydroxy-DHEA in cultures of *Absidia coerulea* AM93, *Fusarium culmorum*, *Mortierella isabellina* AM212, *Gelasinospora retispora*, *Mucor silvaticus* as well as 7β-hydroxy-DHEA in the cultures of *Cephalosporium aphidicola*, *Aspergillus wentii* MRC 200316, and *Mortierella isabellina* AM212 are described in the literature [4,22,23,24,25,26,27]. 7α-Hydroxyandrostenedione is also a compound of practical significance used in the pharmaceutical industry in the production of diuretic agents. It can be obtained in cultures of *Chaetomium* sp. KCH 6651, *Didymosphearia igniaria* KCH 6670, *Paecilomyces victoriae* or *Absidia glauca* [7,28,29,30]. 7-Oxo-DHEA affects the levels of thyroid hormones and thermogenesis and can be used to control obesity [31,32,33,34]. Moreover, steroid lactones can inhibit aromatase—an enzyme that catalyzes aromatization of androgens into estrogens, overexpression of which may be observed in hormone-dependent breast cancers [35]. The first steroid aromatase inhibitor used in treatment was testolactone [36]. High activity of Baeyer-Villiger monooxygenase are characteristic to strains from the genera *Penicillium*, e.g., P. *simplicissimum, P. lanosocoeruleum, P. commune* or *P. chrysogenum* [6,37,38].

The biocatalyst used in this work, an *Isaria fumosorosea* strain, belongs to an interesting group of about 700 species (from >100 genera) of entomopathogenic (insect-pathogenic) fungi that constitute a unique, highly specialized trophic subgroup [39,40]. Fungal pathogens of insects are found within every ecosystem and all major fungal lineages with the principal exception of the higher basidiomycetes [40,41,42]. *Isaria fumosorosea* (previously *Paecilomyces fumosoroseus* [43,44]) is a promising mycoinsecticide for the control of the diamondback moth (*Plutella xylostella*), the Asian citrus psyllid (*Diaphorina citri*), whiteflies and other pest insects [45,46,47,48]. Like most fungal pathogens, *I. fumosorosea* directly penetrates the host through the exoskeleton, with the help of a range of hydrolytic enzymes secreted by growing hyphae. These enzymes include chitinases, proteases, and lipases, which destroy the complex and variable structure of the insect cuticle [49,50,51,52,53]. Among the extensive group of entomopathogenic fungi, strains of mainly one species, *Beauveria bassiana*, have been used as a biocatalyst. The species is able to provide highly effective glucosylation of aromatic compounds, as well as polyphenols [54,55,56,57,58,59,60]. It is a well-known biocatalyst for the transformation of steroid compounds. Hydroxylations in the C-7α and C-11α positions are described in the literature [61,62,63,64,65]. Strains from this genus also produce Baeyer-Villiger oxidations to afford D-homo lactones [66,67]. There are various publications describing the transformations of different *B. bassiana* strains to multiple metabolites [61,67].

## 2. Results and Discussion

The purpose of this study was to investigate the catalytic ability of entomopathogenic filamentous fungus of the *Isaria fumosorosea* KCh J2 strain against selected steroid compounds. The strain was isolated from a spider cadaver. Substrates in this study were androstenedione, adrenosterone, progesterone, 17α-methyltestosterone and dehydroepiandrosterone. All substrates were transformed with high conversion in a short period of time. The effect of molecular structure on regio- and stereoselectivity of hydroxylation was observed. An additional goal was to obtain lactone derivatives of DHEA and to optimize the process. The course of biotransformation was established by a screening procedure. The structure of products was determined by analysis of pure fractions obtained from preparative biotransformations.

Product structures were determined by proton nuclear magnetic resonance (^1^H-NMR), carbon-13 nuclear magnetic resonance (^13^C-NMR) (Table 1), heteronuclear multiple-bond correlation spectroscopy (HMBC), heteronuclear multiple-quantum correlation spectroscopy (HMQC), gas chromatography (GC) and thin-layer chromatography (TLC). The spectral characteristics of obtained compounds were in agreement with literature data [4,28,68,69,70].

**Scheme 1:**
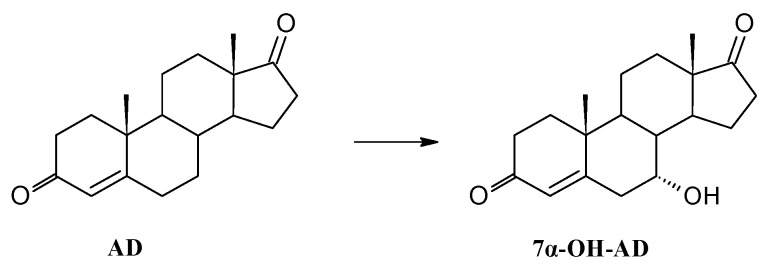
Transformation of androstenedione in *Isaria fumosorosea* KCh J2 culture.

Biotransformation of androstenedione (AD) in *Isaria fumosorosea* KCh J2 culture gave only one monohydroxylation product, 7α-hydroxyandrost-4-ene-3,17-dione (7α-OH-AD), in high yield (Scheme 1). All the added substrate was transformed in less than 24 h (Table 2). Prolongation of the process caused product degradation. There are known microbial methods of obtaining 7α-OH-AD using *Didymosphearia igniaria* KCH 6670, *Chaetomium sp*. KCH 6651, *Neurospora crassa* or *Mucor racemosus*, but their efficiency is low and the number of by-products is substantial [7,28,71,72]. The product can be used in the pharmaceutical industry in the synthesis of diuretics [73].

**Scheme 2:**
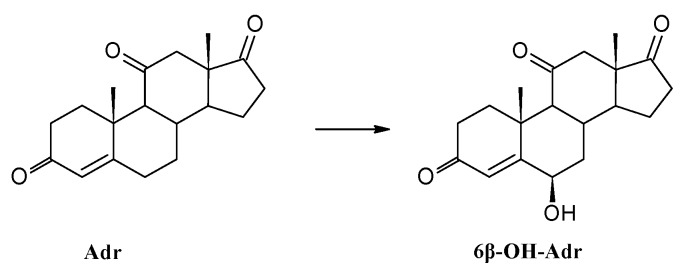
Hydroxylation of adrenosterone by *Isaria fumosorosea* KCh J2 strain.

In contrast, the transformation of adrenosterone (Adr), another substrate with a 3-on-4-ene moiety and a carbonyl group attached to C-17, did not result in the formation of the corresponding 7α-hydroxy derivative (Scheme 2). Probably, the carbonyl group at C-11 is a sterical hindrance and prevents access to the active site of the enzyme and formation of 7α-hydroxyadrenosterone. Instead of this, 6β-hydroxyadrenosterone (6β-OH-Adr) is produced but much more slowly (complete transformation of the substrate in more than 7 days) compared to products of other substrates (Table 2). Reduction of the C-17 carbonyl group of adrenosterone, known from the transformation in cultures of *Cunninghamella elegant*, *Aspergillus tamarii* Kita, *Cephalosporium aphidicola*, *Fusarium lini* or *Trichothecium roseum* [74,75,76], did not happen after 7 days of transformation by *Isaria fumosorosea* KCh J2.

**Scheme 3:**
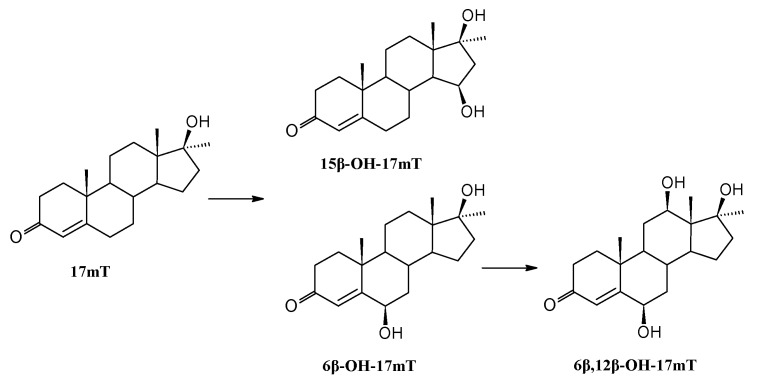
Transformation of 17α-methyltestosterone in *Isaria fumosorosea* KCh J2 culture.

In the culture of *Isaria fumosorosea* KCh J2 17α-methyltestosterone (17mT) was transformed into two monohydroxylated derivatives: 6β-OH-17mT and 15β-OH-17mT, in less than 24 h (Table 2). Additionally, 6β-OH-17mT was hydroxylated in the 12β position, giving the dihydroxylated derivative 6β,12β-OH-17mT (Scheme 3). 6β-Hydroxy- and dihydroxy derivatives were obtained in the culture of *Acremonium strictum* [70].

Ten mg of progesterone was transformed in the culture of *Isaria fumosorosea* KCh J2 strain in less than 24 h, giving many products. Eight fractions from the crude mixture were separated as a result of the transformation of 100 mg of progesterone, but the number of fractions and their purity did not allow the chemical structure of any product to be determined. The number of fractions was astonishing considering the one or three products seen in the transformations of other 3-oxo-4-ene-steroids (AD, Adr, 17mT) used in this study.

**Scheme 4:**
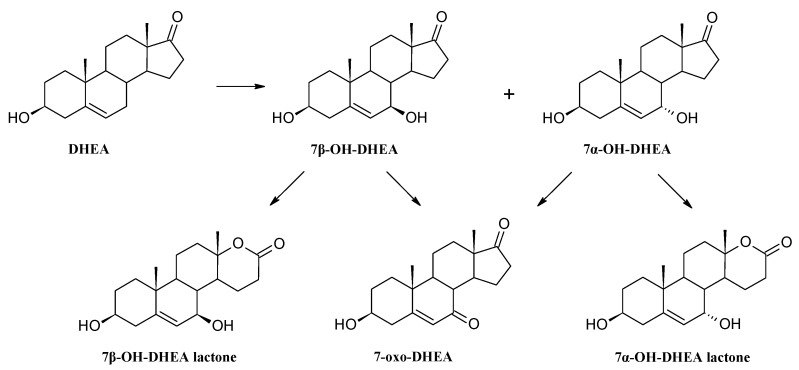
Putative transformation of DHEA in the culture of *Isaria fumosorosea* KCh J2 strain.

Dehydroepiandrosterone (DHEA) was transformed in less than 24 h in the culture of Isaria fumosorosea KCh J2 strain (Table 3). To establish the pathway of DHEA transformation we modified the screening procedure (Section 3.3). This modification confirmed that the substrate was firstly regioselectively transformed to C-7 position monohydroxylated derivatives (7α-OH-DHEA and 7β-OH-DHEA) and then oxidized, to a small extent, to 7-oxo-DHEA. Furthermore, both hydroxy derivatives were transformed to the corresponding D-lactones (Scheme 4). Additional transformations of intermediate products were conducted to verify possible interconversion between isomers. Transformation of 7α-OH-DHEA led to the formation of 7β-OH-DHEA and 7α-OH-DHEA lactone. The addition of 7β-OH-DHEA to the culture of *Isaria fumosorosea* KCh J2 gave an analogous result. Surprisingly, in the transformation of both alcohols, we did not observe any amount of 7-oxo-DHEA. This compound as a substrate was not reduced to any 7-hydroxy derivatives after 3 days of transformation. The total amount of 7-alcohol came from hydroxylation of DHEA, not from the reduction of 7-oxo-DHEA. Milecka-Tronina et al. and Kołek et al. also observed, in cultures of different genera—*Absidia coerulea* AM93 and *Mortierella isabellina* AM212— the oxidation of stereoisomers of 7OH-DHEA to 7-oxo-DHEA but not a stereoselective reduction to the opposite 7-alcohols [4,22]. However, such an interconversion was successfully tested in human, pig and rat liver microsomal fractions containing 11β-hydroxysteroid dehydrogenase (11βHSD) as well as human 11βHSD1 expressed in *Saccharomyces cerevisiae* [77,78,79,80,81]. Isoenzymes of the 11βHSD family catalyze the reaction of activation of cortisone and inactivation of cortisol, but the spectrum of substrates is broader (corticosterone, 7-hydroxy-cholesterol, 7OH-DHEA) and oxidoreductase activity also occurs in the C-7 position [77]. Thus, interconversion of 7OH-DHEA via 7-oxo-DHEA is probably specific only to these animals’ cytochrome P450s. In addition, 7-oxo-DHEA was characterized as an 11β-HSD inhibitor, which can explain the lack of reduction to 7OH-DHEA [77]. In our study interconversion of 7-hydroxy-DHEA stereoisomers was observed but not via the 7-oxo form. We assume that this interconversion is not catalyzed by 11βHSD. Moreover, transformations of intermediates were slower than the transformation of DHEA. In the transformation of DHEA lactones were observed after 1 day of incubation, in contrast to 3 days incubation of the 7β-hydroxy derivative. 7α-OH-DHEA lactone was not transformed to the corresponding lactone in 3 days incubation. Such results indicate that DHEA acts as an inductor for this reaction cascade. 

Due to the rapid transformation of DHEA, an experiment with different DHEA concentrations was performed. Various amounts of the substrate were added to a grown culture of *Isaria fumosorosea* KCh J2 to give a final medium concentration of DHEA in the range of 0.1 to 5.0 g/L. Analysis of the composition of the reaction mixtures indicates that the 7β-OH product is formed in the largest amount (Table 3). Along with the increase of DHEA concentration the ratio of 7α-OH-DHEA to 7β-OH-DHEA decreases. Also, transformation of hydroxy derivatives into the corresponding lactones is faster at a higher concentration of substrate. As we suggested earlier, DHEA seems to be an inductor of the cascade, but this requires further investigation.

## 3. Materials and Methods 

### 3.1. Materials 

The substrates androstenedione, adrenosterone, progesterone, 17α-methyltestosterone and dehydroepiandrosterone (DHEA) were purchased from Sigma-Aldrich (St. Louis, MO, USA). 3β,7β-Dihydroxyandrost-5-ene-17-one (7β-OH-DHEA), 3β,7β-dihydroxyandrost-5-ene-17-one (7β-OH-DHEA) and 3β-hydroxyandrost-5-ene-7,17-dione (7-oxo-DHEA) were isolated as a products of DHEA transformation in the culture of *Isaria fumosorosea* KCh J2 strain. 

The microorganism *Isaria fumosorosea* KCh J2 was obtained from the collection of the Department of Chemistry, Wrocław University of Environmental and Life Sciences (Wrocław, Poland). Isolation and identification procedures were described in our previous paper [82]. The strain was maintained on Sabouraud 4% dextrose-agar slopes and freshly subcultured before use in the transformation experiments.

### 3.2. Screening Procedure

Erlenmeyer flasks (300 mL), each containing 100 mL of the cultivation medium (3% glucose, 1% aminobac), were inoculated with a suspension of *I. fumosorosea* KCh J2 strain and then incubated for 3 days at 24 °C on a rotary shaker. Then 10 mg of a substrate dissolved in 1 mL of tetrahydrofuran (THF) was added. Samples were taken on the 1st, 3rd and 7th day of the process and products were subsequently extracted using chloroform and analyzed using TLC and GC.

### 3.3. Screening Procedure for DHEA 

Incubation of DHEA was carried out in Erlenmeyer flasks (300 mL) containing 100 mL of the cultivation medium. Ten mg of DHEA was added in THF (1 mL) to a 3-day-old culture of the investigated strain. The transformation conditions were the same as in the standard experiment (Section 3.2). Samples were taken at 3, 6, 9, 12 h and on the 1st, 3rd and 7th day of the process, and products were subsequently extracted using chloroform and analyzed using TLC and GC.

### 3.4. Establishing the DHEA Transformation Pathway 

To the 3-day-old culture, cultivated as described in Section 3.2, Ten mg of 7α-OH-DHEA, 7β-OH-DHEA, 7-oxo-DHEA and DHEA (control) dissolved in 1 mL of THF was added to each flask. Samples were taken at 6 and 12 h and on the 1st and 3rd day of the process and products were subsequently extracted using chloroform and analyzed using TLC.

### 3.5. Transformation Procedure for Different DHEA Concentrations

To each flask containing 100 mL of the 3-day-old culture of *Isaria fumosorosea* KCh J2, cultivated as described in Section 3.2, DHEA (50, 100, 200 or 500 mg) dissolved in THF (2 mL) was added. The final concentration of DHEA in the culture was 0.5, 1.0, 2.0 and 5.0 g/L, respectively. Samples were taken at 6 and 12 h and on the 1st and 3rd day of the process and products were subsequently extracted using chloroform and analyzed using TLC and GC.

### 3.6. Preparative Biotransformation

The same transformations were performed on the preparative scale in 2000 mL flasks, each containing 500 mL of the cultivation medium. The culture of *I. fumosorosea* KCh J2 was incubated under the same conditions as in the screening procedure and then 100 mg of substrate dissolved in 2 mL of THF was added to the 3-day-old culture. After the complete transformation of the substrate, the mixture was extracted with CHCl_3_ (3 × 300 mL), dried (MgSO_4_) and concentrated in vacuo. The crude mixture obtained this way was separated by preparative TLC and analyzed (TLC, GC).

### 3.7. Analytical Methods

The course of the biotransformation was monitored by means of TLC. The composition of product mixtures was established by GC. The crude mixture was separated by preparative TLC (Silica Gel GF, 500 μm, Analtech, Newark, DE, USA) and hexane/acetone mixture (2:1, *v*/*v*) as an eluent. After elution products were detected under UV light (365 nm) then scraped from the plate and eluted with acetone to give fractions. Analytical TLC was carried out on silica gel G (Merck, Darmstadt, Germany). Compounds were detected by spraying the plates with a H_2_SO_4_/CH_3_OH mixture (1:1, *v*/*v*) and visualized under UV light (254 nm). GC analysis was performed using a Hewlett-Packard 5890A (Series II) GC instrument fitted with a flame ionization detector (FID) (nowadays Agilent, Santa Clara, CA, USA). The DB-5MS (cross-linked phenyl- methylsiloxane) capillary column (30 m × 0.32 mm × 0.25 μm) was used to determine the composition of product mixtures. The following temperature program was used: 220 °C (1 min)/4 °C/min/260 °C (1 min)/30 °C/min/300 °C (5 min). For gas chromatography – mass spectrometry GC-MS analysis, a GCMS-SATURN 2000 instrument (Varian, nowadays Agilent, Santa Clara, CA, USA) was used with a ZB-1 (crosslinked phenyl-methylsiloxane) capillary column (30 m × 0.25 mm × 0.25 μm). The following temperature programme was used: 220 °C (1 min)/5 °C/1 min/300 °C (7 min) (Supplementary Materials). The NMR spectra were recorded on a DRX 600 MHz spectrometer (Bruker, Bruker, Billerica, MA, USA) and measured in CDCl_3_. Products poorly soluble in chloroform were dissolved in DMSO-*d*_6_ or THF-*d*_8_. The products’ structures were determined by means of elemental analysis, ^1^H-NMR, ^13^C-NMR and correlation spectroscopy (HMBC, HMQC).

### 3.8. Spectral Data of Isolated Metabolites

#### 3.8.1. Transformation of Androstenedione (AD)

After 24 h transformation of 100 mg of androstenedione in *Isaria fumosorosea* KCh J2 culture 42.3 mg (42%) of 7α-hydroxyandrost-4-ene-3,17-dione (7α-OH-AD) was isolated as the sole product (Supplementary Materials). 

*7α-Hydroxyandrost-4-ene-3,17-dione* (**7α-OH-AD**). ^1^H-NMR (600 MHz) (ppm) (CDCl_3_) δ: 0.89 (s, 3H, 18-H); 1.20 (s, 3H, 19-H); 1.26 (td, 1H, *J* = 13.1, 3.6 Hz, 12-Hα); 1.45 (td, 1H, *J* = 13.0, 3.5 Hz, 11-Hβ); 1.50–1.60 (m, 2H, 9-H, 15-Hβ); 1.68–1.79 (m, 4H, 1-Hα, 8-H, 11-Hα, 14-H); 1.82 (dm, 1H, *J* = 13.0 Hz, 12-Hβ); 2.01–2.13 (m, 3H, 1-Hb, 15-Hα, 16-Hα); 2.35 (dm, 1H, *J* = 16.6 Hz, 2-Hα); 2.37–2.49 (m, 3H, 2-Hβ, 6-Hα, 16-Hβ); 2.65 (d, 1H, *J* = 14.9 Hz, 6-Hβ); 4.09 (s, 1H, 7-Hβ); 5.78 (s, 1H, 4-H).

#### 3.8.2. Transformation of Adrenosterone (Adr)

After 24 h incubation of 100 mg adrenosterone in *Isaria fumosorosea* KCh J2 culture 33.1 mg (33%) of 6β-hydroxyandrost-4-ene-3,11,17-trione (6β-OH-Adr) was isolated as the sole product (Supplementary Materials). 

*6β-Hydroxyandrost-4-ene-3,11,17-trione* (**6β-OH-Adr**). ^1^H-NMR (600 MHz) (ppm) (CDCl_3_) δ: 0.89 (s, 3H, 18-H); 1.50 (ddd, 1H, *J* = 14.1, 11.6, 2.6 Hz, 7-Hα); 1.60 (s, 3H, 19-H); 1.62 (td, 1H, *J* = 14.5, 4.0 Hz, 1-Hα); 1.73 (ddd, 1H, *J* = 21.4, 12.1, 9.4 Hz, 15-Hβ); 1.86 (d, 1H, *J* = 11.5 Hz, 9-H); 1.89 (td, 1H, *J* = 11.8, 5.7 Hz, 14-H); 2.12–2.18 (m, 1H, 15-Hα); 2.20–2.37 (m, 4H, 2-Hα, 7-Hβ, 12-Hα, 16-Hα); 2.44–2.60 (m, 4H, 2-Hβ, 8-H, 12-Hβ, 16-Hβ); 2.80 (dm, 1H, *J* = 13.4 Hz, 1-Hβ); 4.39 (br s, 1H, 6-Hα); 5.79 (br s, 1H, 4-H).

#### 3.8.3. Transformation of 17α-Methyltestosterone (17mT)

After 7 days transformation of 100 mg of 17α-methyltestosterone in *Isaria fumosorosea* KCh J2 culture 8.4 mg (8%) of 6β-hydroxy-17α-methyltestosterone (6β-OH-17mT), 5.1 mg (5%) of 15β-hydroxy-17α-methyltestosterone (15β-OH-17mT) and 34.3 mg (34%) of 6β,12β-dihydroxy-17α-methyltestosterone (6β,12β-OH-17mT) were isolated (Supplementary Materials). 

*6β-Hydroxy-17α-methyltestosterone* (**6β-OH-17mT**). ^1^H-NMR (600 MHz) (ppm) (CDCl_3_) δ: 0.89 (td, 1H *J* = 11.3, 4.3 Hz, 9-H); 0.94 (s, 3H, 18-H); 1.19–1.23 (m, 2H, 7-Hα, 14-H); 1.22 (s, 3H, 20-H); 1.28–1.38 (m, 2H, 12-Hα, 15-Hβ); 1.40 (s, 3H, 19-H); 1.50 (qd, 1H, *J* = 12.7, 4.0 Hz, 11-Hα); 1.55–1.64 (m, 3H, 11-Hβ, 12-Hβ, 15-Hα); 1.69 (ddd, 1H, *J* = 14.5, 13.4, 4.2 Hz, 1-Hα); 1.76 (ddd, 1H, *J* = 14.2, 9.6, 6.4 Hz, 16-Hα); 1.84 (qd, 1H, *J* = 14.1, 3.5 Hz, 16-Hβ); 2.01 (dt, 1H, *J* = 13.5, 3.0 Hz, 7-Hβ); 2.03–2.07 (m, 2H, 1-Hβ, 8-H); 2.39 (dddd, 1H, *J* = 16.9, 4.0, 2.8, 0.7 Hz, 2-Hα); 2.52 (ddd, 1H, *J* = 16.8, 15.1, 5.0 Hz, 2-Hβ); 4.35 (t, 1H, *J* = 2.8 Hz, 6-Hα); 5.82 (s, 1H, 4-H). 

Due to the low solubility of the resulting product in CDCl_3_, the compound was dissolved in the deuterated THF(THF-*d*_8_) and NMR analysis was performed again (Supplementary Materials). 

*6β-Hydroxy-17α-methyltestosterone* (**6β-OH-17mT**). ^1^H-NMR (600 MHz) (ppm) (THF-*d_8_*) δ: 0.87 (td, 1H *J* = 11.3, 4.3 Hz, 9-H); 0.90 (s, 3H, 18-H); 1.13 (s, 3H, 20-H); 1.13 (tdd, 1H, *J* = 12.7, 3.1, 1.3 Hz, 7-Hα); 1.21 (d, 1H, *J* = 11.9, 10.8, 4.3 Hz, 14-H); 1.25–1.33 (m, 2H, 12-Hα, 15-Hβ); 1.38 (s, 3H, 19-H); 1.48–1.55 (m, 2H, 11-Hα, 12-Hβ); 1.56–1.62 (m, 3H, 11-Hβ, 15-Hα, 16-Hα); 1.66 (ddd, 1H, *J* = 14.6, 13.4, 4.3 Hz, 1-Hα); 1.80–1.86 (m, 1H, 16-Hβ); 1.93 (dt, 1H, *J* = 13.5, 3.0 Hz, 7-Hβ); 2.01 (ddd, 1H, *J* = 13.1, 4.9, 2.8 Hz, 1-Hβ); 2.08 (qd, 1H, *J* = 11.0, 3.2 Hz, 8-H); 2.22 (dddd, 1H, *J* = 16.9, 3.9, 2.8, 0.8 Hz, 2-Hα); 2.44 (ddd, 1H, *J* = 16.8, 15.2, 5.0 Hz, 2-Hβ); 3.29 (s, 1H, 17-O*H*); 4.17 (q, 1H, *J* = 2.8 Hz, 6-Hα); 4.29 (dd, 1H, *J* = 2.7, 1.4 Hz, 6-O*H*); 5.66 (s, 1H, 4-H).

*15β-Hydroxy-17α-methyltestosterone* (**15β-OH-17mT**). ^1^H-NMR (600 MHz) (ppm) (CDCl_3_) δ: 0.99 (td, 1H, *J* = 12.2, 3.9 Hz, 9-H); 1.06–1.13 (m, 2H, 7-Hα, 14-H); 1.15 (s, 3H, 18-H); 1.18 (s, 3H, 19-H); 1.24 (s, 3H, 20-H); 1.29 (td, 1H, *J* = 13.7, 4.7 Hz, 12-Hα); 1.46 (qd, 1H, *J* = 12.9, 3.3 Hz, 11-Hβ); 1.52 (td, 1H, *J* = 12.4, 3.6 Hz, 12-Hβ); 1.63 (qd, 1H, *J* = 11.6, 4.2 Hz, 11-Hα); 1.74 (td, 1H, *J* = 13.9, 4.8 Hz, 1-Hα); 2.00 (qd, 1H, *J* = 11.0, 3.2 Hz, 8-H); 2.05 (ddd, 1H, *J* = 13.7, 5.2, 3.3 Hz, 1-Hβ); 2.10 (ddt, 1H, *J* = 12.5, 5.5, 2.9 Hz, 7-Hβ); 2.27 (dd, 1H, *J* = 14.4, 3.9, 2.7 Hz, 6-Hα); 2.30-2.37 (m, 3H, 2-Hα, 16-Hα, 16-Hβ); 2.43 (ddd, 1H, *J* = 19.5, 11.8, 5.2 Hz, 2-Hβ); 2.48 (td, 1H, *J* = 14.4, 1.7 Hz; 6-Hβ); 4.20 (ddd, 1H, *J* = 7.9, 5.8, 2.4 Hz, 15-Hα); 5.74 (s, 1H, 4-H). 

Due to the low solubility of the resulting product in CDCl_3_, the compound was dissolved in the deuterated DMSO (DMSO-*d*_6_) and NMR analysis was performed again (Supplementary Materials).

*6β,12β-Dihydroxy-17α-methyltestosterone* (**6β,12β-OH-17mT**). ^1^H-NMR (600 MHz) (ppm) (DMSO-*d*_6_) δ: 0.81 (s, 3H, 18-H); 0.90 (ddd, 1H, *J* = 11.7, 10.1, 4.2 Hz, 9-H); 0.98–1.09 (m, 2H, 7-Hα, 14-H); 1.21 (s, 3H, 20-H); 1.22–1.27 (m, 1H, 15-Hβ); 1.28 (s, 3H, 19-H); 1.32 (q, 1H, *J* = 12.0 Hz, 11-Hα); 1.48–1.55 (m, 3H, 11-Hβ, 15-Hα, 16-Hα); 1.60 (ddd, 1H, *J* = 14.6, 13.4, 4.1 Hz, 1-Hα); 1.73 (t, 1H, *J* = 11.2 Hz, 16-Hβ); 1.78 (dt, 1H, *J* = 13.6, 3.0 Hz, 7-Hβ); 1.82 (qd, 1H, *J* = 11.2, 2.8 Hz, 8-H); 1.90 (ddd, 1H, *J* = 13.0, 4.7, 2.6 Hz, 1-Hβ); 2.20 (ddd, 1H, *J* = 16.9, 3.9, 2.8 Hz, 2-Hα); 2.45 (ddd, 1H, *J* = 16.9, 15.4, 4.7 Hz, 2-Hβ); 3.52–3.56 (m, 1H, 12-Hα); 3.57 (s, 1H, 17-O*H*); 4.00 (d, 1H, *J* = 3.6 Hz, 12-O*H*); 5.11 (q, 1H, *J* = 2.5 Hz, 6-Hα); 5.11 (d, 1H, *J* = 2.5 Hz, 6-O*H*); 5.66 (s, 1H, 4-H).

#### 3.8.4. Transformation of Progesterone (P)

The complete transformation of 100 mg of progesterone by *Isaria fumosorosea* KCh J2 culture in 48 h gave many products. Eight fractions from crude mixture were separated by preparative TLC. NMR analysis of each fraction exposed a mixture of products. Analysis of NMR, TLC and GC data ensured that the biotransformation of progesterone was effective although the quantity of products makes identification impossible.

#### 3.8.5. Transformation of dehydroepiandrosterone (DHEA)

After 12 h transformation of 200 mg of dehydroepiandrosterone in *Isaria fumosorosea* KCh J2 culture 10.2 mg (5%) of 3β,7α-dihydroxyandrost-5-ene-17-one (7α-OH-DHEA), 86.4 mg (43%) of 3β,7β-dihydroxyandrost-5-ene-17-one (7β-OH-DHEA), 5.8 mg (3%) of 3β-hydroxyandrost-5-ene-7,17-dione (7-oxo-DHEA) and 31.7 mg (16%) of 3β,7β-dihydroxy-17a-oxa-d-homo-androst-5-ene-17-one (7β-OH-DHEA lactone) were isolated. After 1 day transformation of 200 mg of dehydroepiandrosterone in *Isaria fumosorosea* KCh J2 culture 30.1 mg (15%) 3β,7α-dihydroxy-17a-oxa-d-homo-androst-5-ene-17-one (7α-OH-DHEA lactone) and 119.8 mg (60%) of 3β,7β-dihydroxy-17a-oxa-d-homo-androst-5-ene-17-one (7β-OH-DHEA lactone) were isolated (Supplementary Materials). 

*3β,7α-Dihydroxyandrost-5-ene-17-one* (**7α-OH-DHEA**). ^1^H-NMR (600 MHz) (ppm) (CDCl_3_) δ: 0.87 (s, 3H, 18-H); 1.01 (s, 3H, 19-H); 1.11 (td, 1H, *J* = 13.4, 3.8 Hz, 1-Hα); 1.23–1.31 (m, 2H, 9-H, 12-Hα); 1.49 (td, 1H, *J* = 13.1, 4.3 Hz, 11-Hα); 1.50–1.60 (m, 2H, 2-Hα, 15-Hα); 1.64–1.72 (m, 2H, 8-H, 11-Hβ); 1.78 (td, 1H, *J* = 12.1, 5.3 Hz, 14-H); 1.80–1.89 (m, 3H, 1-Hβ, 2-Hβ, 12-Hβ); 2.07–2.17 (m, 2H, 15-Hβ, 16-Hα); 2.29 (br t, 1H, *J* = 12.3 Hz, 4-Hα); 2.35 (ddd, 1H, *J* = 13.3, 4.8, 2.0 Hz, 4-Hβ); 2.33 (dd, 1H, *J* = 13.1, 4.6 Hz, 16-Hβ); 3.56 (tt, 1H, *J* = 11.3, 4.7 Hz, 3-Hα); 3.96 (br t, 1H, *J* = 3.8 Hz, 7-Hβ); 5.63 (dd, 1H, *J* = 5.1, 1.2 Hz, 6-H).

*3β,7β-Dihydroxyandrost-5-ene-17-one* (**7β-OH-DHEA**). ^1^H-NMR (600 MHz) (ppm) (CDCl_3_) δ: 0.88 (s, 3H, 18-H); 1.05 (td, 1H, *J* = 13.4, 3.7 Hz, 1-Hα); 1.06 (s, 3H, 19-H); 1.10 (td, 1H, *J* = 12.1, 4.6 Hz, 9-H); 1.25 (td, 1H, *J* = 13.7, 4.7 Hz, 12-Hα); 1.44 (ddd, 1H, *J* = 12.5, 11.0, 5.9 Hz, 14-H); 1.47–1.55 (m, 2H, 2-Hα, 11-Hα); 1.57 (td, 1H, *J* = 11.2, 8.2 Hz, 8-H); 1.70 (dtd, 1H, *J* = 13.8, 4.3, 2.9 Hz, 11-Hβ); 1.81–1.89 (m, 4H, 1-Hβ, 2-Hβ, 12-Hβ, 15-Hβ); 2.11 (dt, 1H, *J* = 19.0, 9.1 Hz, 16-Hα); 2.24 (ddd, 1H, *J* = 12.3, 8.7, 6.0 Hz, 15-Hα); 2.26 (ddt, 1H, *J* = 13.4, 11.3, 2.1 Hz, 4-Ha); 2.35 (ddd, 1H, *J* = 13.2, 4.9, 2.1 Hz, 4-Hβ); 2.45 (dd, 1H, *J* = 19.4, 8.6 Hz, 16-Hβ); 3.55 (tt, 1H, *J* = 11.3, 4.4 Hz, 3-Hα); 3.95 (dt, 1H, *J* = 8.1, 2.2 Hz, 7-Hα); 5.31 (t, 1H, *J* = 1.9 Hz, 6-H).

*3β-Hydroxyandrost-5-ene-7,17-dione* (**7-oxo-DHEA**). ^1^H-NMR (600 MHz) (ppm) (CDCl_3_) δ: 0.89 (s, 3H, 18-H); 1.22 (s, 3H, 19-H); 1.23–1.34 (m, 2H, 1-Hα, 12-Hα); 1.54–1.68 (m, 4H, 2-Hα, 9-H, 11-Hα, 14-H); 1.70–1.91 (m, 3H, 11-Hβ, 12-Hβ, 15-Hα); 1.91–2.00 (m, 2H, 1-Hβ, 2-Hβ); 2.14 (dd, 1H, *J* = 18.8, 9.8 Hz, 16-Hα); 2.35–2.45 (m, 2H, 4-Ha, 8-H); 2.46 (dd, 1H, *J* = 18.8, 8.3 Hz, 16-Hβ); 2.54 (ddd, 1H, *J* = 13.9, 4.6, 2,2 Hz, 4-Hβ); 2.81 (ddd, 1H, *J* = 13.4, 8.7, 7.2 Hz, 15-Hβ); 3.68 (tt, 1H, *J* = 11.3, 4.8 Hz, 3-Hα); 5.74 (br s, 1H, 6-H)

*3β,7α-Dihydroxy-17a-oxa-d-homo-androst-5-ene-17-one* (**7α-OH-DHEA lactone**). ^1^H-NMR (600 MHz) (ppm) (CDCl_3_) δ: 0.97 (s, 3H, 19-H); 1.13 (td, 1H, *J* = 13.6, 3.7 Hz, 1-Hα); 1.33 (s, 3H, 18-H); 1.34–1.40 (m, 3H, 9-H, 11-Hα, 14-H); 1.50–1.56 (m, 2H, 2-Hα, 15-Hα); 1.67 (td, 1H, *J* = 12.8, 4.0 Hz, 12-Hα); 1.81 (m, 1H, 11-Hβ); 1.86-1.91 (m, 2H, 1-Hβ, 2-Hβ); 1.94–2.02 (m, 2H, 8-H, 12-Hβ); 2.11 (dddd, 1H, *J* = 13.1, 8.5, 4.6, 3.0 Hz, 15-Hβ); 2.24 (ddt, 1H, *J* = 13.2, 11.5, 1.7 Hz, 4-Ha); 2.37 (dd, 1H, *J* = 13.4, 5.1, 2.1 Hz, 4-Hβ); 2.63 (td, 1H, *J* = 18.8, 8.9 Hz, 16-Hα); 2.69 (ddd, 1H, *J* = 18.8, 8.7, 2.5 Hz, 16-Hβ); 3.59 (tt, 1H, *J* = 11.4, 4.6 Hz, 3-Hα); 3.99 (br s, 1H, 7-Hβ); 5.62 (dd, 1H, *J* = 5.2, 1.7 Hz, 6-H).

*3β,7β-Dihydroxy-17a-oxa-d-homo-androst-5-ene-17-one* (**7β-OH-DHEA lactone**). ^1^H-NMR (600 MHz) (ppm) (CDCl_3_) δ: 1.03 (s, 3H, 19-H); 1.10 (td, 1H, *J* = 13.2, 3.5 Hz, 1-Hα); 1.25 (td, 1H, *J* = 12.2, 3.9 Hz, 9-H); 1.32 (td, 1H *J* = 7.3, 3.5 Hz, 8-H); 1.34 (s, 3H, 18-H); 1.51 (ddd, 1H, *J* = 13.6, 9.6, 2.9 Hz, 2-Hα); 1.59 (ddd, 1H, *J* = 12.2, 10.5, 4.3 Hz, 14-H); 1.65 (td, 1H, *J* = 13.2, 4.0 Hz, 12-Hα); 1.74–1.83 (m, 2H, 11-Hα, 11-Hβ, 15-Hα); 1.85–1.91 (m, 2H, 1-Hβ, 2-Hβ); 1.98 (dt, 1H, *J* = 12.6, 3.4 Hz, 12-Hβ); 2.24 (ddt, 1H, *J* = 13.4, 11.5, 2.0 Hz, 4-Ha); 2.37 (ddd, 1H, *J* = 13.4, 4.8, 2.4 Hz, 4-Hβ); 2.44 (dddd, 1H, *J* = 9.4, 7.1, 4.7, 1.9 Hz, 15-Hβ); 2.57 (dt, 1H, *J* = 18.8, 9.3 Hz, 16-Hα); 2.69 (ddd, 1H, *J* = 18.9, 8.4, 1.9 Hz, 16-Hβ); 3.57 (tt, 1H, *J* = 11.3, 4.5 Hz, 3-Hα); 3.95 (br d, 1H, *J* = 5.2 Hz, 7-Hα); 5.27 (t, 1H, *J* = 2.3 Hz, 6-H).

## 4. Conclusions

The entomopathogenic fungus *Isaria fumosorosea* KCh J2 has broad ability to transform steroid substrates into the corresponding hydroxylated derivatives. Transformations of androstenedione gave 7α-OH-AD as the sole product in high yield after a short period of time. Transformation of DHEA gave hydroxylated D ring lactones. The strain is able to transform the substrate in a concentration of 5.0 g/L in less than 72 h. With such a large amount of substrate, we observed higher specificity of hydroxylation and faster transformation to the corresponding lactones. The high substrate specificity and acceptance of a high substrate concentration by this strain could be utilized in the production of steroid compounds on an industrial scale. These results encourage further investigation of metabolic activity of *Isaria fumosorosea* KCh J2 and other entomopathogenic fungi. 

## Figures and Tables

**Table 1:** ^13^C-NMR chemical shifts of products in CDCl_3_.

Atom Number	Products										
	7α-OH-AD	6β-OH-Adr	6β-OH-17mT	6β-OH-17mT ^a^	15β-OH-17mT	6β,12β-OH-17mT ^b^	7α-OH-DHEA	7β-OH-DHEA	7α-OH-DHEA-Lactone	7β-OH-DHEA-Lactone	7-oxo-DHEA
1	35.40	36.10	38.36	38.50	34.11	36.58	37.07	37.02	36.84	36.72	36.60
2	33.89	34.11	34.39	35.08	32.87	33.89	31.39	31.60	31.32	31.53	31.25
3	198.83	200.67	200.53	198.91	199.71	199.22	71.25	71.30	71.20	71.11	70.44
4	127.03	127.17	126.55	126.46	124.07	125.21	42.06	41.77	42.02	41.47	41.99
5	167.25	166.61	168.38	169.45	171.20	168.84	146.64	143.78	146.29	144.00	166.27
6	41.01	72.13	73.25	73.40	32.78	71.07	123.67	125.65	123.43	125.15	126.06
7	67.01	38.10	39.05	40.11	32.22	37.52	64.37	72.91	63.44	72.21	201.20
8	39.37	30.65	30.70	31.82	35.94	29.34	37.32	40.54	40.24	43.42	44.47
9	45.31	63.13	53.77	55.32	54.32	51.68	42.72	48.37	41.76	48.13	50.22
10	38.55	37.68	38.22	39.14	38.95	38.27	37.63	36.78	37.54	36.52	38.54
11	20.15	207.41	20.78	21.85	20.70	29.54	20.19	20.51	21.70	22.07	20.72
12	30.98	50.50	31.54	32.79	31.47	71.83	31.18	31.36	38.53	39.15	30.85
13	47.31	50.44	45.60	46.58	44.89	48.41	47.23	47.89	83.50	83.46	48.00
14	45.64	49.71	50.29	51.57	54.77	49.35	45.05	51.34	40.15	47.20	45.88
15	21.25	21.69	23.30	24.37	69.16	22.67	22.02	24.31	19.86	21.55	24.31
16	35.71	36.03	37.29	39.89	51.97	38.02	35.91	36.10	28.86	29.26	35.77
17	220.41	217.24	81.73	80.90	81.40	80.42	221.30	221.31	171.83	171.79	220.52
18	13.49	14.91	14.11	14.71	16.67	8.75	13.39	13.70	20.08	20.38	13.89
19	17.01	19.32	19.68	19.94	17.47	18.86	18.38	19.29	18.33	19.07	17.57
20			25.91	26.64	25.41	25.83					

^a^ Compound 6β-OH-mT in THF-*d*_8_; ^b^ Compound 6β,12β-mT in DMSO-*d*_6_.

**Table 2:** Product accumulation during transformation in *Isaria fumosorosea* KCh J2 culture.

Substrate	Compounds Found in the Reaction Mixture (%)	Biotransformation Time (Days)		
		1	3	7
Androstenedione	AD	-	-	-
	7α-OH-AD	76	71	64
Adrenosterone	Adr	38	28	11
	6β-OH-Adr	57	67	84
17α-Methyltestosterone	17mT	-	-	-
	6β-OH-17mT	76	49	20
	15β-OH-17mT	11	9	8
	6β,12β-OH-17mT	4	67	84

**Table 3:** Compositions of crude mixtures obtained in transformations of different amounts of DHEA.

Concentration of Substrate (g/L)	Compounds Found in the Reaction Mixture (%)	Biotransformation Time (h)				
		3	12	24	72	168
0.1	DHEA	7	-	-	-	-
	7α-OH-DHEA	15	2	-	-	-
	7β-OH-DHEA	75	34	18	8	-
	7-oxo-DHEA	2	9	6	-	-
	7α-OH-DHEA lactone	-	13	20	22	22
	7β-OH-DHEA lactone	-	41	52	60	58
0.5	DHEA	74	2	-	-	-
	7α-OH-DHEA	5	1	-	-	-
	7β-OH-DHEA	21	16	-	-	-
	7-oxo-DHEA	-	1	-	-	-
	7α-OH-DHEA lactone	-	15	18	17	8
	7β-OH-DHEA lactone	-	62	76	72	74
1.0	DHEA	75	1	-	-	-
	7α-OH-DHEA	5	2	-	-	-
	7β-OH-DHEA	20	36	-	-	-
	7-oxo-DHEA	-	2	-	-	-
	7α-OH-DHEA lactone	-	12	19	20	15
	7β-OH-DHEA lactone	-	45	76	70	66
2.0	DHEA	89	8	-	-	-
	7α-OH-DHEA	2	7	-	-	-
	7β-OH-DHEA	9	58	2	-	-
	7-oxo-DHEA	-	4	-	-	-
	7α-OH-DHEA lactone	-	1	16	14	12
	7β-OH-DHEA lactone	-	20	78	78	79
5.0	DHEA	96	56	26	-	-
	7α-OH-DHEA	1	5	6	-	-
	7β-OH-DHEA	3	33	37	-	-
	7-oxo-DHEA	-	1	3	-	-
	7α-OH-DHEA lactone	-	-	2	12	11
	7β-OH-DHEA lactone	-	4	23	81	75

## Supplementary Materials

Supplementary materials are available online.
