# Supplementary material for: Isaria fumosorosea KCh J2 Entomopathogenic Strain as an Effective Biocatalyst for Steroid Compound Transformations
Source: Molecules. 2017 Sep 9;22(9):1511. doi: 10.3390/molecules22091511 (PMC6151793; doi:10.3390/molecules22091511)

Supplementary Data

Isaria fumosorosea KCh J2 - entomopathogenic strain as an effective biocatalyst of steroid compounds

Ewa Kozłowska,* Monika Dymarska, Edyta Kostrzewa-Susłow, and Tomasz Janeczko*

Department of Chemistry, Wrocław University of Environmental and Life Sciences, Norwida 25, 50-375 Wrocław, Poland.

*Corresponding author: E-mail: e.a.kozlowska@gmail.com (Ewa Kozłowska); janeczko13@interia.pl (Tomasz Janeczko)

Content

Fig.S1. ^{1}H NMR spectral of 7α-hydroxyandrost-4-ene-3,17-dione (**7α-OH-AD**) ($CDCl_3$, 600 MHz)

Fig.S2. ^{13}C NMR spectral of 7α-hydroxyandrost-4-ene-3,17-dione (**7α-OH-AD**) ($CDCl_3$, 151 MHz)

Fig.S3. HSQC spectral of 7α-hydroxyandrost-4-ene-3,17-dione (**7α-OH-AD**) ($CDCl_3$, 151 MHz)

Fig.S4. COSY spectral of 7α-hydroxyandrost-4-ene-3,17-dione (**7α-OH-AD**) ($CDCl_3$, 151 MHz)

Fig.S5. ^{1}H NMR spectral of 6β-hydroxyandrost-4-ene-3,11,17-trione (**6β-OH-Adr**) ($CDCl_3$, 600 MHz)

Fig.S6. ^{13}C NMR spectral of 6β-hydroxyandrost-4-ene-3,11,17-trione (**6β-OH-Adr**) ($CDCl_3$, 151 MHz)

Fig.S7. HSQC spectral of 6β-hydroxyandrost-4-ene-3,11,17-trione (**6β-OH-Adr**) ($CDCl_3$, 151 MHz)

Fig.S8. COSY spectral of 6β-hydroxyandrost-4-ene-3,11,17-trione (**6β-OH-Adr**) ($CDCl_3$, 151 MHz)

Fig.S9. ^{1}H NMR spectral of 15β-hydroxy-17α-methyltestosterone (**15β-OH-mT**) ($CDCl_3$, 600 MHz)

Fig.S10. ^{13}C NMR spectral of 15β-hydroxy-17α-methyltestosterone **(15β-OH-mT)** ($CDCl_3$, 151 MHz)

Fig.S11. ^{1}H NMR spectral of 6β-hydroxy-17α-methyltestosterone (**6β-OH-17mT**) ($CDCl_3$, 600 MHz)

Fig.S12. ^{13}C NMR spectral of 6β-hydroxy-17α-methyltestosterone (**6β-OH-17mT**) ($CDCl_3$, 151 MHz)

Fig.S13. ^{1}H NMR spectral of 6β-hydroxy-17α-methyltestosterone (**6β-OH-17mT**) (THF, 600 MHz)

Fig.S14. ^{13}C NMR spectral of 6β-hydroxy-17α-methyltestosterone (**6β-OH-17mT**) (THF, 151 MHz)

Fig.S15. HSQC spectral of 6β-hydroxy-17α-methyltestosterone (**6β-OH-17mT**) (THF, 151 MHz)

Fig.S16. COSY spectral of 6β-hydroxy-17α-methyltestosterone (**6β-OH-17mT**) (THF, 151 MHz)

Fig.S17. ^{1}H NMR spectral of 6β,12β-dihydroxy-17α-methyltestosterone (**6β,12β-OH-17mT**) ($CDCl_3$, 600 MHz)

Fig.S18. ^{1}H NMR spectral of 6β,12β-dihydroxy-17α-methyltestosterone (**6β,12β-OH-17mT**) (DMSO, 600 MHz)

Fig.S19. ^{13}C NMR spectral of 6β,12β-dihydroxy-17α-methyltestosterone (**6β,12β-OH-17mT**) (DMSO, 151 MHz)

Fig.S20. HSQC spectral of 6β,12β-dihydroxy-17α-methyltestosterone (**6β,12β-OH-17mT**) (DMSO, 151 MHz)

Fig.S21. COSY spectral of 6β,12β-dihydroxy-17α-methyltestosterone (**6β,12β-OH-17mT**) (DMSO, 151 MHz)

Fig.S22. ^{1}H NMR spectral of 3β,7α-dihydroxyandrost-5-ene-17-one (**7α-OH-DHEA**) ($CDCl_3$, 600 MHz)

Fig.S23. ^{13}C NMR spectral of 3β,7α-dihydroxyandrost-5-ene-17-one (**7α-OH-DHEA**) ($CDCl_3$, 151 MHz)

Fig.S24. HSQC spectral of 3β,7α-dihydroxyandrost-5-ene-17-one (**7α-OH-DHEA**) ($CDCl_3$, 151 MHz)

Fig.S25. COSY spectral of 3β,7α-dihydroxyandrost-5-ene-17-one (**7α-OH-DHEA**) ($CDCl_3$, 151 MHz)

Fig.S26. ^{1}H NMR spectral of 3β,7β-dihydroxyandrost-5-ene-17-one (**7β-OH-DHEA**) ($CDCl_3$, 600 MHz)

Fig.S27. ^{13}C NMR spectral of 3β,7β-dihydroxyandrost-5-ene-17-one (**7β-OH-DHEA**) ($CDCl_3$, 151 MHz)

Fig.S28. HSQC spectral of 3β,7β-dihydroxyandrost-5-ene-17-one (**7β-OH-DHEA**) ($CDCl_3$, 151 MHz)

Fig.S29. ^{1}H NMR spectral of 3β-hydroxyandrost-5-ene-7,17-dione (**7-oxo-DHEA**) ($CDCl_3$, 600 MHz)

Fig.S30. ^{13}C NMR spectral of 3β-hydroxyandrost-5-ene-7,17-dione (**7-oxo-DHEA**) ($CDCl_3$, 151 MHz)

Fig.S31. HSQC spectral of 3β-hydroxyandrost-5-ene-7,17-dione (**7-oxo-DHEA**) ($CDCl_3$, 151 MHz)

Fig.S32. COSY spectral of 3β-hydroxyandrost-5-ene-7,17-dione (**7-oxo-DHEA**) ($CDCl_3$, 151 MHz)

Fig.S33. ^{1}H NMR spectral of 3β,7α-dihydroxy-17a-oxa-D-homo-androst-5-en-17-one (**7α-OH-DHEA-lactone**) ($CDCl_3$, 600 MHz)

Fig.S34. ^{13}C NMR spectral of 3β,7α-dihydroxy-17a-oxa-D-homo-androst-5-en-17-one (**7α-OH-DHEA-lactone**) ($CDCl_3$, 151 MHz)

Fig.S35. HSQC spectral of 3β,7α-dihydroxy-17a-oxa-D-homo-androst-5-en-17-one (**7α-OH-DHEA-lactone**) ($CDCl_3$, 151 MHz)

Fig.S36. ^{1}H NMR spectral of 3β,7β-dihydroxy-17a-oxa-D-homo-androst-5-en-17-one (**7β-OH-DHEA-lactone**) ($CDCl_3$, 600 MHz)

Fig.S37. ^{13}C NMR spectral of 3β,7β-dihydroxy-17a-oxa-D-homo-androst-5-en-17-one (**7β-OH-DHEA-lactone**) ($CDCl_3$, 151 MHz)

Fig.S38. HSQC spectral of 3β,7β-dihydroxy-17a-oxa-D-homo-androst-5-en-17-one (**7β-OH-DHEA-lactone**) ($CDCl_3$, 151 MHz)

Fig.S39. GC-MS spectra of 7α-hydroxyandrost-4-ene-3,17-dione (**7α-OH-AD**)

Fig.S40. Enlarged GC-MS spectra of 7α-hydroxyandrost-4-ene-3,17-dione (**7α-OH-AD**)

Fig.S41. GC-MS spectra of 6β-hydroxyandrost-4-ene-3,11,17-trione (**6β-OH-Adr**)

Fig.S42. Enlarged GC-MS spectra of 6β-hydroxyandrost-4-ene-3,11,17-trione (**6β-OH-Adr**)

Fig.S43. GC-MS spectra of 15β-hydroxy-17α-methyltestosterone (**15β-OH-17mT**)

Fig.S44. GC-MS spectra of 6β-hydroxy-17α-methyltestosterone (**6β-OH-17mT**)

Fig.S45. GC-MS spectra of 6β,12β-dihydroxy-17α-methyltestosterone (**6β,12β-OH-17mT**)

Fig.S46. Enlarged GC-MS spectra of 6β,12β-dihydroxy-17α-methyltestosterone (**6β,12β-OH-17mT**)

Fig.S47. GC-MS spectra of 3β,7α-dihydroxyandrost-5-ene-17-one (**7α-OH-DHEA**)

Fig.S48. Enlarged GC-MS spectra of 3β,7α-dihydroxyandrost-5-ene-17-one (**7α-OH-DHEA**)

Fig.S49. GC-MS spectra of 3β,7β-dihydroxyandrost-5-ene-17-one (**7β-OH-DHEA**)

Fig.S50. Enlarged GC-MS spectra of 3β,7β-dihydroxyandrost-5-ene-17-one (**7β-OH-DHEA**)

Fig.S51. GC-MS spectra of 3β-hydroxyandrost-5-ene-7,17-dione (**7-oxo-DHEA**)

Fig.S52. Enlarged GC-MS spectra of 3β-hydroxyandrost-5-ene-7,17-dione (**7-oxo-DHEA**)

Fig.S53. GC-MS spectra of 3β,7α-dihydroxy-17a-oxa-D-homo-androst-5-en-17-one (**7α-OH-DHEA-lactone**)

Fig.S54. Enlarged GC-MS spectra of 3β,7α-dihydroxy-17a-oxa-D-homo-androst-5-en-17-one (**7α-OH-DHEA-lactone**)

Fig.S55. GC-MS spectra of 3β,7β-dihydroxy-17a-oxa-D-homo-androst-5-en-17-one (**7β-OH-DHEA-lactone**)

Fig.S56. Enlarged GC-MS spectra of 3β,7β-dihydroxy-17a-oxa-D-homo-androst-5-en-17-one (**7β-OH-DHEA-lactone**)

Fig.S1. ^{1}H NMR spectral of 7α-hydroxyandrost-4-ene-3,17-dione (**7α-OH-AD**) ($CDCl_3$, 600 MHz)

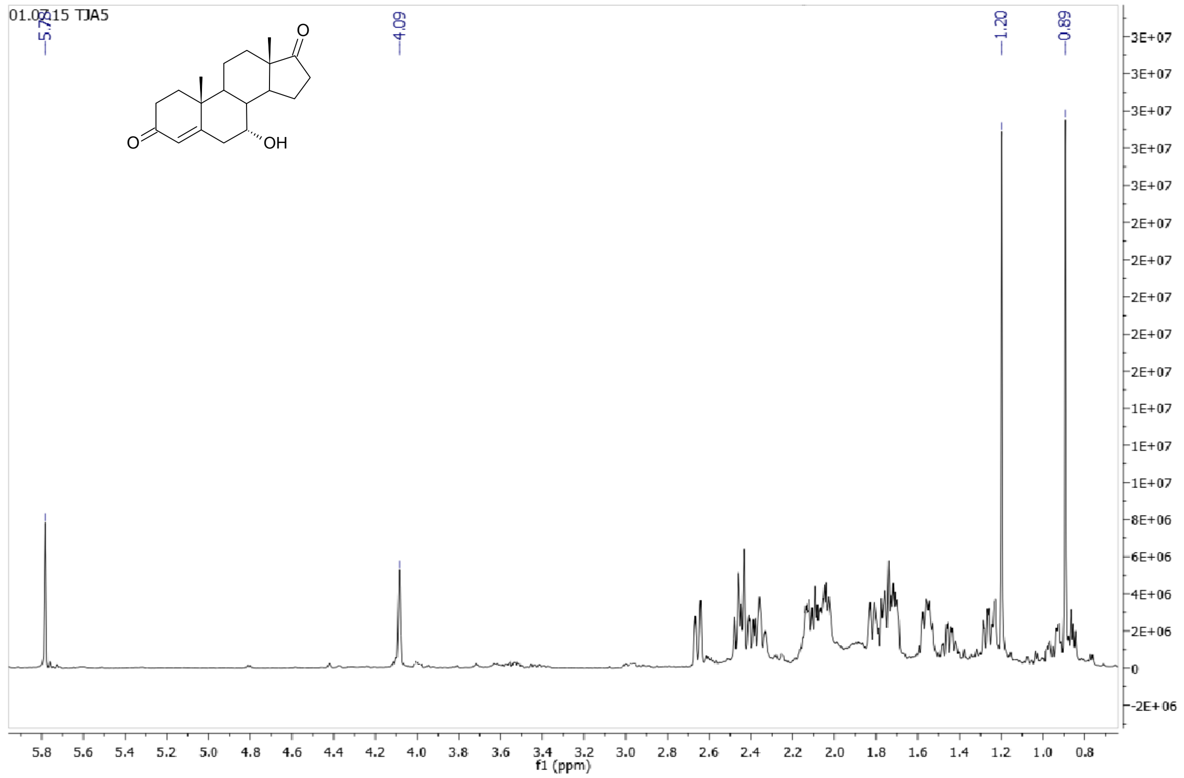

Fig.S2. ^{13}C NMR spectral of 7α-hydroxyandrost-4-ene-3,17-dione (**7α-OH-AD**) ($CDCl_3$, 151 MHz)

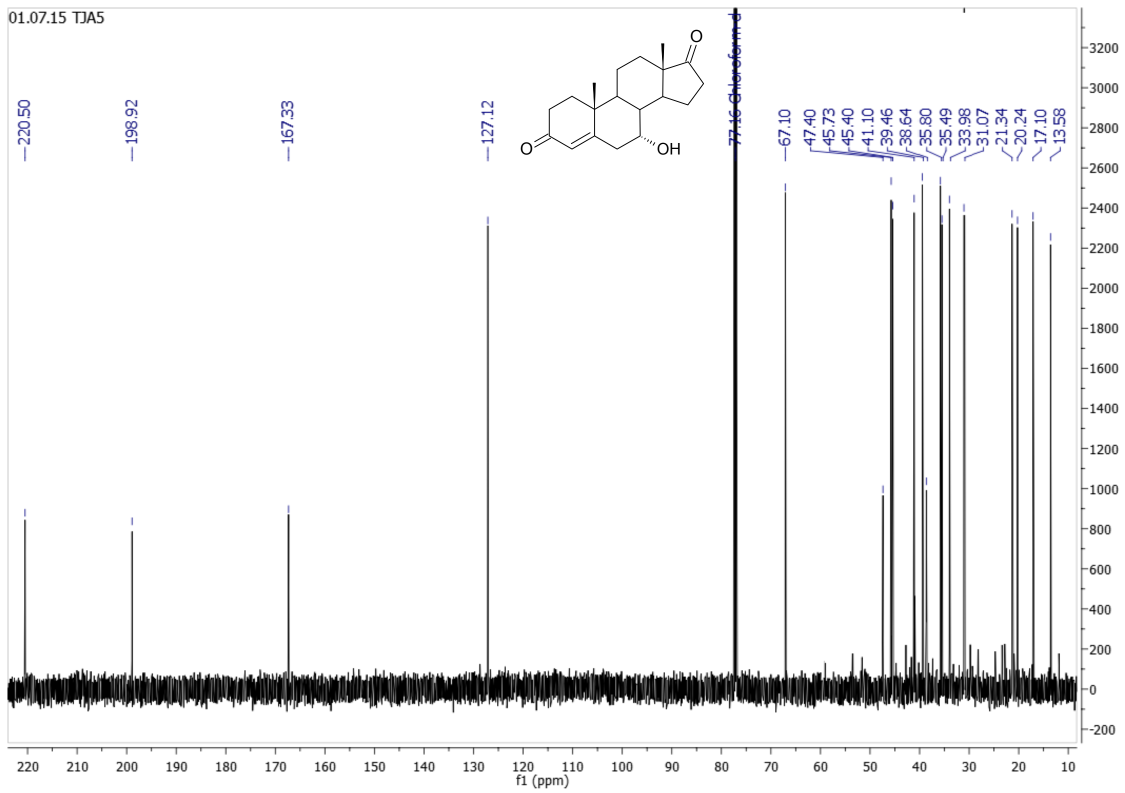

Fig.S3. HSQC spectral of 7α-hydroxyandrost-4-ene-3,17-dione (**7α-OH-AD**) (CDCl₃, 151 MHz)

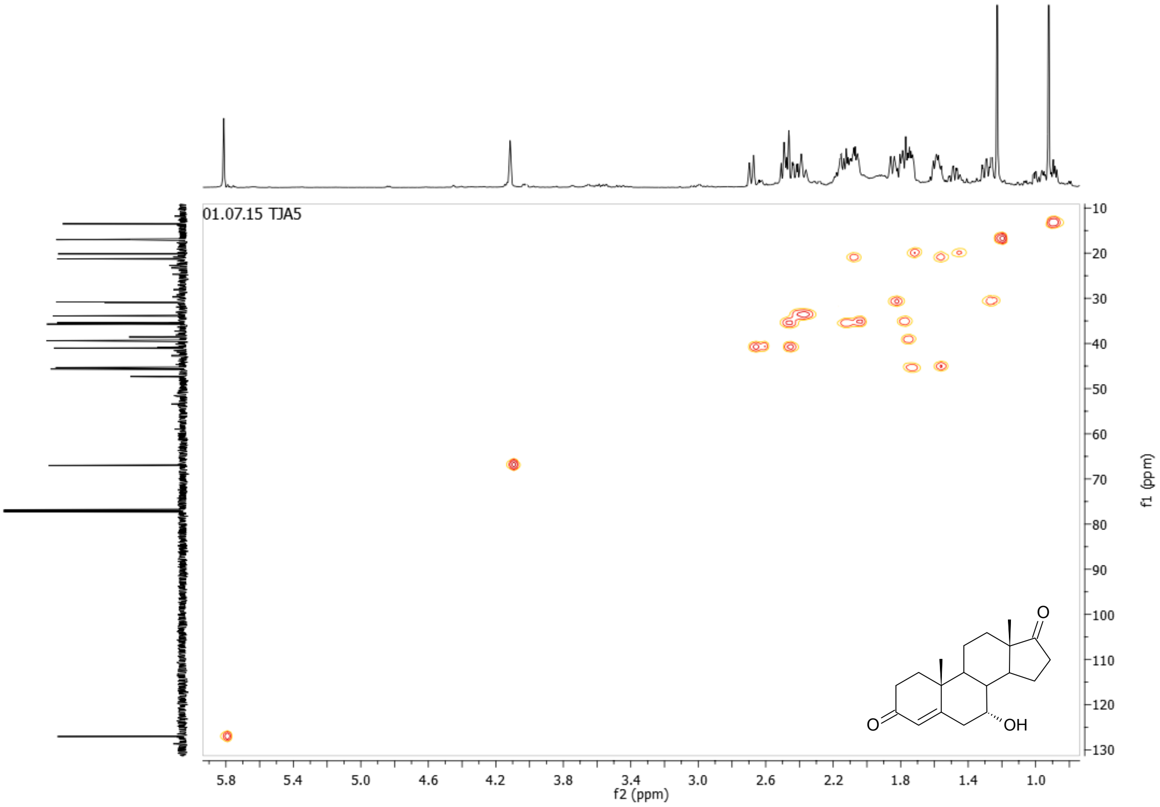

Fig.S4. COSY spectral of 7α-hydroxyandrost-4-ene-3,17-dione (**7α-OH-AD**) (CDCl₃, 151 MHz)

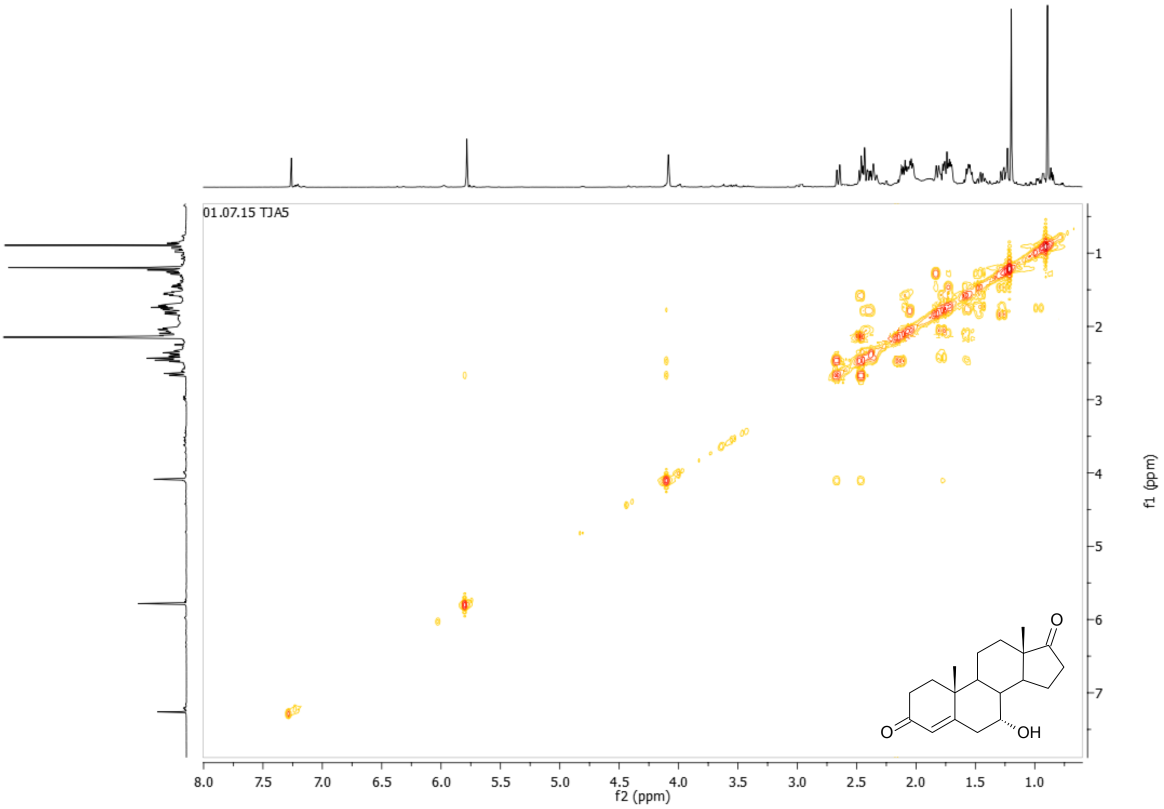

Fig.S5. ^{1}H NMR spectral of 6β-hydroxyandrost-4-ene-3,11,17-trione (**6β-OH-Adr**) (CDCl₃, 600 MHz)

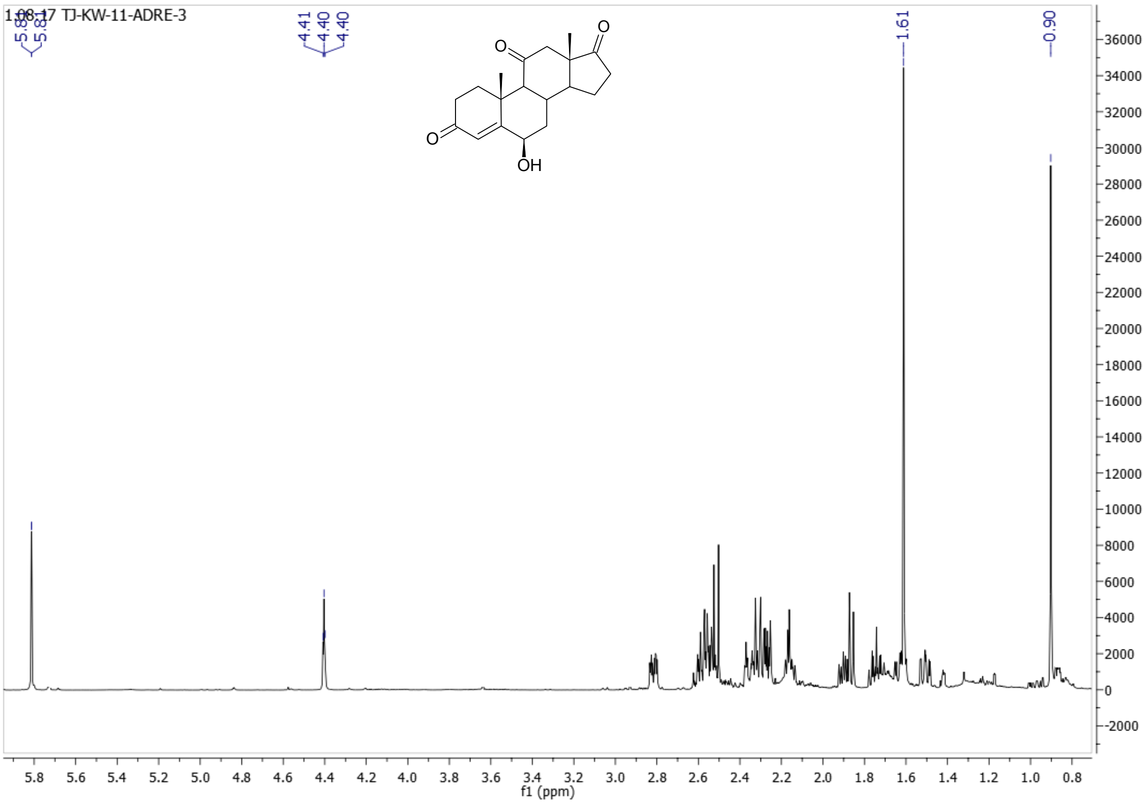

Fig.S6. ^{13}C NMR spectral of 6β-hydroxyandrost-4-ene-3,11,17-trione (**6β-OH-Adr**) (CDCl₃, 151 MHz)

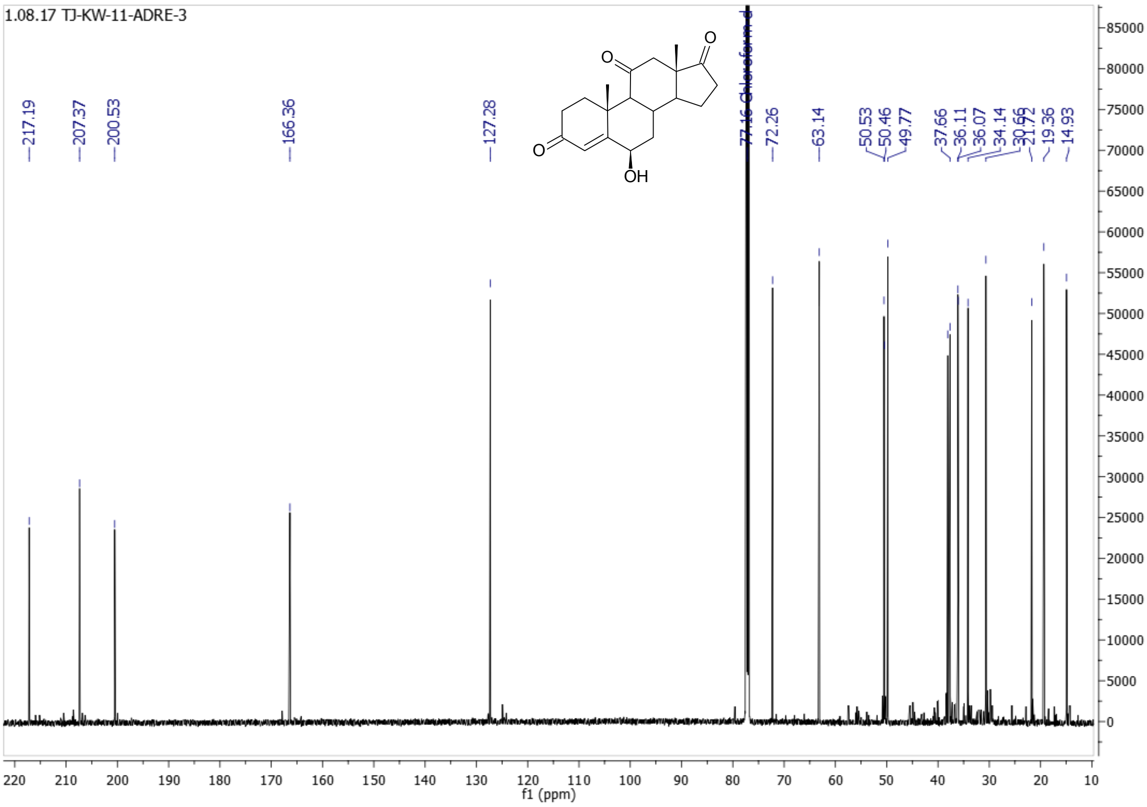

Fig.S7. HSQC spectral of 6β-hydroxyandrost-4-ene-3,11,17-trione (**6β-OH-Adr**) ($CDCl_3$, 151 MHz)

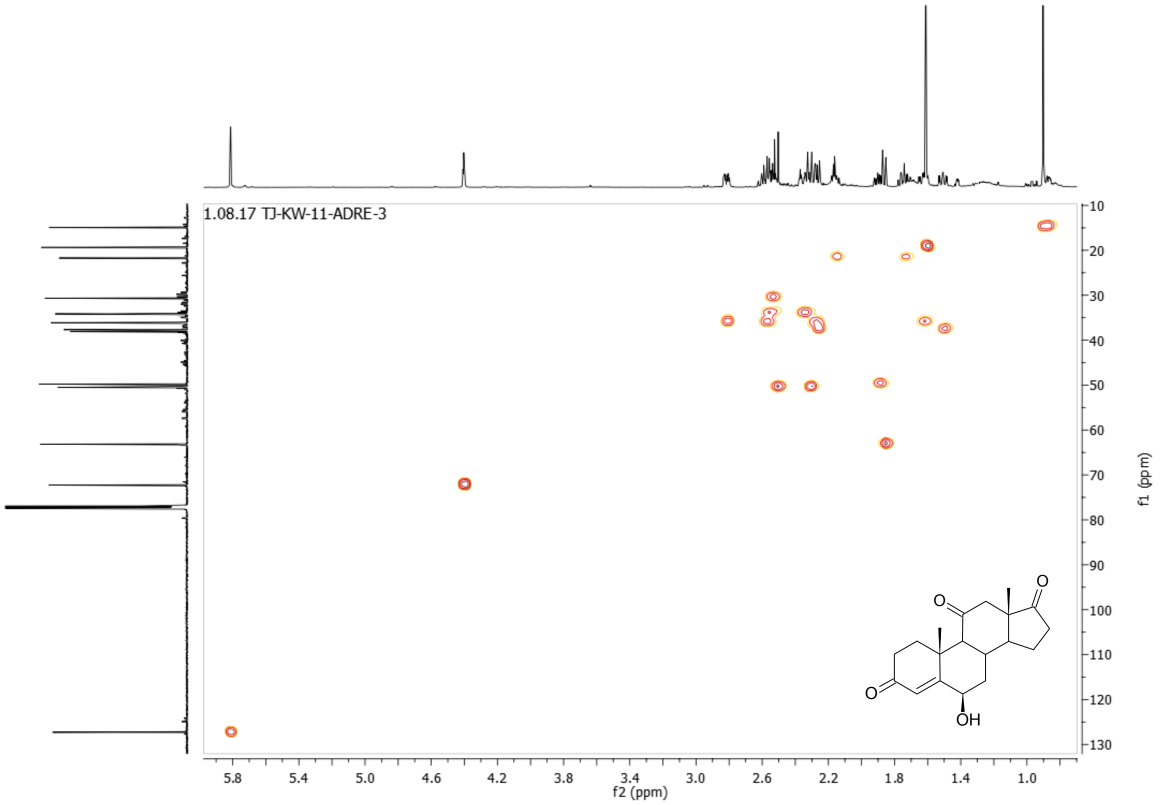

Fig.S8. COSY spectral of 6β-hydroxyandrost-4-ene-3,11,17-trione (**6β-OH-Adr**) ($CDCl_3$, 151 MHz)

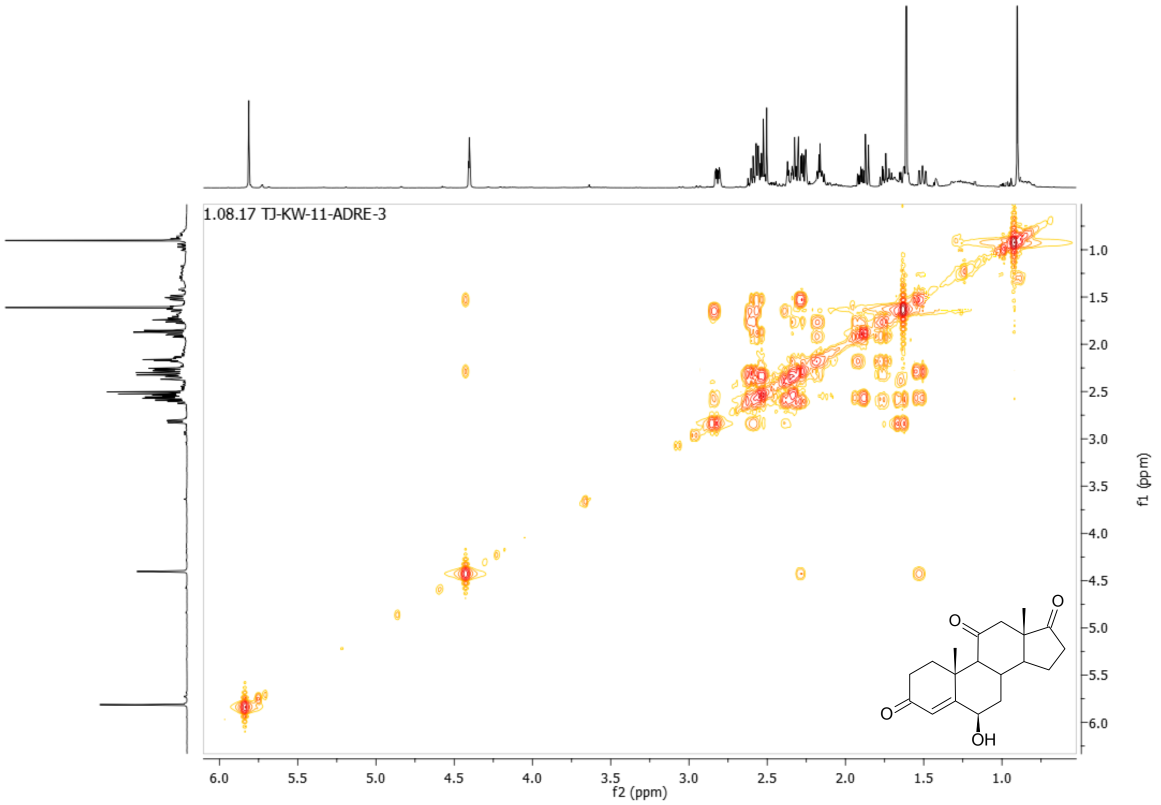

Fig.S9. ^{1}H NMR spectral of 15β-hydroxy-17α-methyltestosterone (**15β-OH-mT**) ($CDCl_3$, 600 MHz)

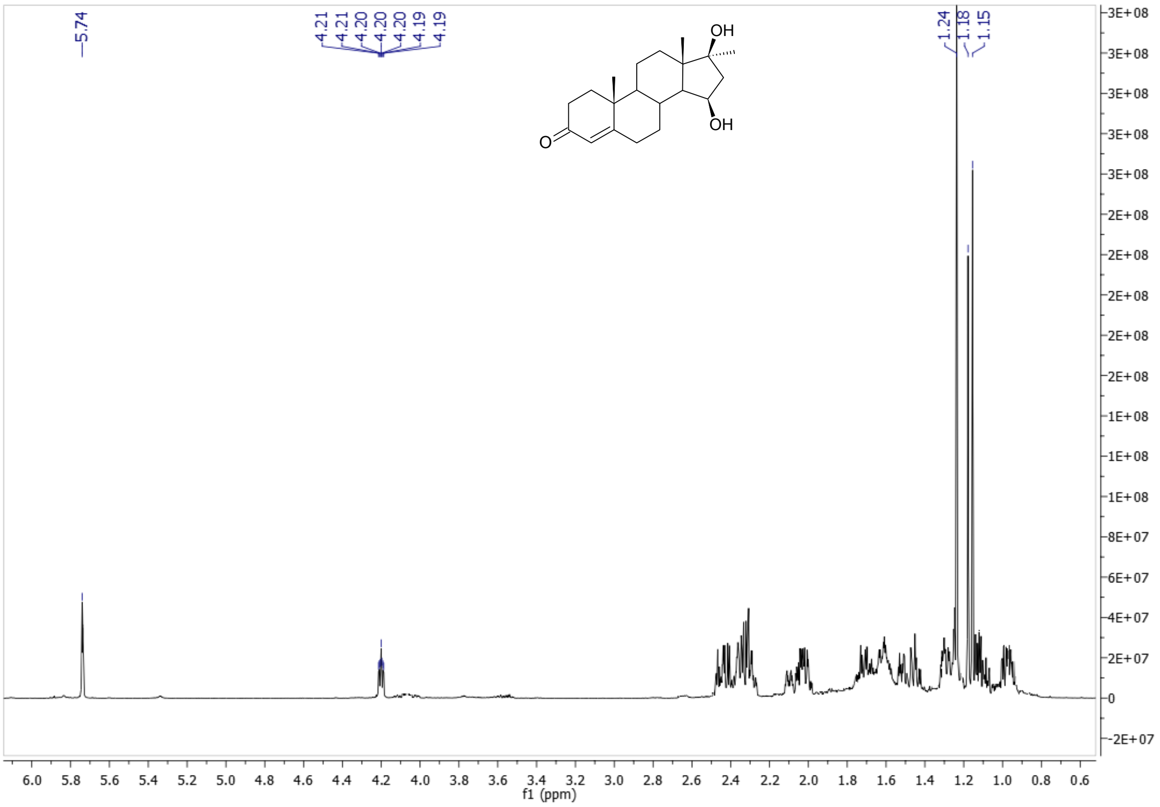

Fig.S10. ^{13}C NMR spectral of 15β-hydroxy-17α-methyltestosterone (**15β-OH-mT**) ($CDCl_3$, 151 MHz)

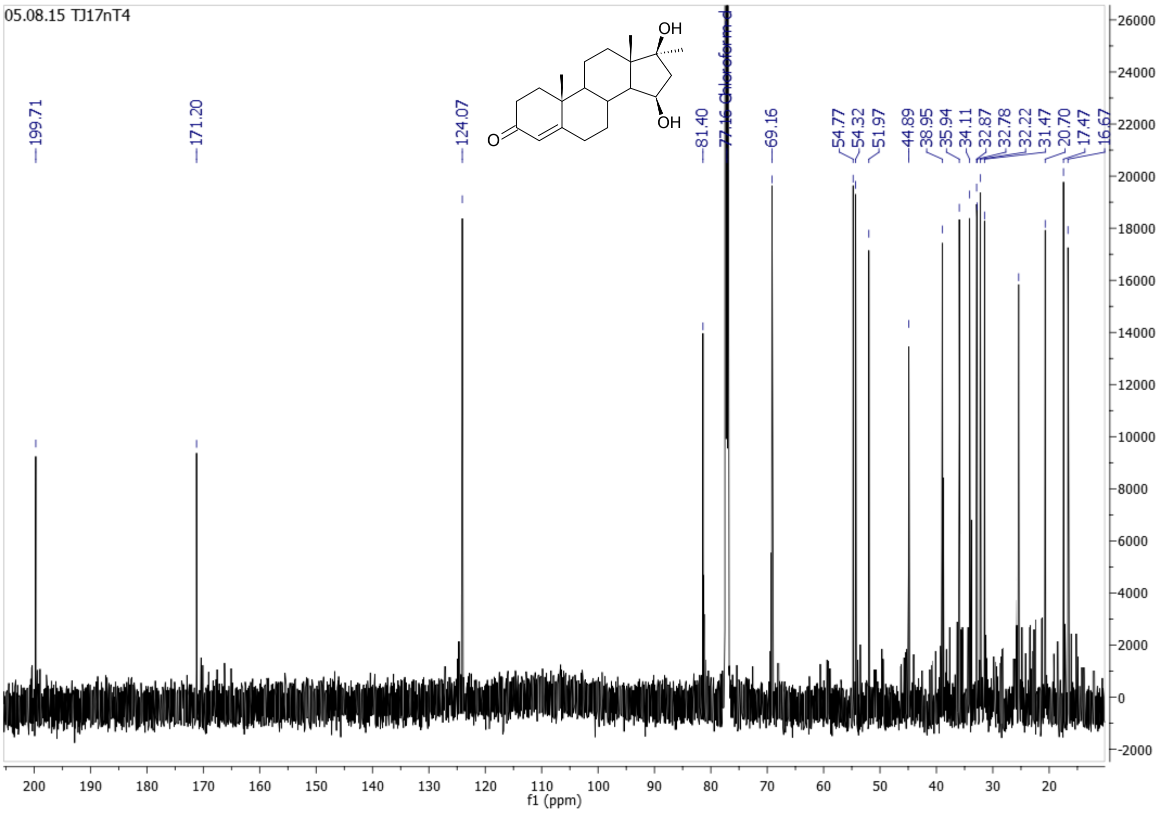

Fig.S11. ^{1}H NMR spectral of 6β-hydroxy-17α-methyltestosterone (**6β-OH-mT**) ($CDCl_3$, 600 MHz)

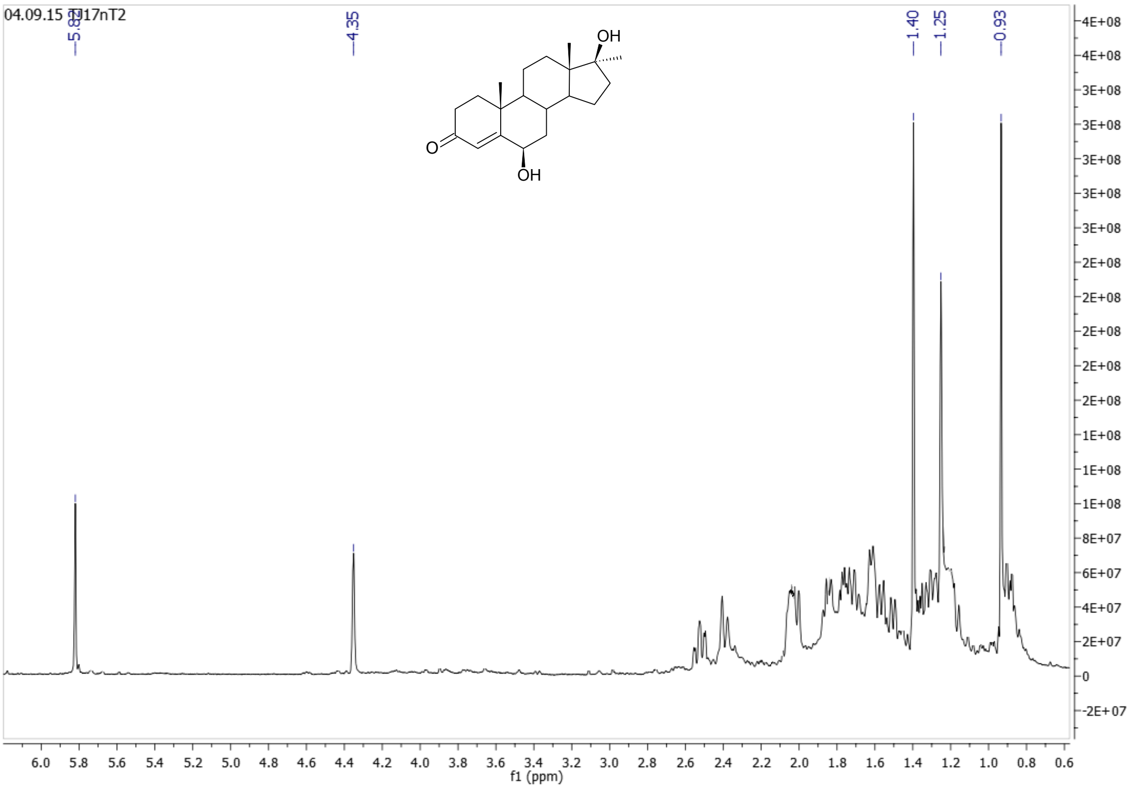

Fig.S12. ^{13}C NMR spectral of 6β-hydroxy-17α-methyltestosterone (**6β-OH-mT**) ($CDCl_3$, 151 MHz)

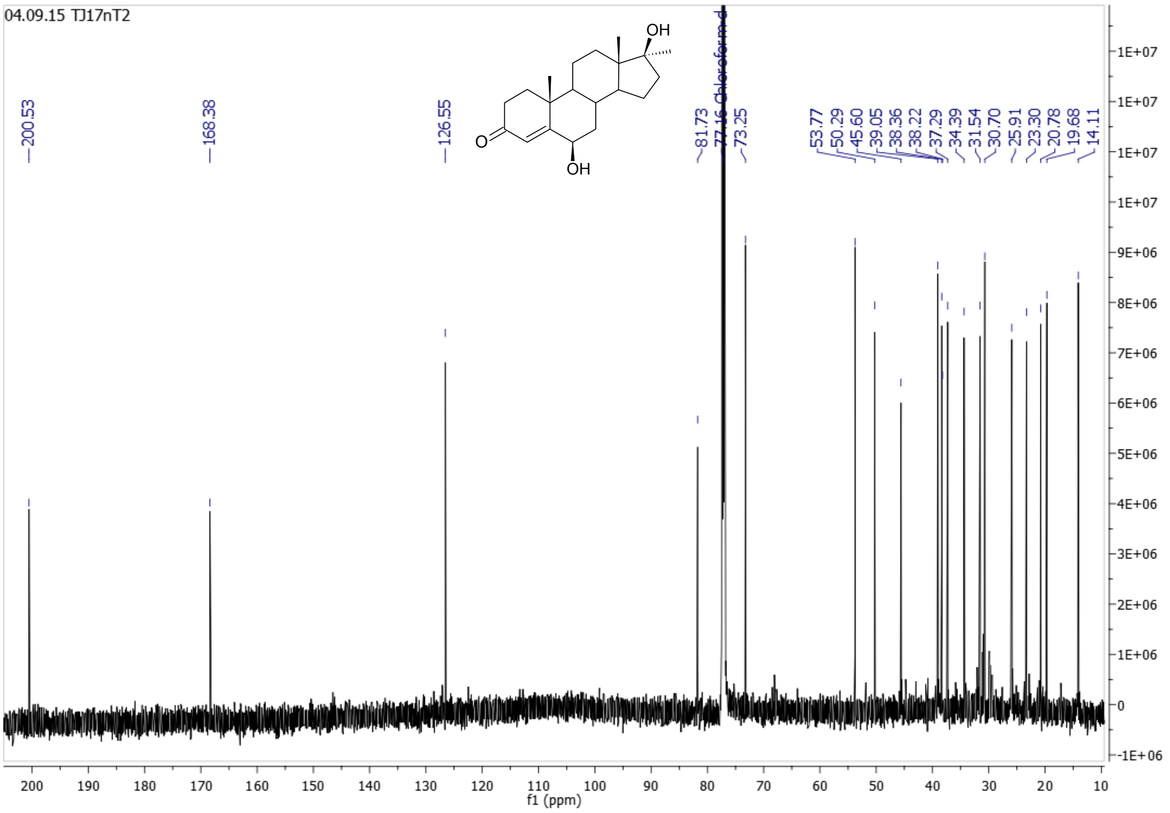

Fig.S13. ^{1}H NMR spectral of 6β-hydroxy-17α-methyltestosterone (**6β-OH-mT**) (THF, 600 MHz)

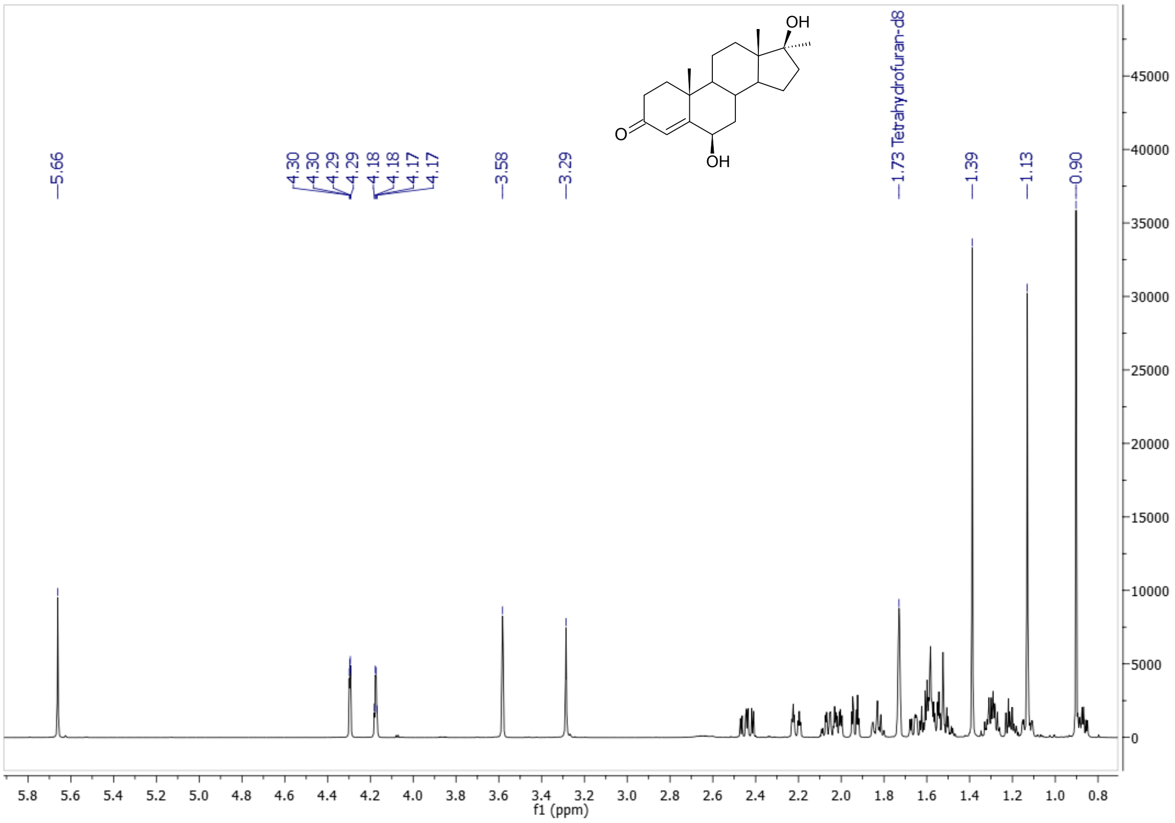

Fig.S14. ^{13}C NMR spectral of 6β-hydroxy-17α-methyltestosterone (**6β-OH-mT**) (THF, 151 MHz)

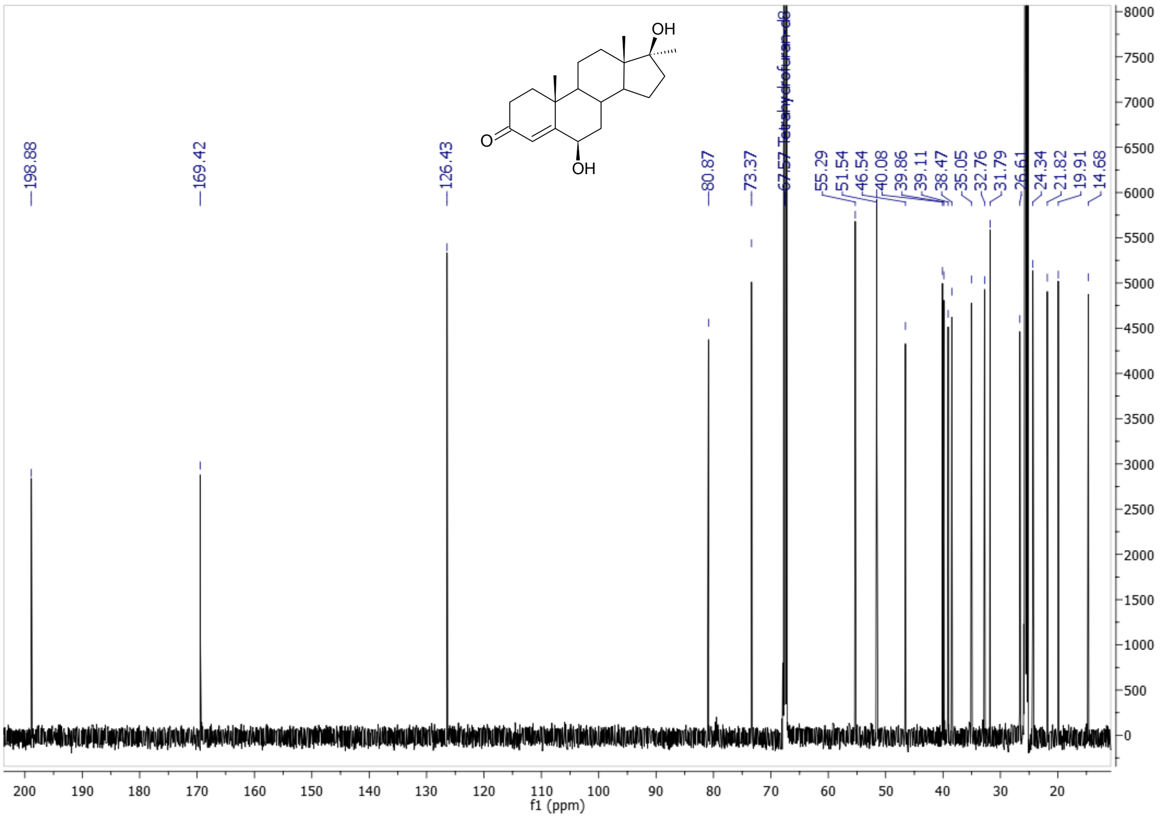

Fig.S15. HSQC spectral of 6β-hydroxy-17α-methyltestosterone (**6β-OH-mT**) (THF, 151 MHz)

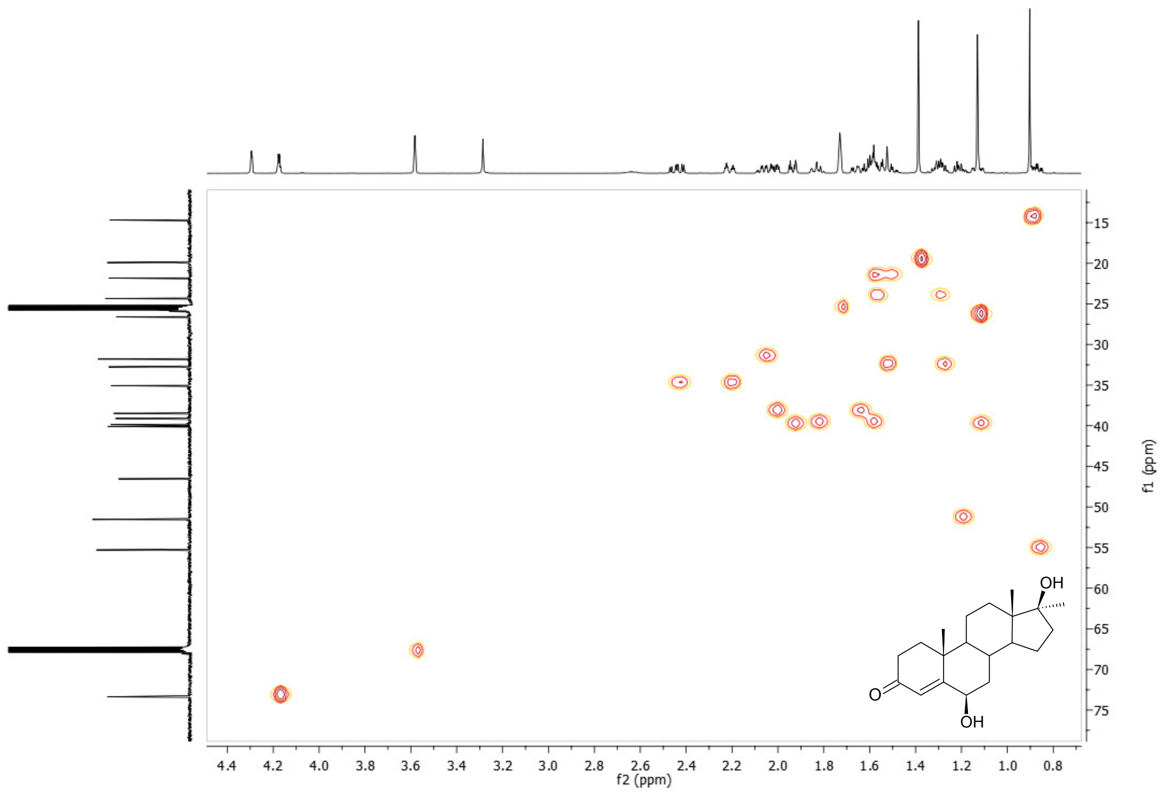

Fig.S16. COSY spectral of 6β-hydroxy-17α-methyltestosterone (**6β-OH-mT**) (THF, 151 MHz)

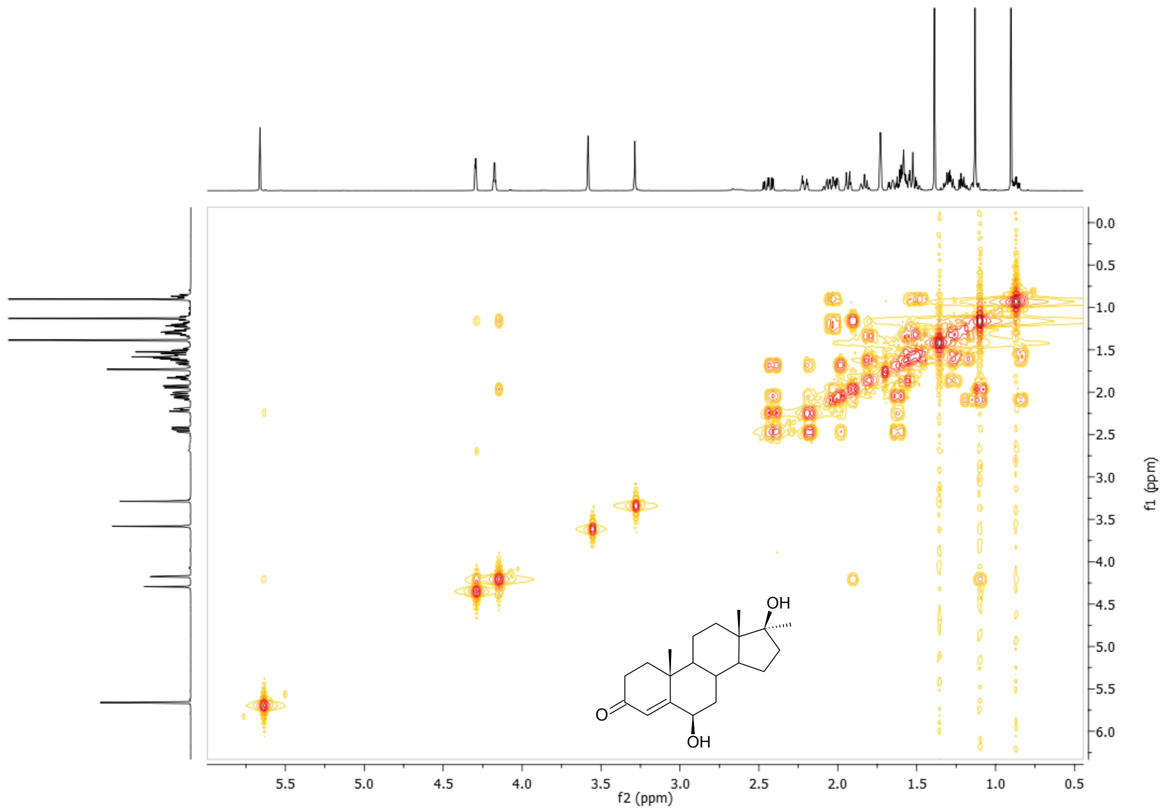

Fig.S17. ^{1}H NMR spectral of 6β,12β-dihydroxy-17α-methyltestosterone (**6β,12β-OH-mT**) ($CDCl_3$, 600 MHz)

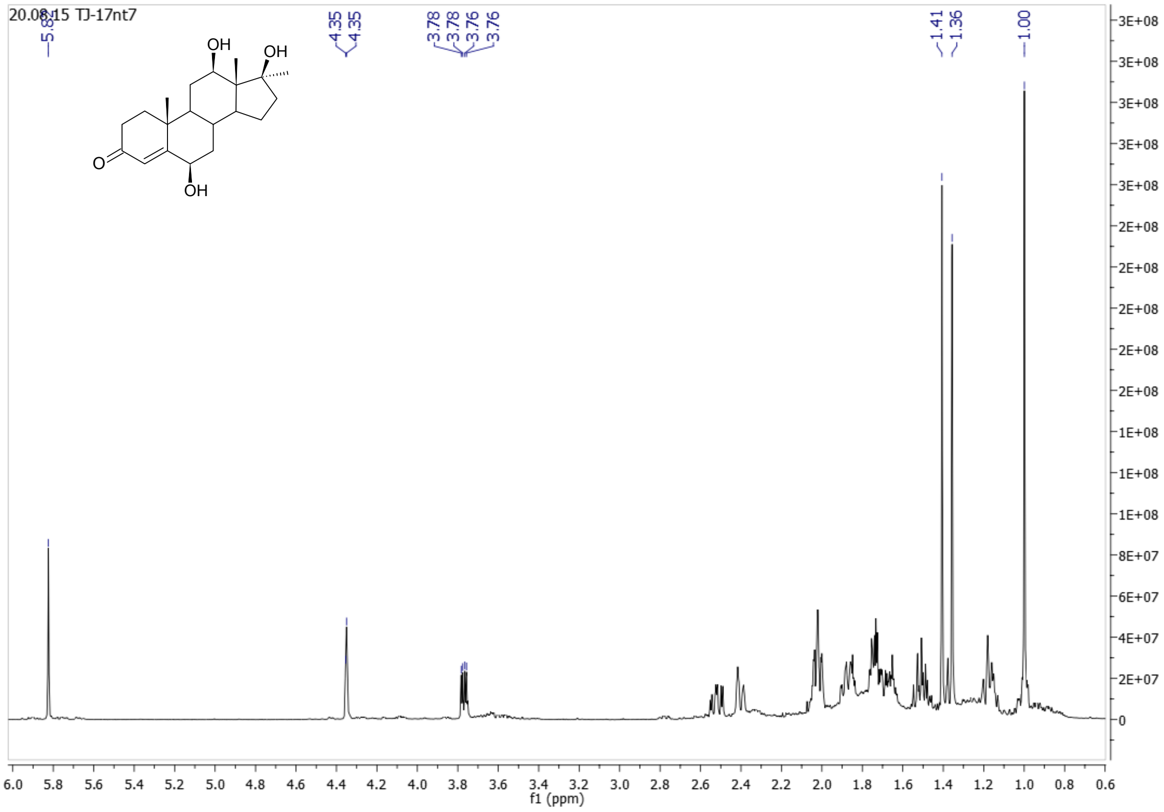

Fig.S18. ^{1}H NMR spectral of 6β,12β-dihydroxy-17α-methyltestosterone (**6β,12β-OH-mT**) (DMSO, 600 MHz)

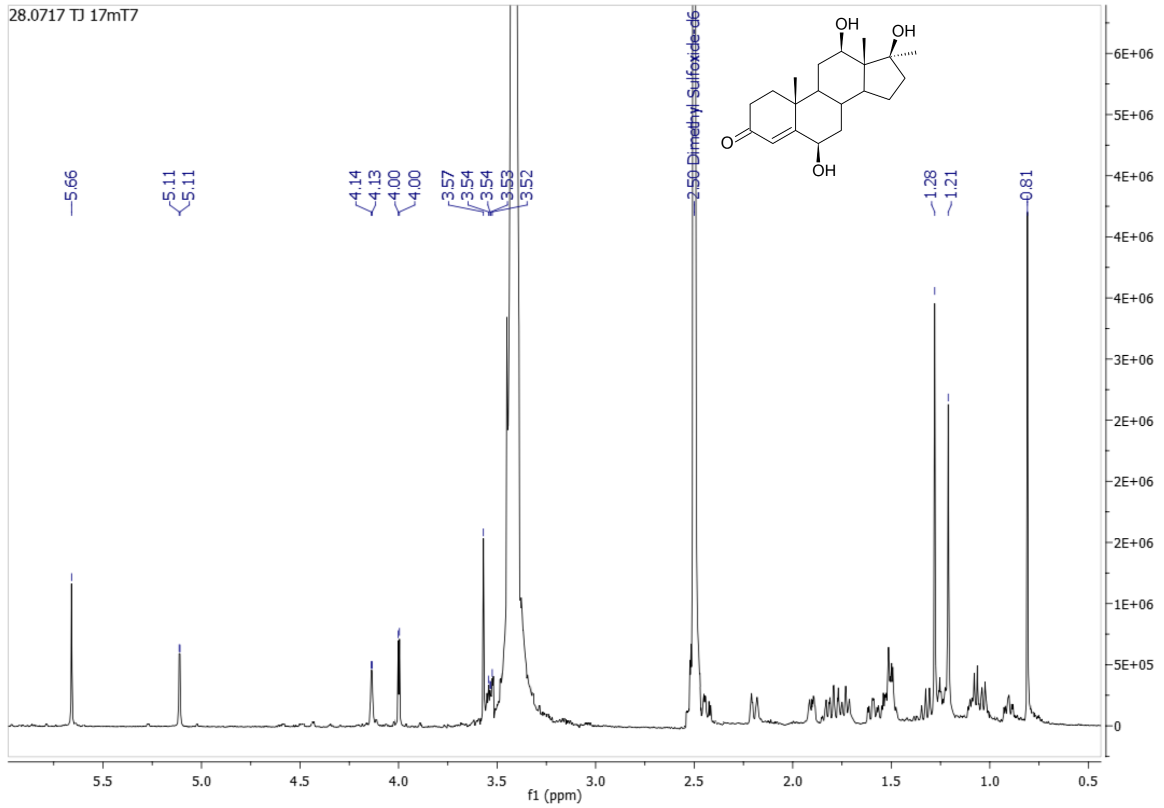

Fig.S19. ^{13}C NMR spectral of 6β,12β-dihydroxy-17α-methyltestosterone (**6β,12β-OH-mT**) (DMSO, 151 MHz)

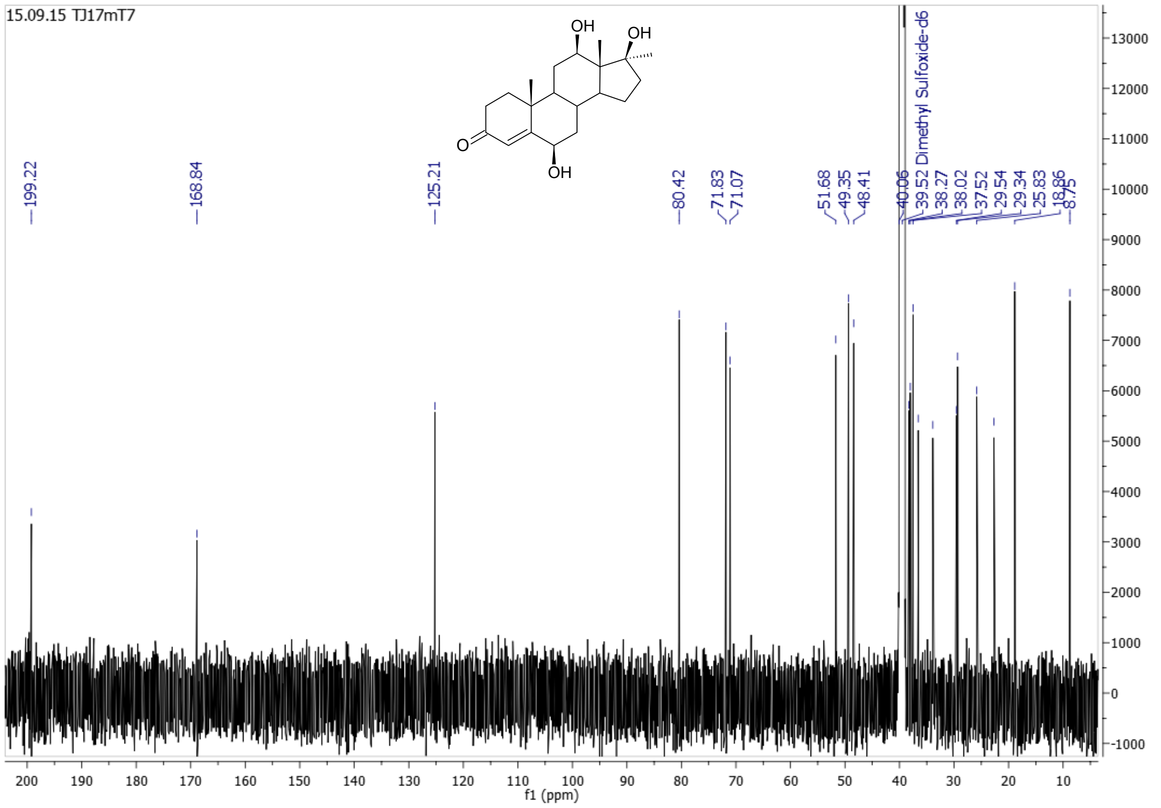

Fig.S20. HSQC spectral of 6β,12β-dihydroxy-17α-methyltestosterone (**6β,12β-OH-mT**) (DMSO, 151 MHz)

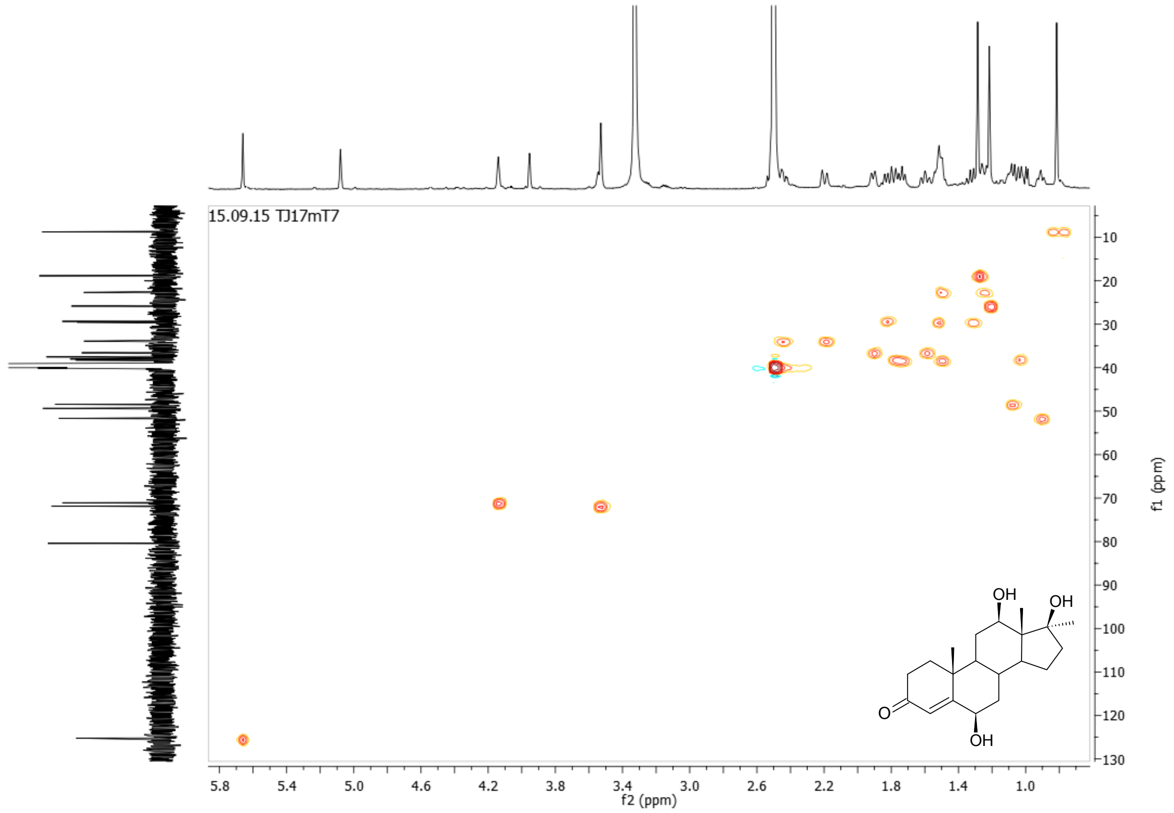

Fig.S21. COSY spectral of 6β,12β-dihydroxy-17α-methyltestosterone (**6β,12β-OH-mT**) (DMSO, 151 MHz)

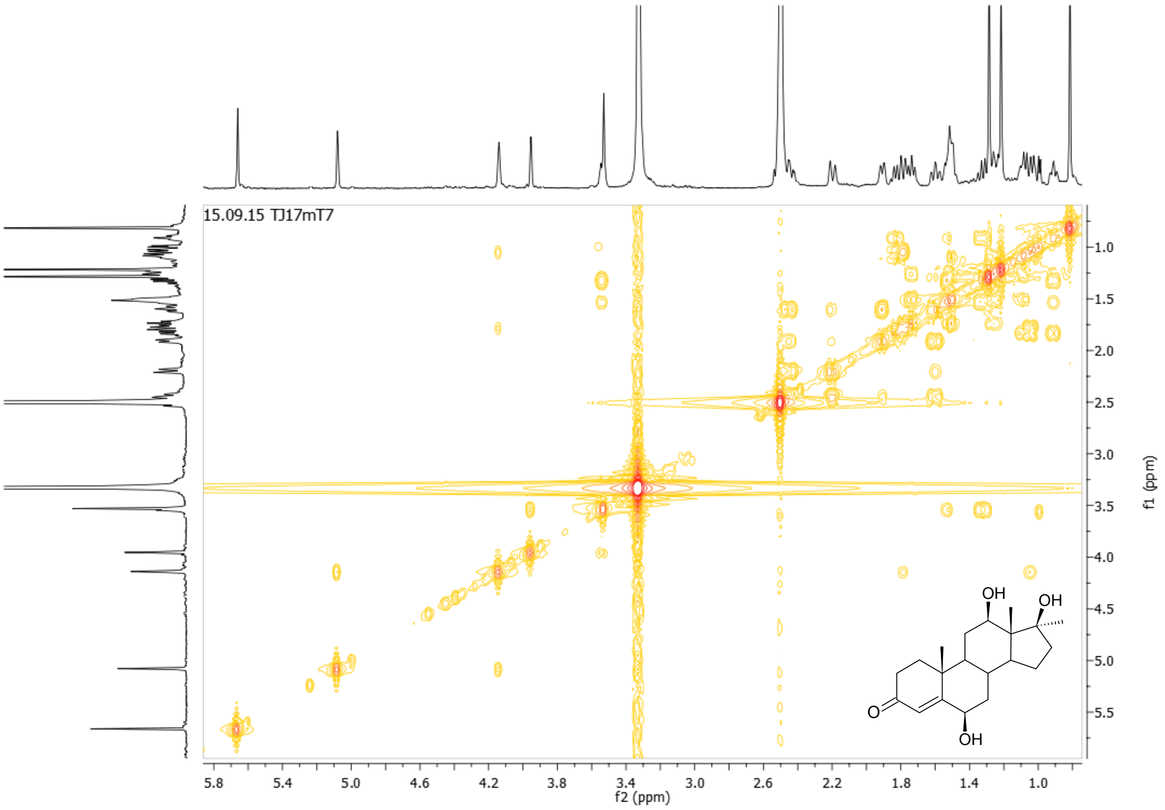

Fig.S22. ^{1}H NMR spectral of 3β,7α-dihydroxyandrost-5-ene-17-one (**7α-OH-DHEA**) ($CDCl_3$, 600 MHz)

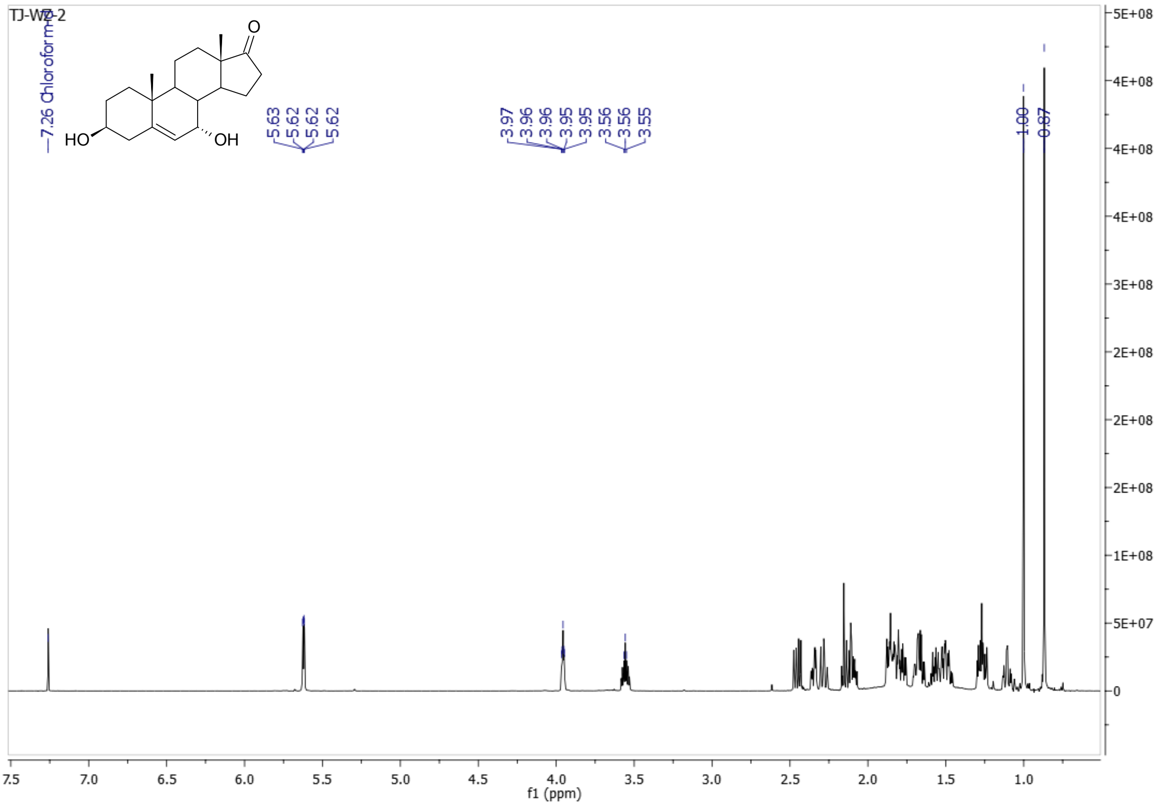

Fig.S23. ^{13}C NMR spectral of 3β,7α-dihydroxyandrost-5-ene-17-one (**7α-OH-DHEA**) ($CDCl_3$, 151 MHz)

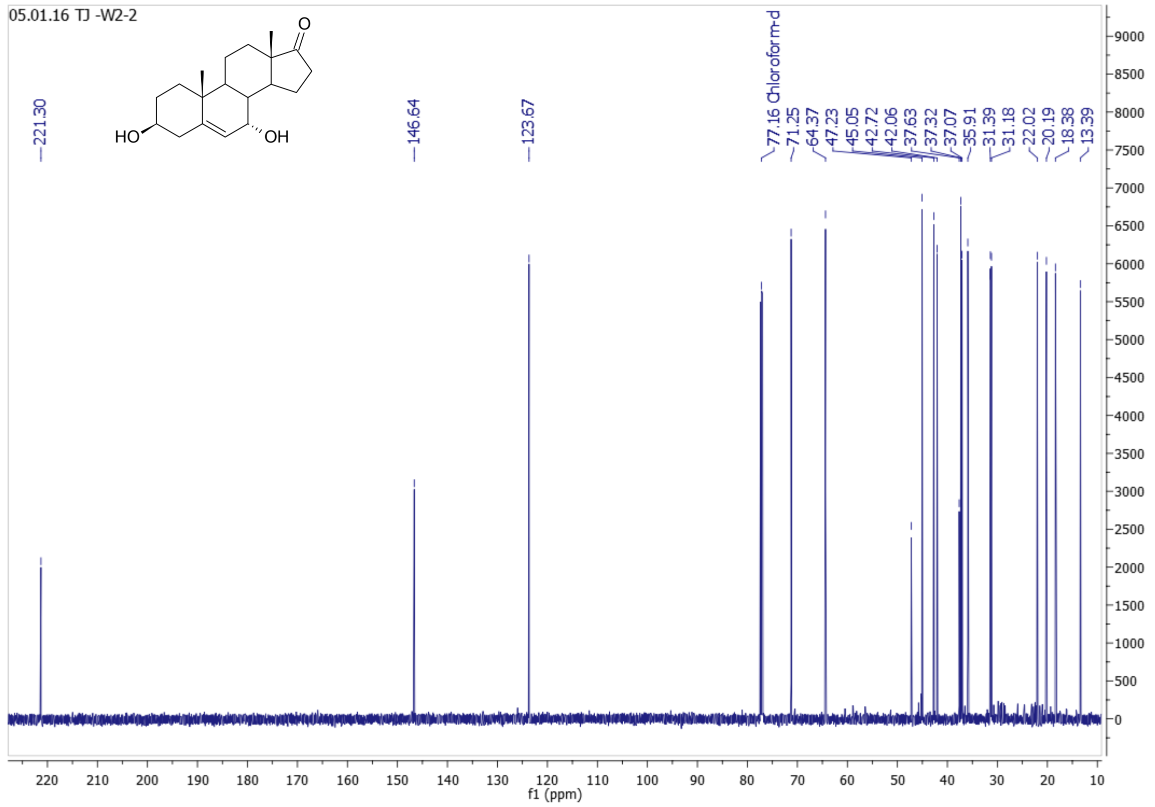

Fig.S24. HSQC spectral of 3β,7α-dihydroxyandrost-5-ene-17-one (**7αOH-DHEA**) ($CDCl_3$, 151 MHz)

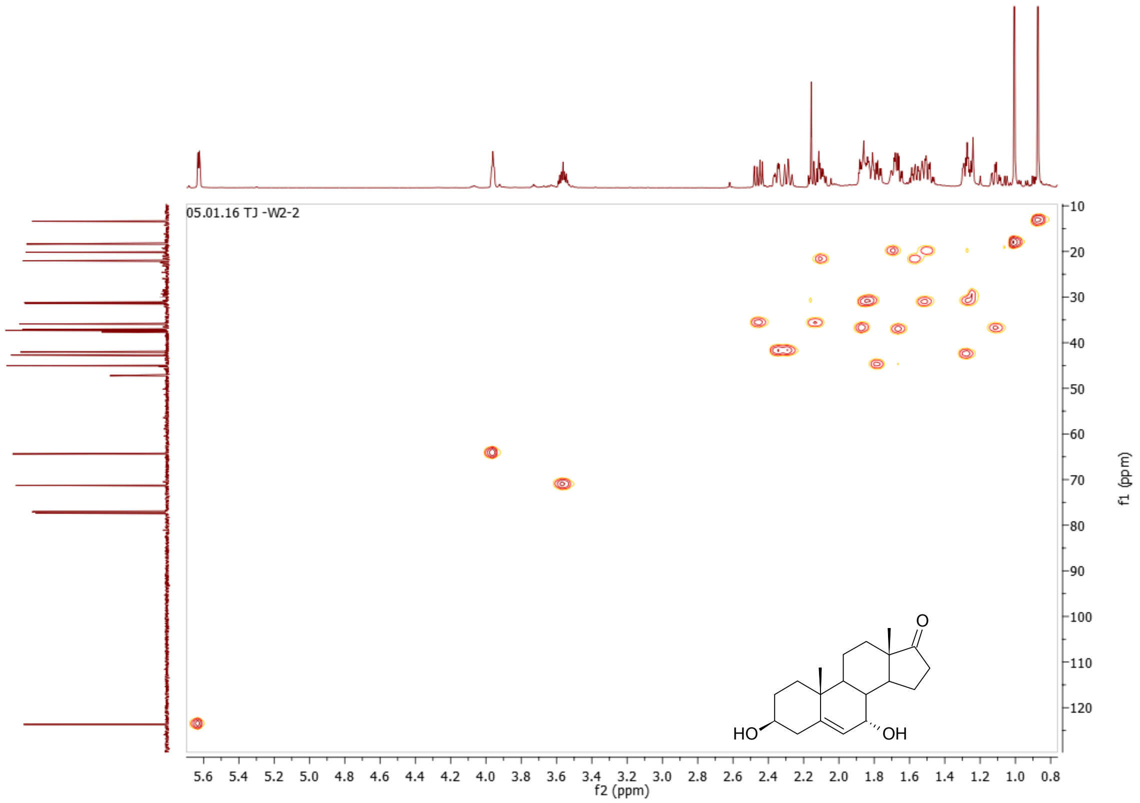

Fig.S25. COSY spectral of 3β,7α-dihydroxyandrost-5-ene-17-one (**7α-OH-DHEA**) ($CDCl_3$, 151 MHz)

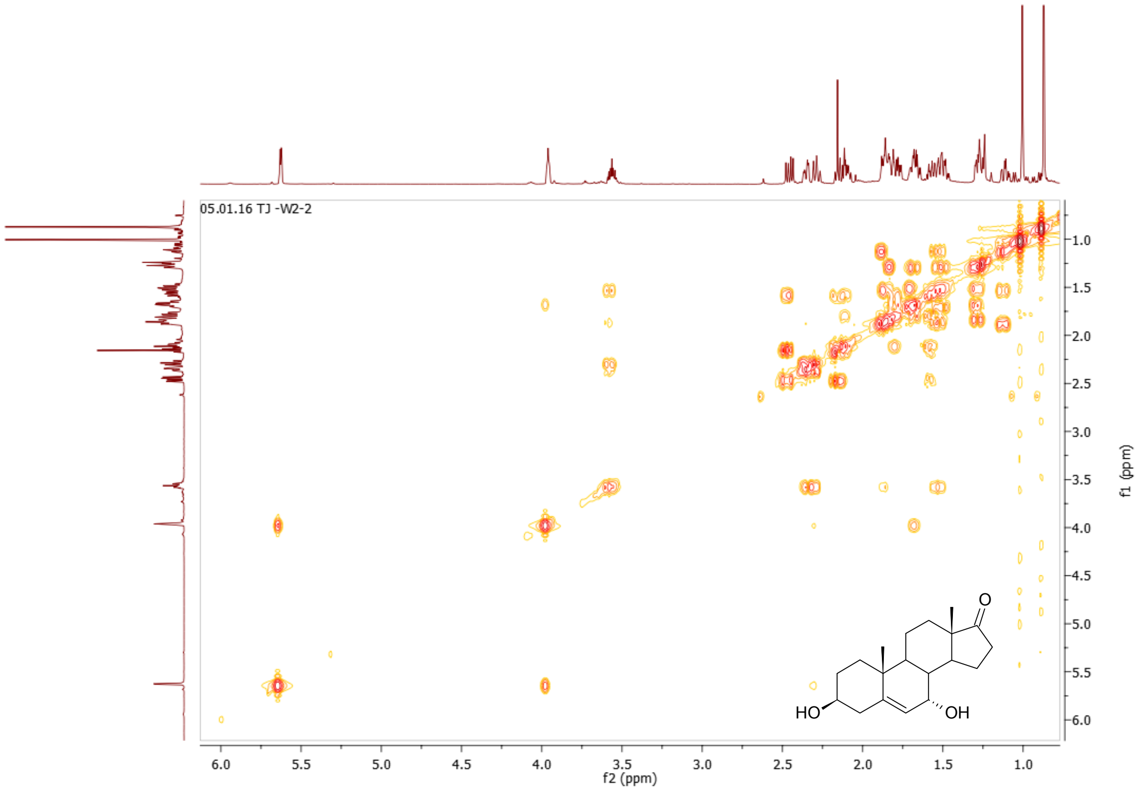

Fig.S26. ^{1}H NMR spectral of 3β,7β-dihydroxyandrost-5-ene-17-one (**7β-OH-DHEA**) ($CDCl_3$, 600 MHz)

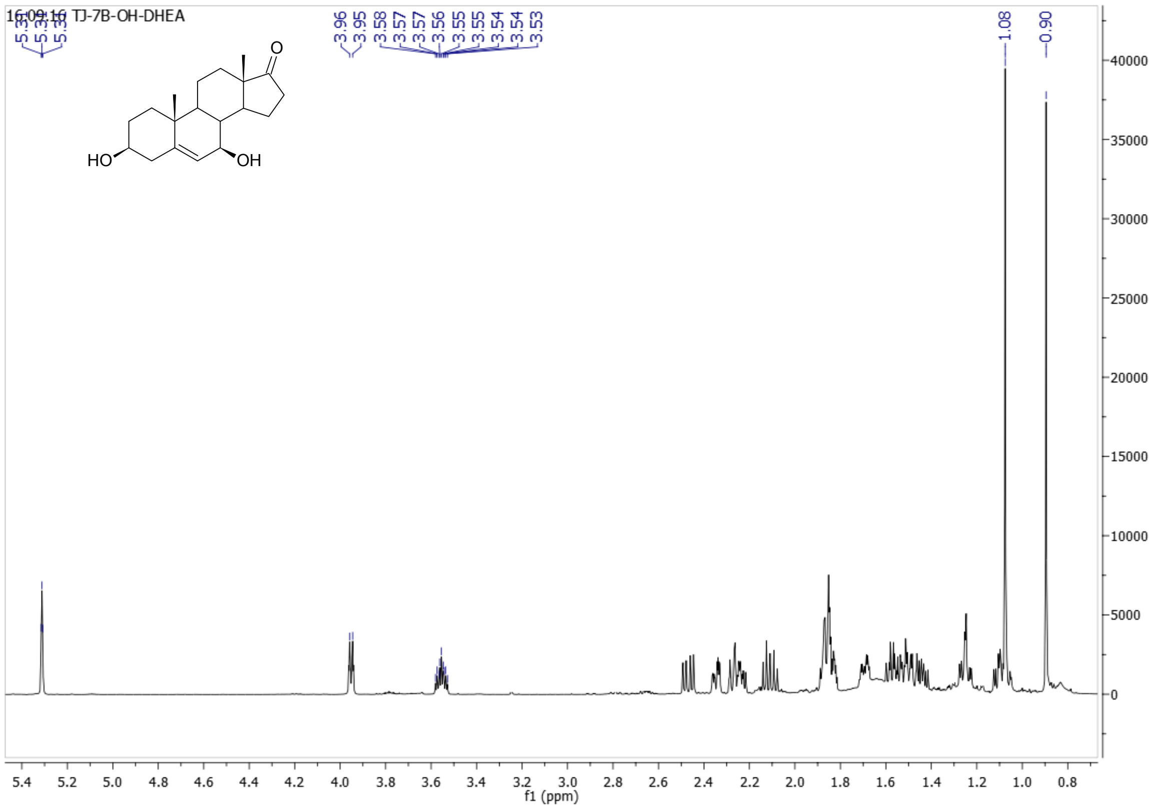

Fig.S27. ^{13}C NMR spectral of 3β,7β-dihydroxyandrost-5-ene-17-one (**7β-OH-DHEA**) ($CDCl_3$, 151 MHz)

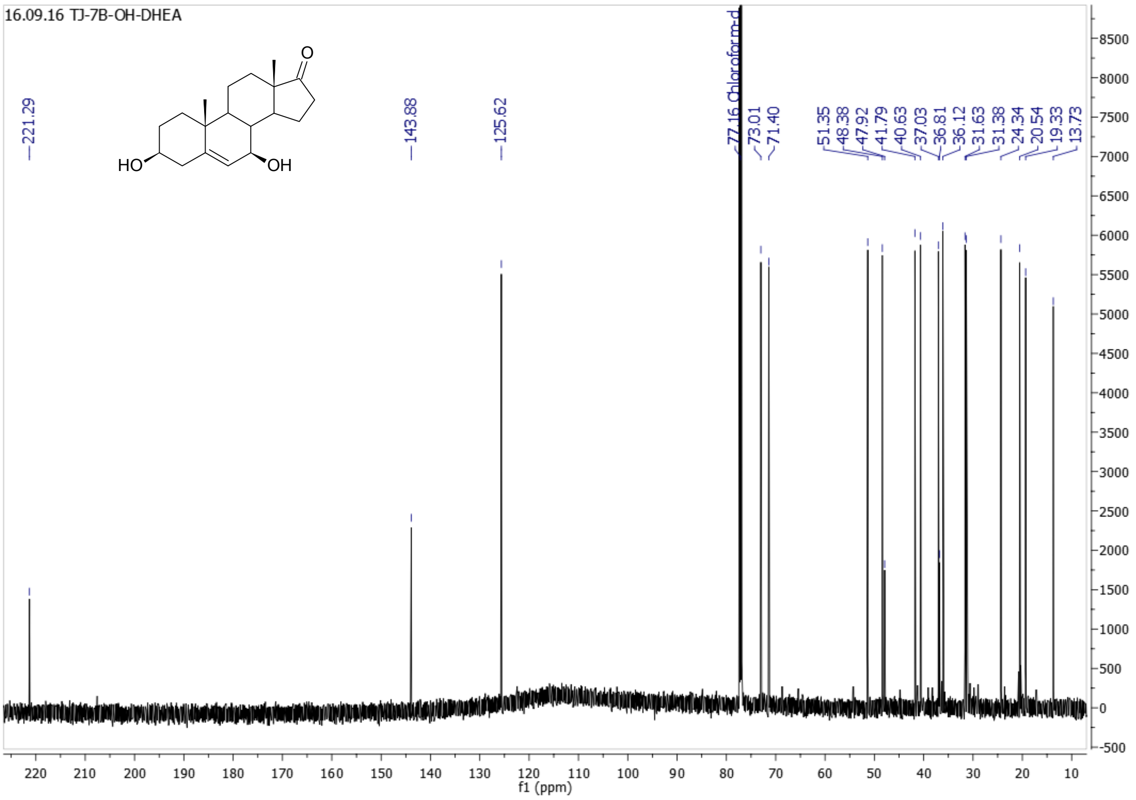

Fig.S28. HSQC spectral of 3β,7β-dihydroxyandrost-5-ene-17-one (**7β-OH-DHEA**) ($CDCl_3$, 151 MHz)

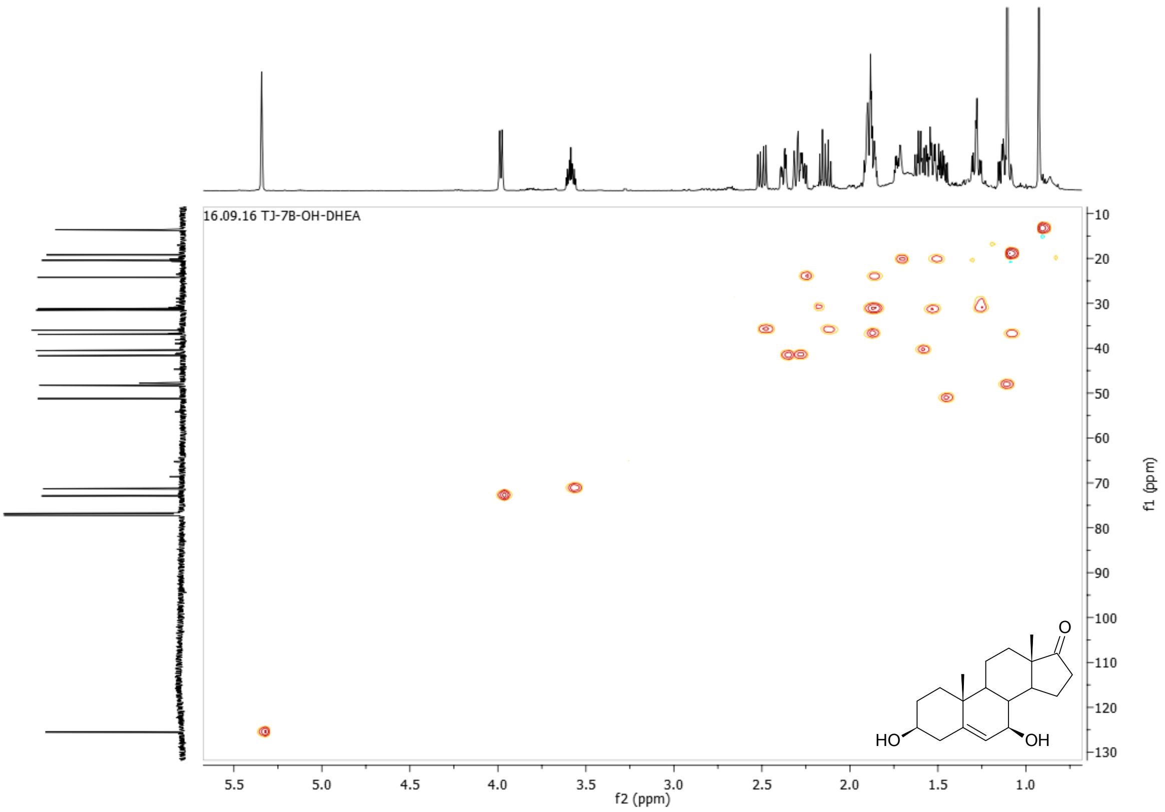

Fig.S29. ^1H NMR spectral of 3β-hydroxyandrost-5-ene-7,17-dione (**7-oxo-DHEA**) ($CDCl_3$, 600 MHz)

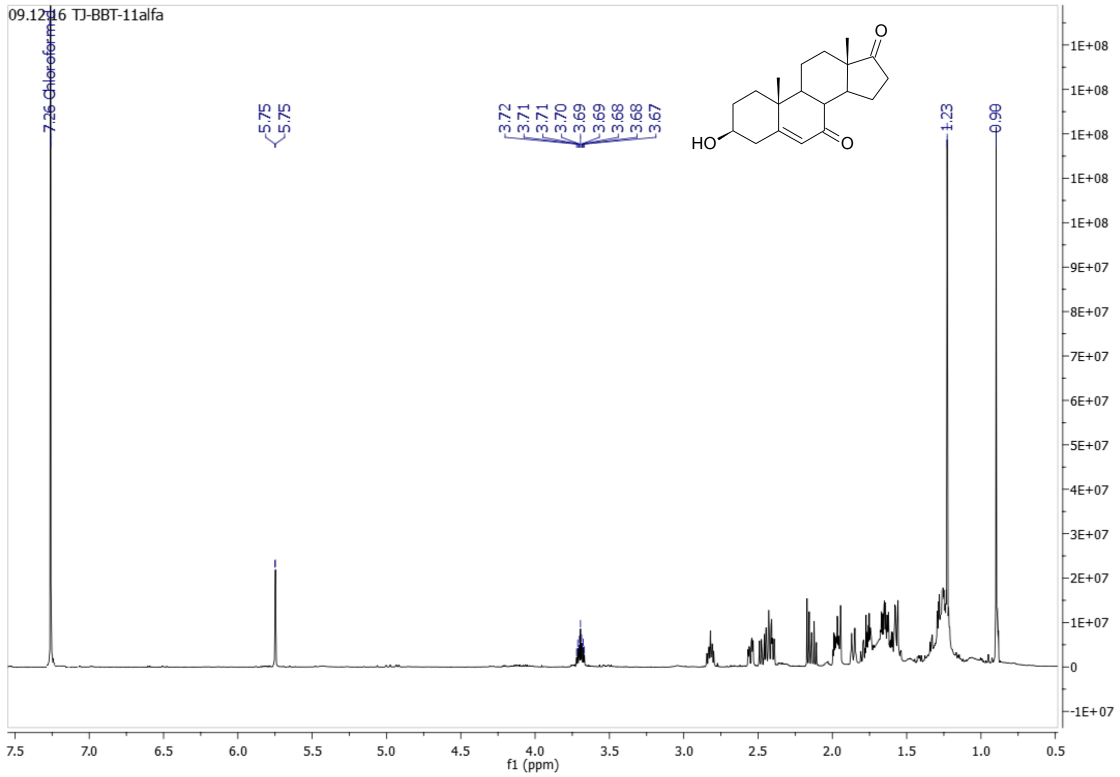

Fig.S30. ^{13}C NMR spectral of 3β-hydroxyandrost-5-ene-7,17-dione (**7-oxo-DHEA**) ($CDCl_3$, 151 MHz)

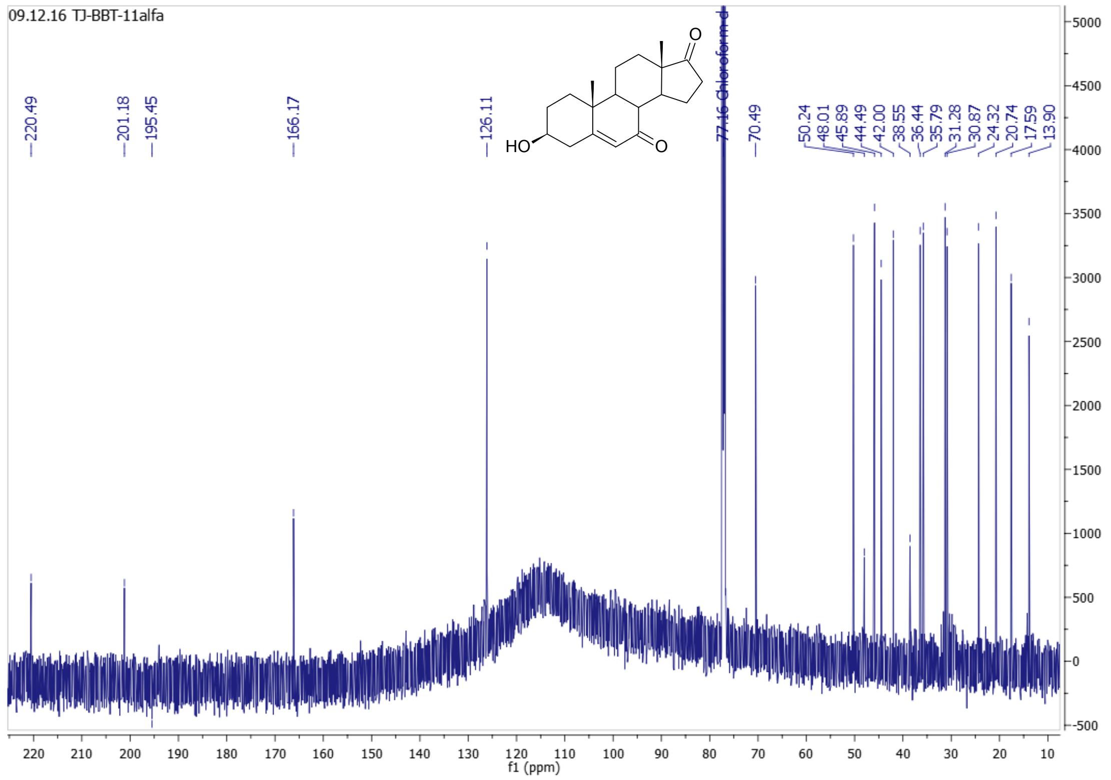

Fig.S31. HSQC spectral of 3β-hydroxyandrost-5-ene-7,17-dione (**7-oxo-DHEA**) ($CDCl_3$, 151 MHz)

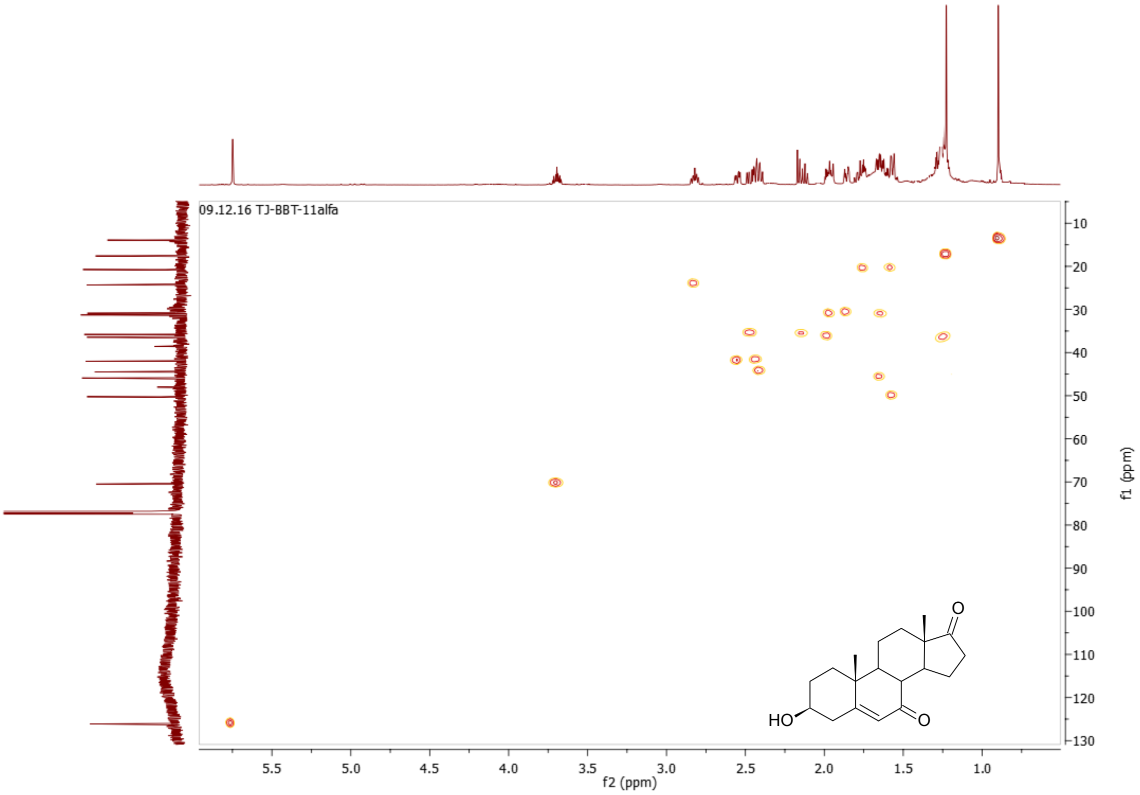

Fig.S32. COSY spectral of 3β-hydroxyandrost-5-ene-7,17-dione (**7-oxo-DHEA**) ($CDCl_3$, 151 MHz)

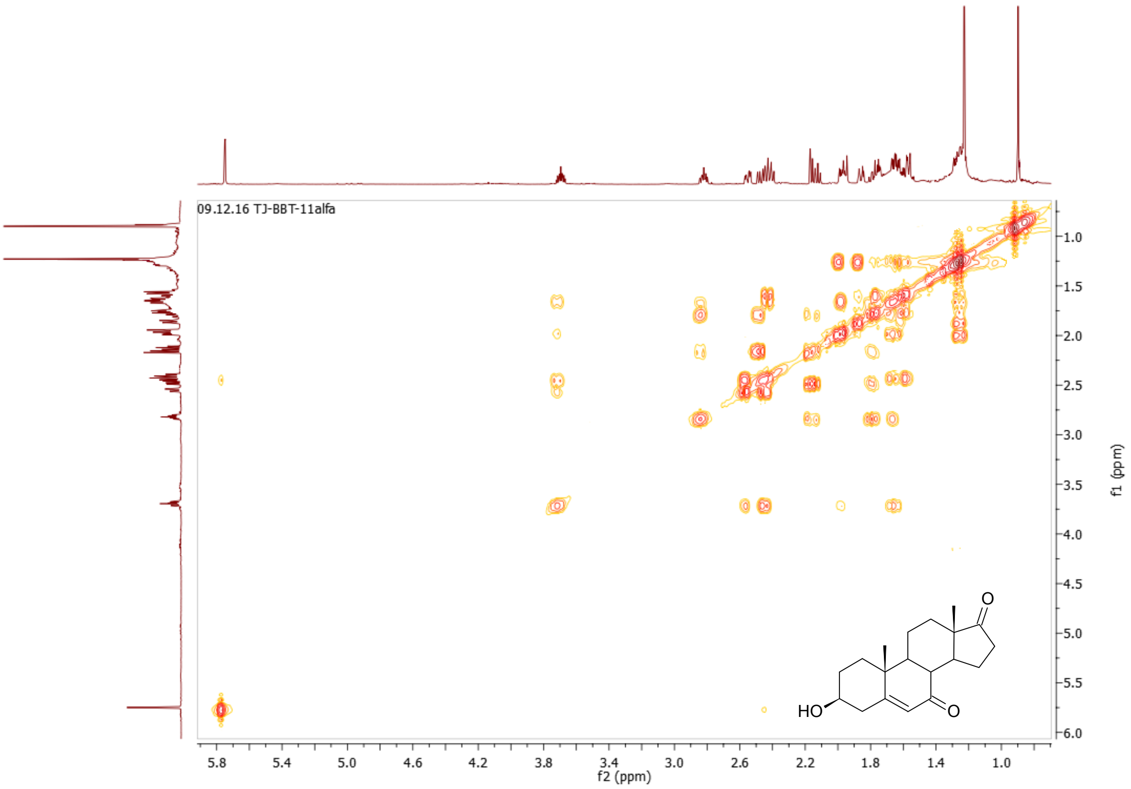

Fig.S33. ^{1}H NMR spectral of 3β,7α-dihydroxy-17a-oxa-D-homo-androst-5-en-17-one (**7α-OH-DHEA-lactone**) ($CDCl_3$, 600 MHz)

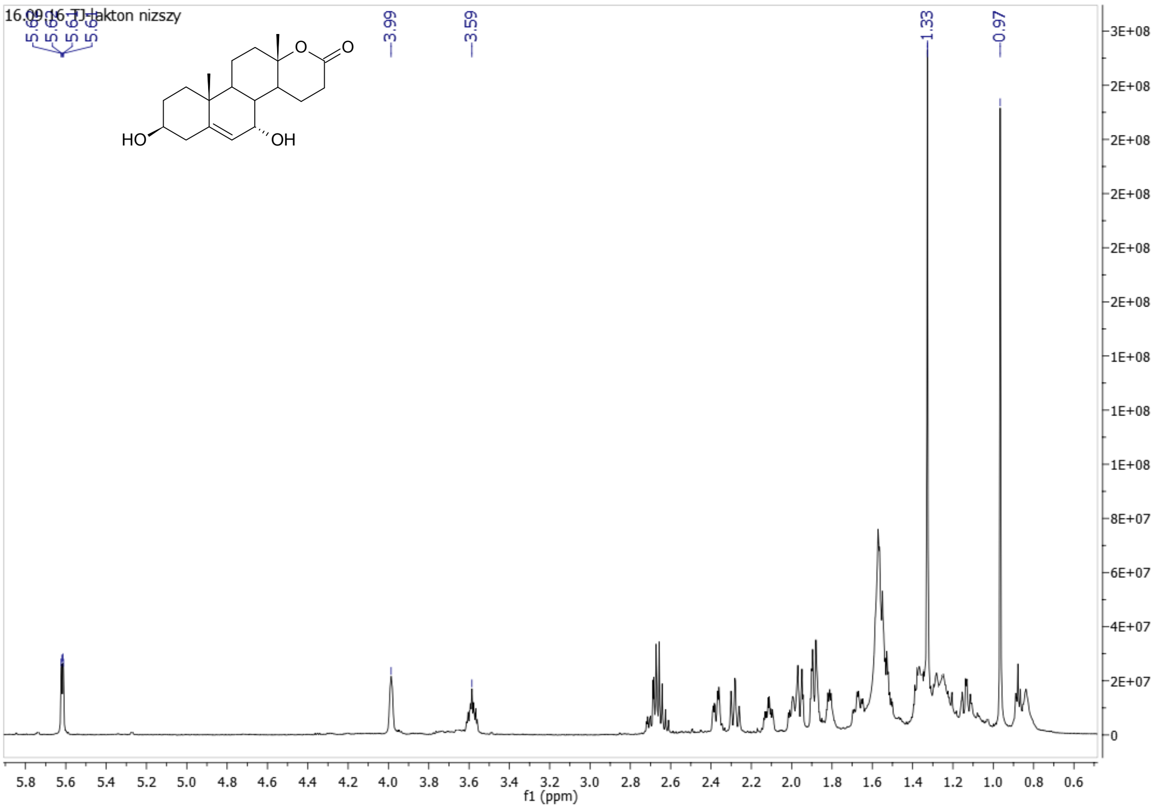

Fig.S34. ^{13}C NMR spectral of 3β,7α-dihydroxy-17a-oxa-D-homo-androst-5-en-17-one (**7α-OH-DHEA-lactone**) ($CDCl_3$, 151 MHz)

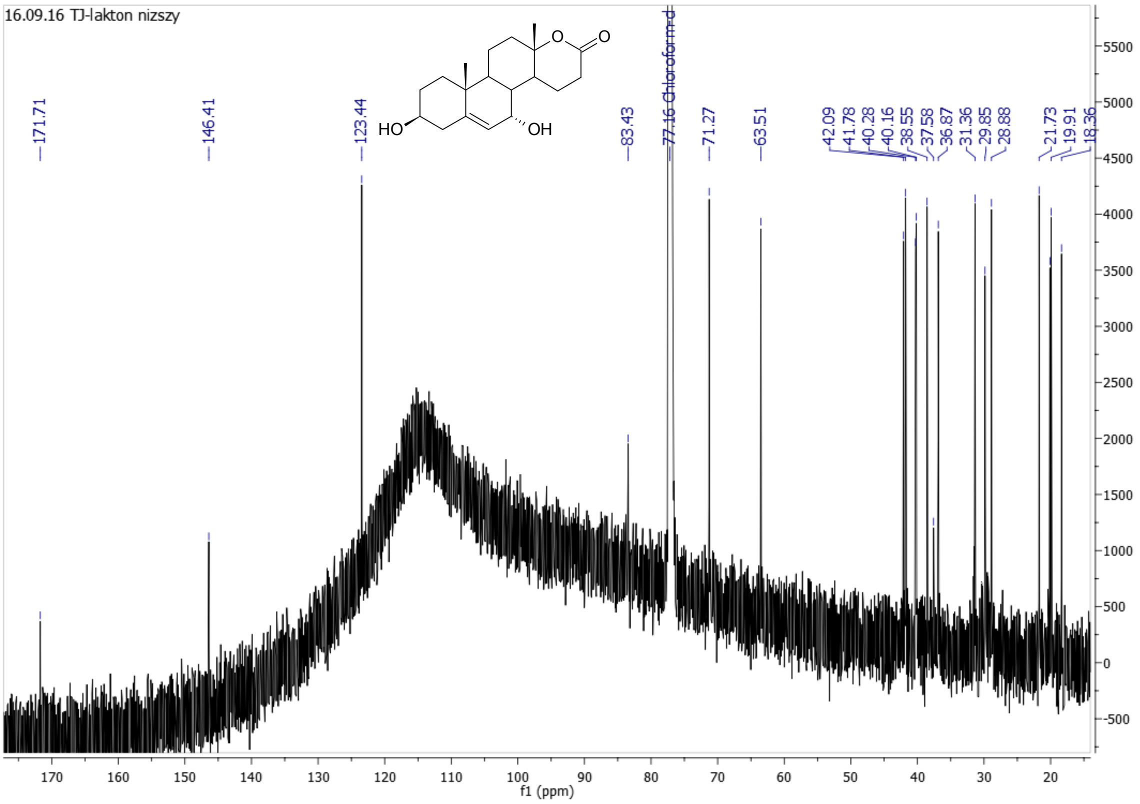

Fig.S35. HSQC spectral of 3β,7α-dihydroxy-17a-oxa-D-homo-androst-5-en-17-one (**7α-OH-DHEA-lactone**) ($CDCl_3$, 151 MHz)

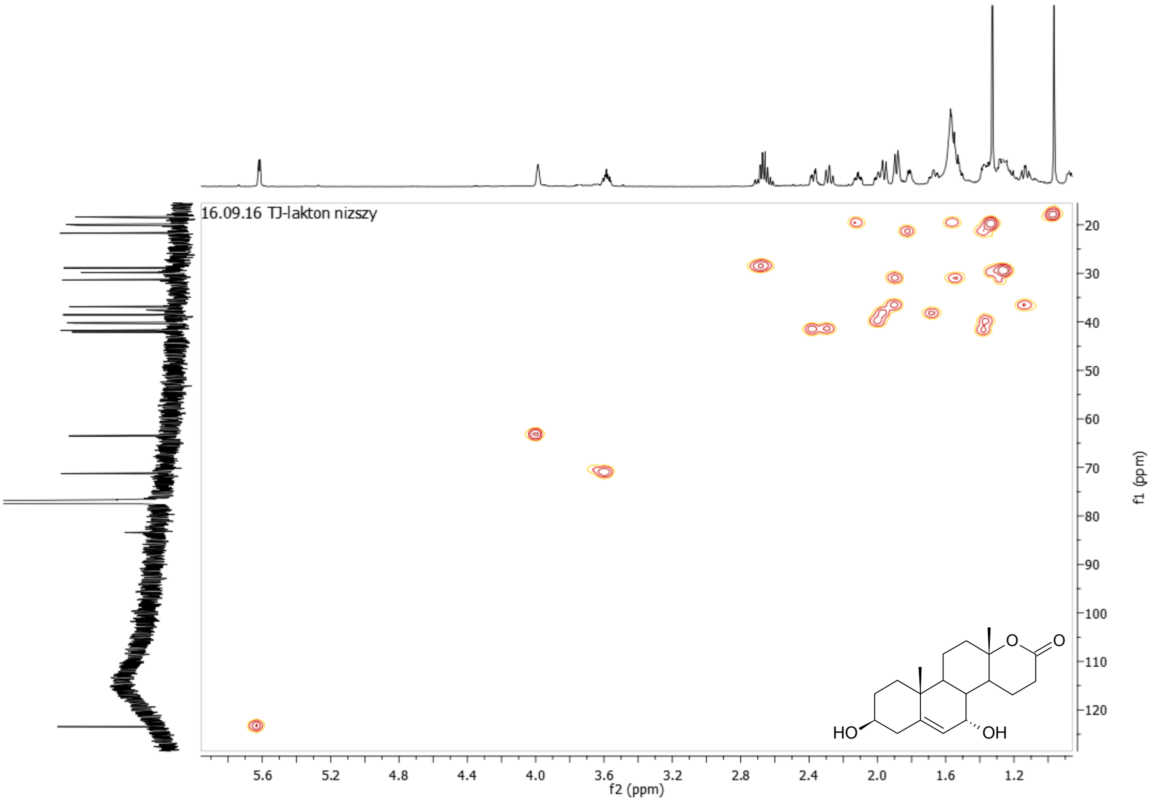

Fig.S36. ^{1}H NMR spectral of 3β,7β-dihydroxy-17a-oxa-D-homo-androst-5-en-17-one (**7βOH-DHEA-lactone**) ($CDCl_3$, 600 MHz)

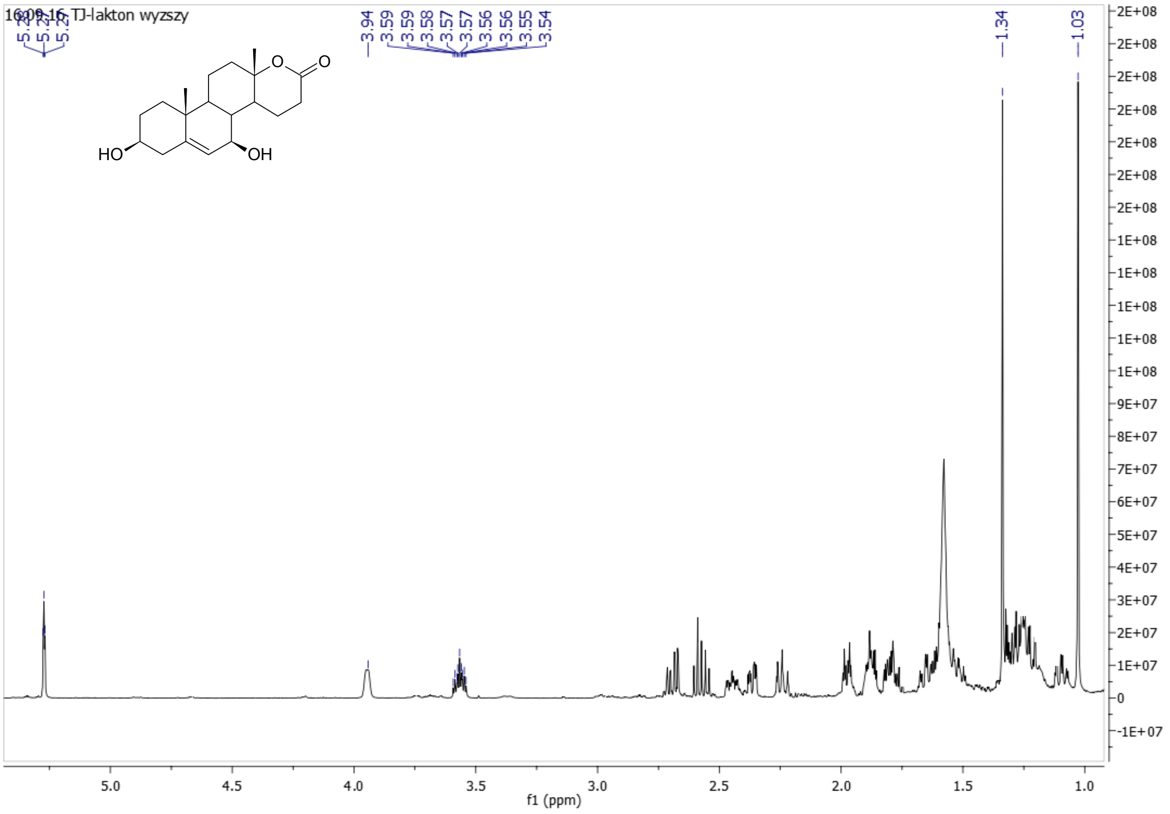

Fig.S37. ^{13}C NMR spectral of 3β,7β-dihydroxy-17a-oxa-D-homo-androst-5-en-17-one (**7βOH-DHEA-lactone**) ($CDCl_3$, 151 MHz)

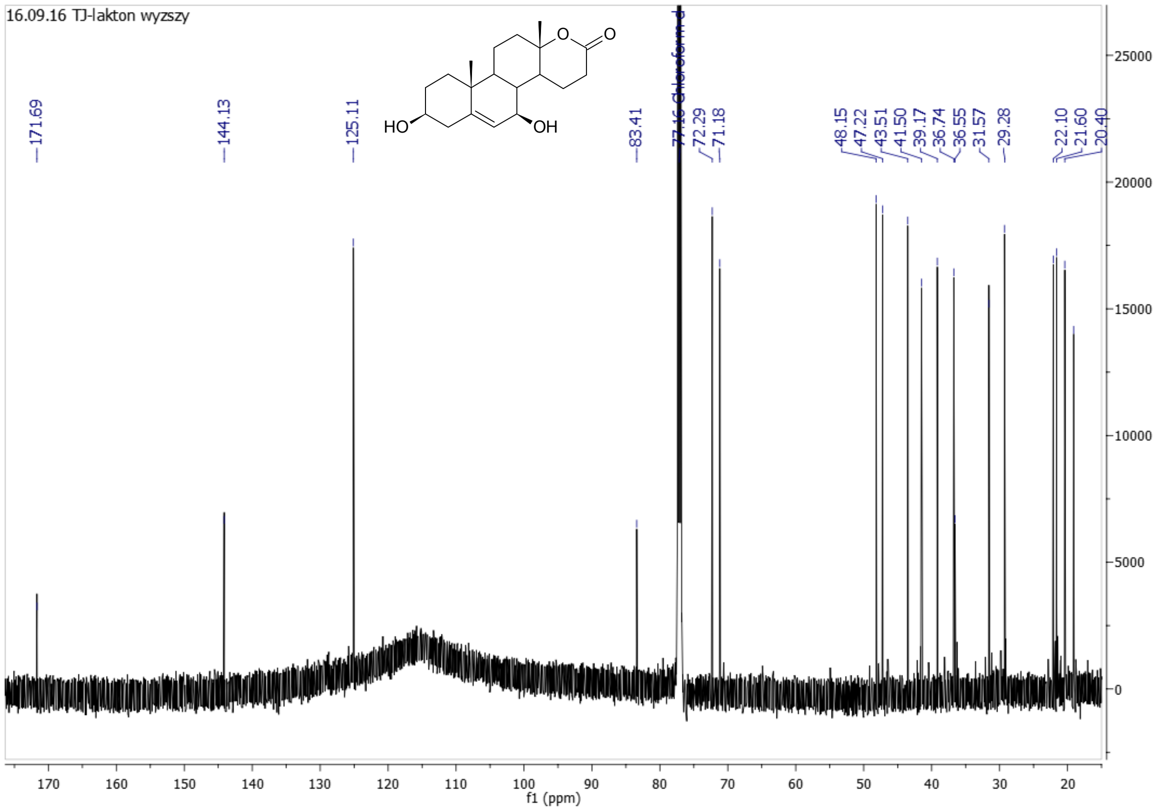

Fig.S38. HSQC spectral of 3β,7β-dihydroxy-17a-oxa-D-homo-androst-5-en-17-one (**7βOH-DHEA-lactone**) ($CDCl_3$, 151 MHz)

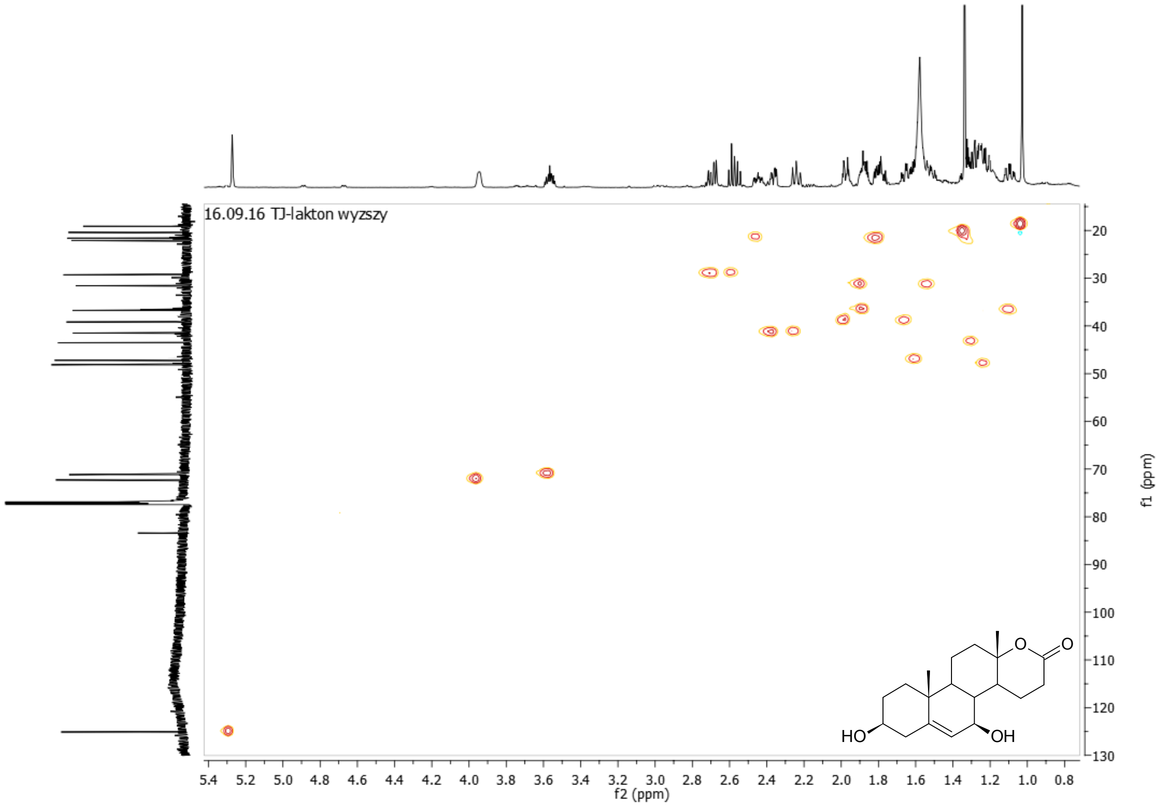

Fig.S39. GC-MS spectra of 7α-hydroxyandrost-4-ene-3,17-dione (**7α-OH-AD**)

Molecular Formula = $C_{19}H_{26}O_3$
Formula Weight = 302.40794

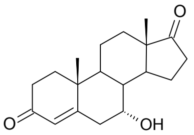

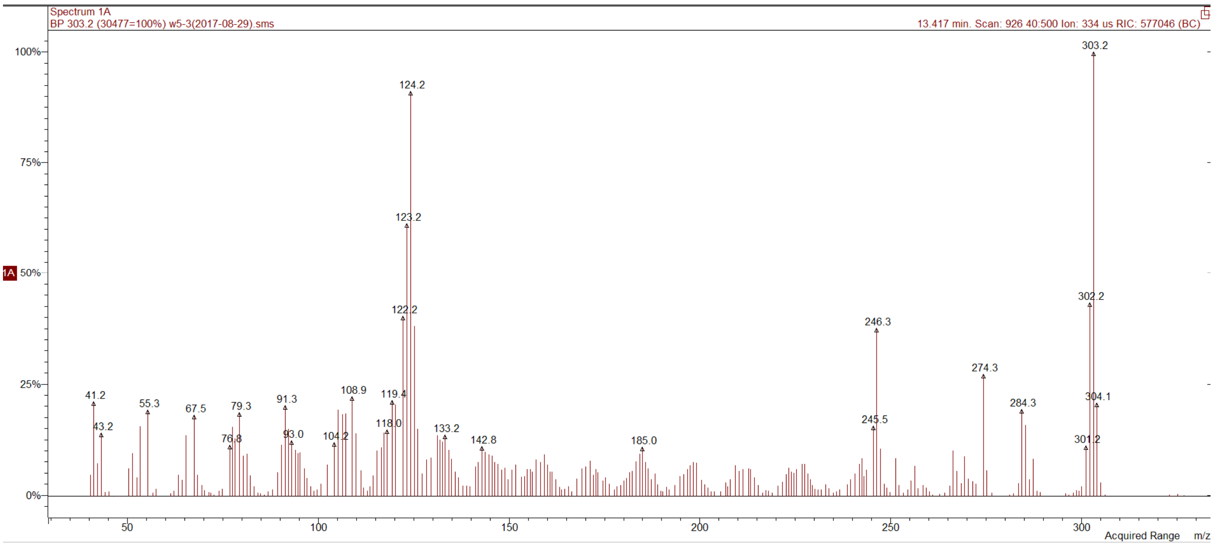

Fig.S40. Enlarged GC-MS spectra of 7α-hydroxyandrost-4-ene-3,17-dione (**7α-OH-AD**)

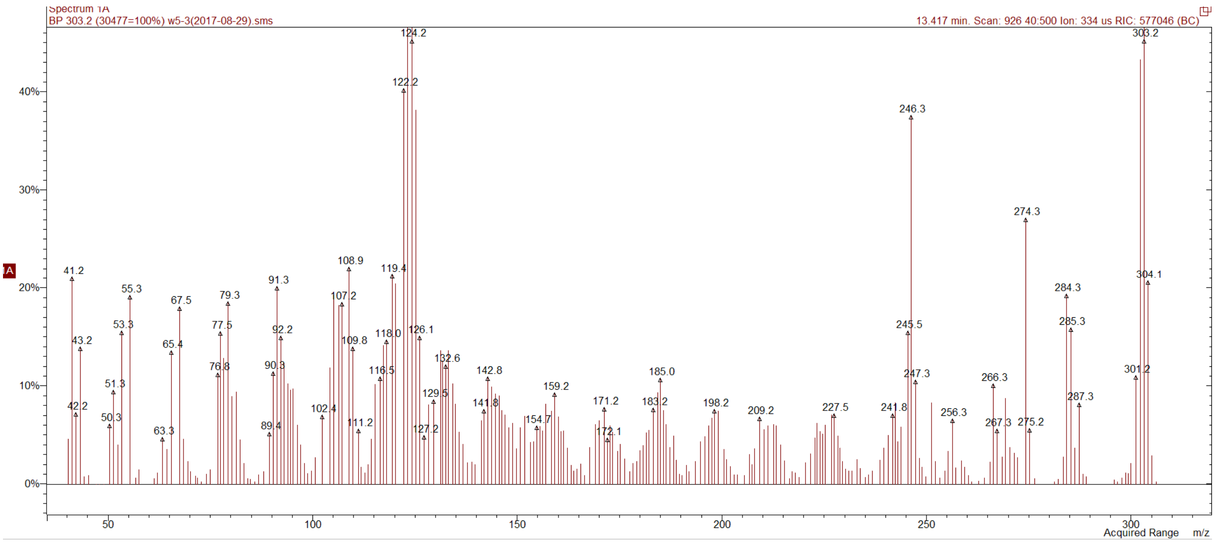

Fig.S41. GC-MS spectra of 6β-hydroxyandrost-4-ene-3,11,17-trione (**6β-OH-Adr**)

Molecular Formula $C_{19}H_{24}O_4$
Formula Weight = 316.39146

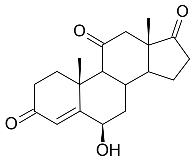

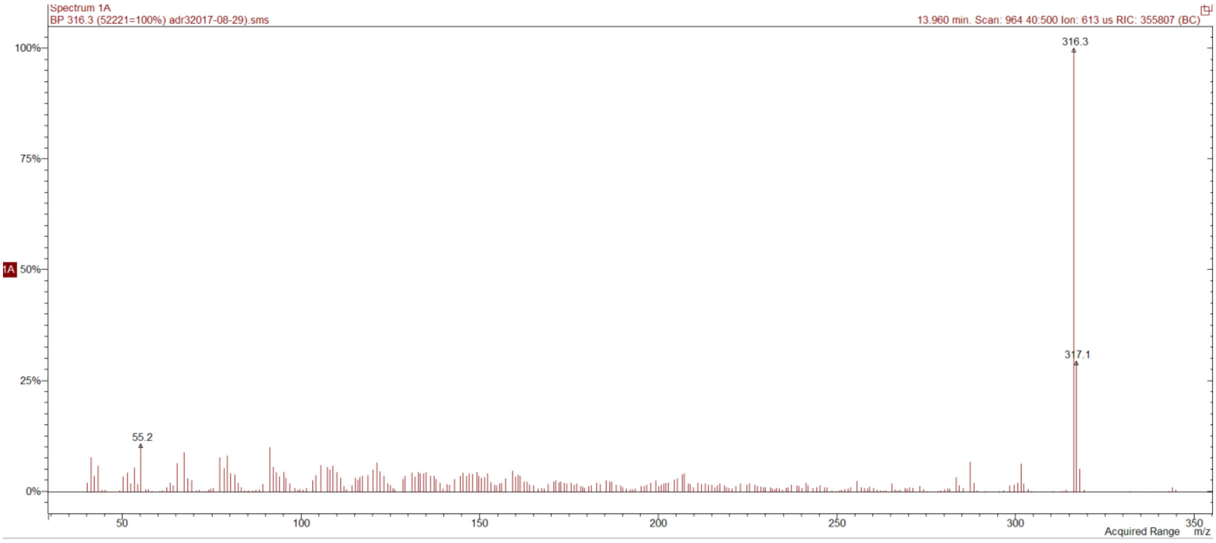

Fig.S42. Enlarged GC-MS spectra of 6β-hydroxyandrost-4-ene-3,11,17-trione (**6β-OH-Adr**)

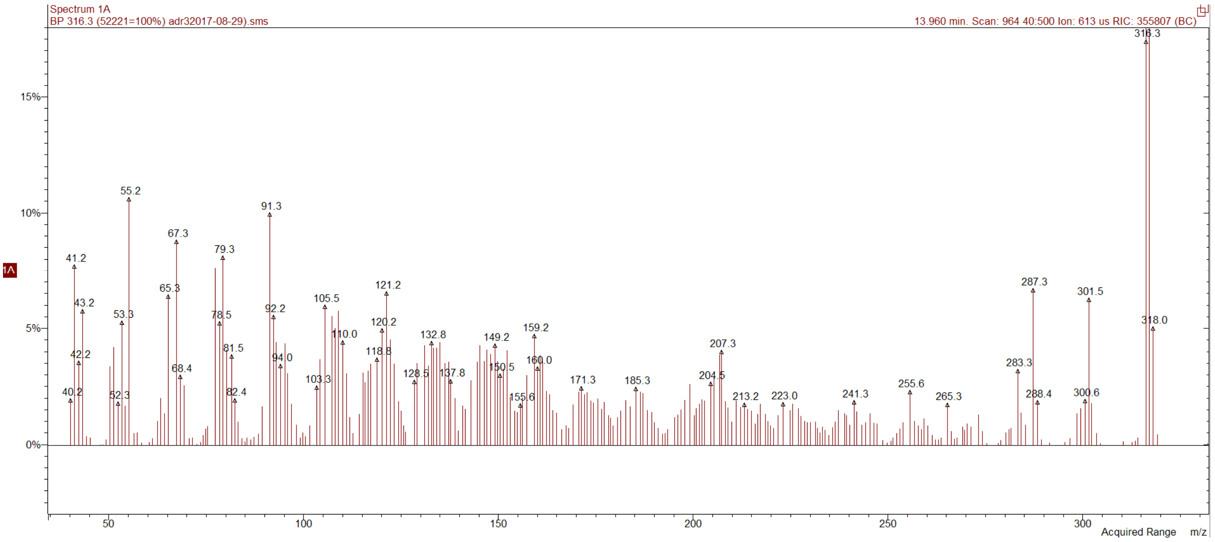

Fig.S43. GC-MS spectra of 15β-hydroxy-17α-methyltestosterone (**15β-OH-17mT**)

Molecular Formula = $C_{20}H_{30}O_3$
Formula Weight = 318.45040

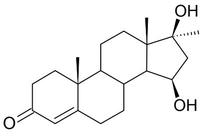

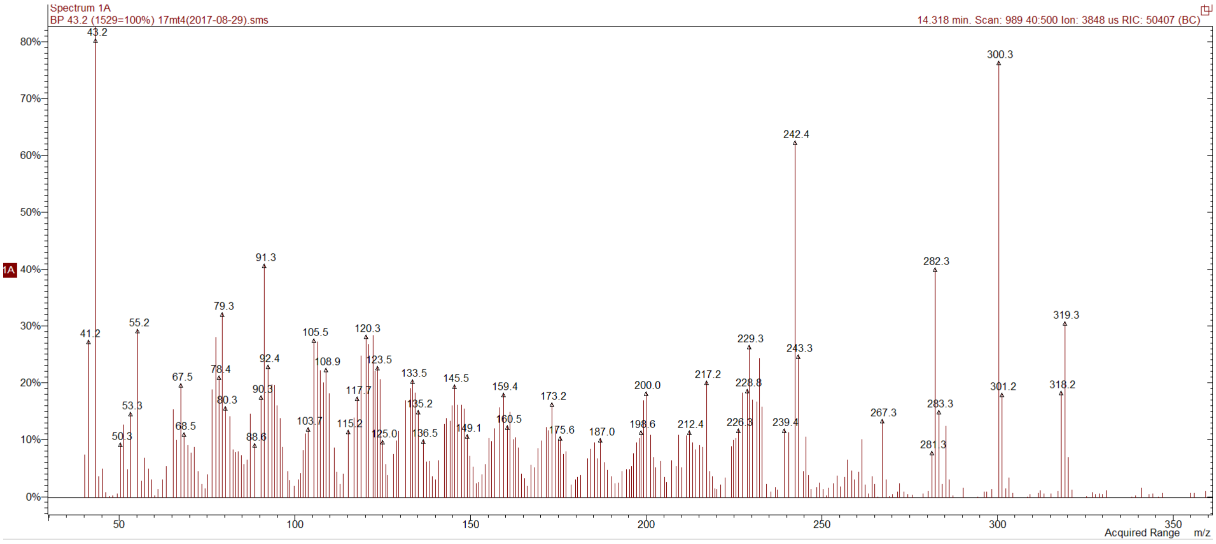

Fig.S44. GC-MS spectra of 6β-hydroxy-17α-methyltestosterone (**6β-OH-17mT**)

Molecular Formula = $C_{20}H_{30}O_3$
Formula Weight = 318.45040

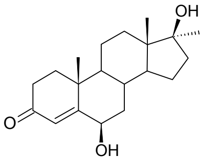

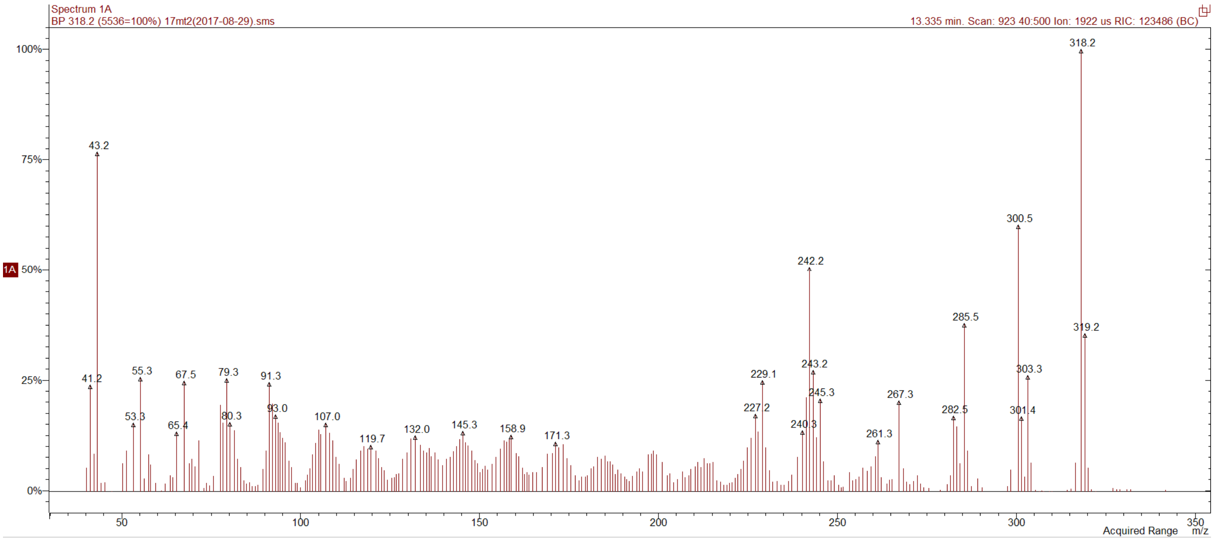

Fig.S45. GC-MS spectra of 6β,12β-dihydroxy-17α-methyltestosterone (**6β,12β-OH-17mT**)

Molecular Formula = $C_{20}H_{30}O_4$
Formula Weight = 334.44980

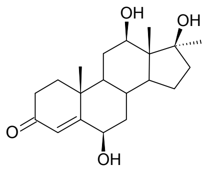

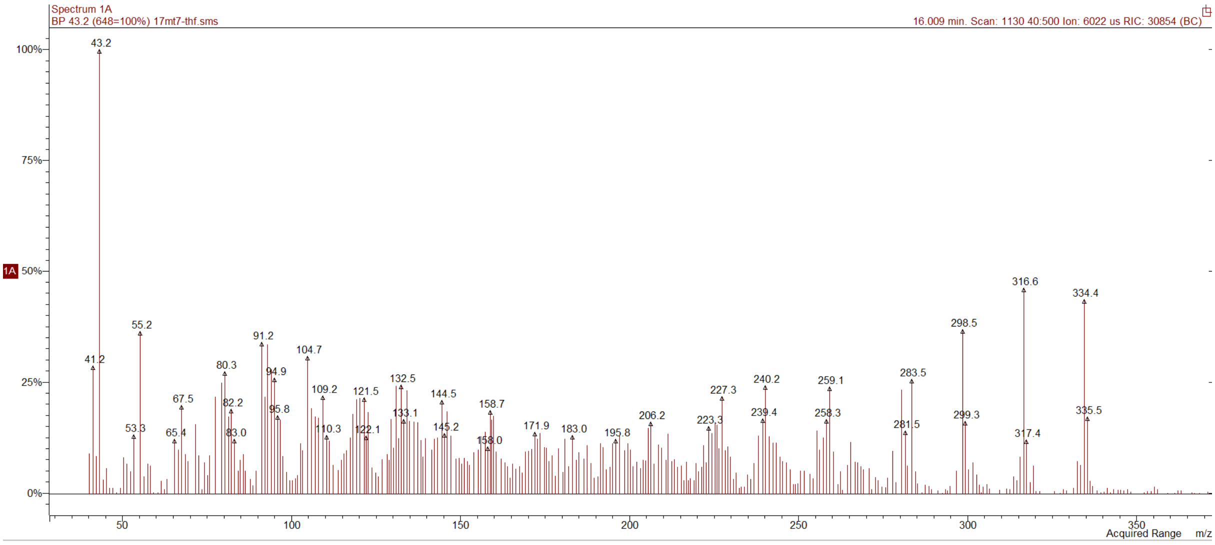

Fig.S46. Enlarged GC-MS spectra of 6β,12β-dihydroxy-17α-methyltestosterone (**6β,12β-OH-17mT**)

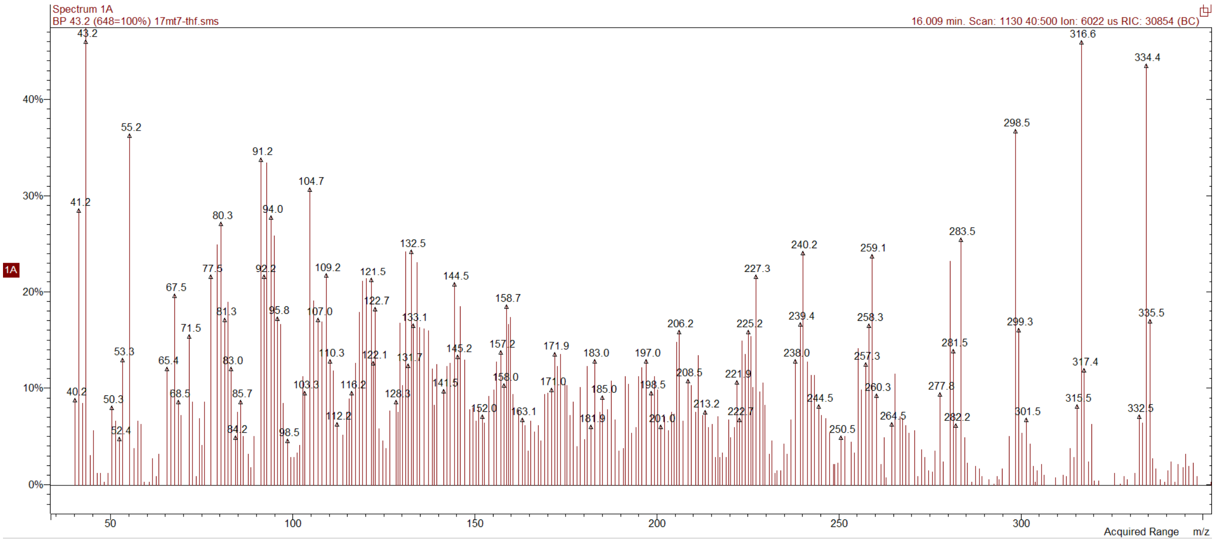

Fig.S47. GC-MS spectra of 3β,7α-dihydroxyandrost-5-ene-17-one (**7α-OH-DHEA**)

Molecular Formula = $C_{19}H_{28}O_3$
Formula Weight = 304.42382

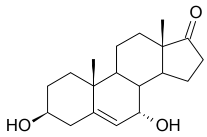

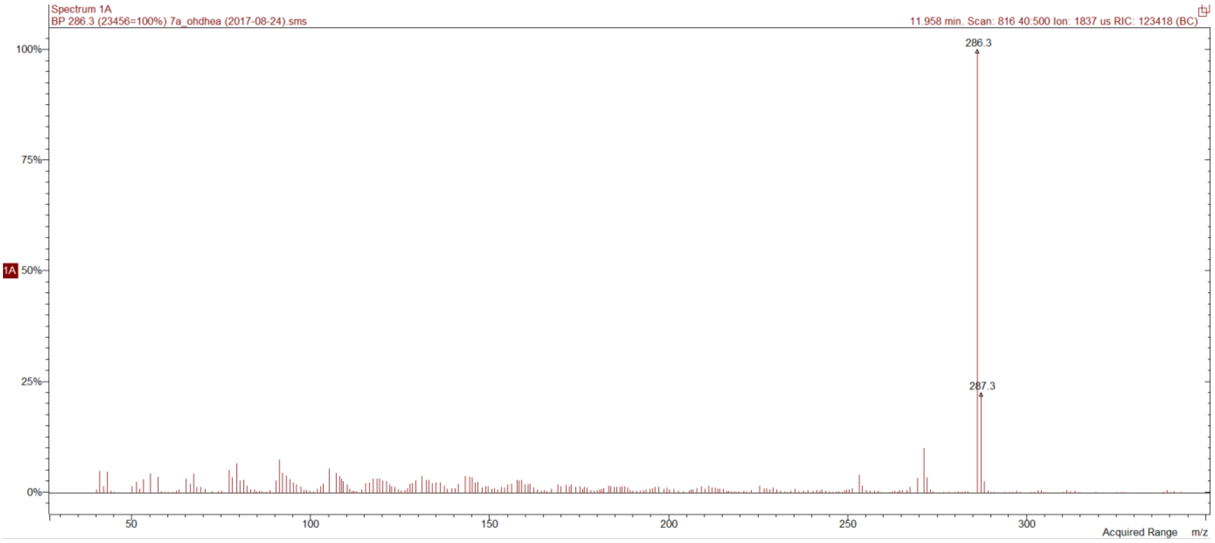

Fig.S48. Enlarged GC-MS spectra of 3β,7α-dihydroxyandrost-5-ene-17-one (**7α-OH-DHEA**)

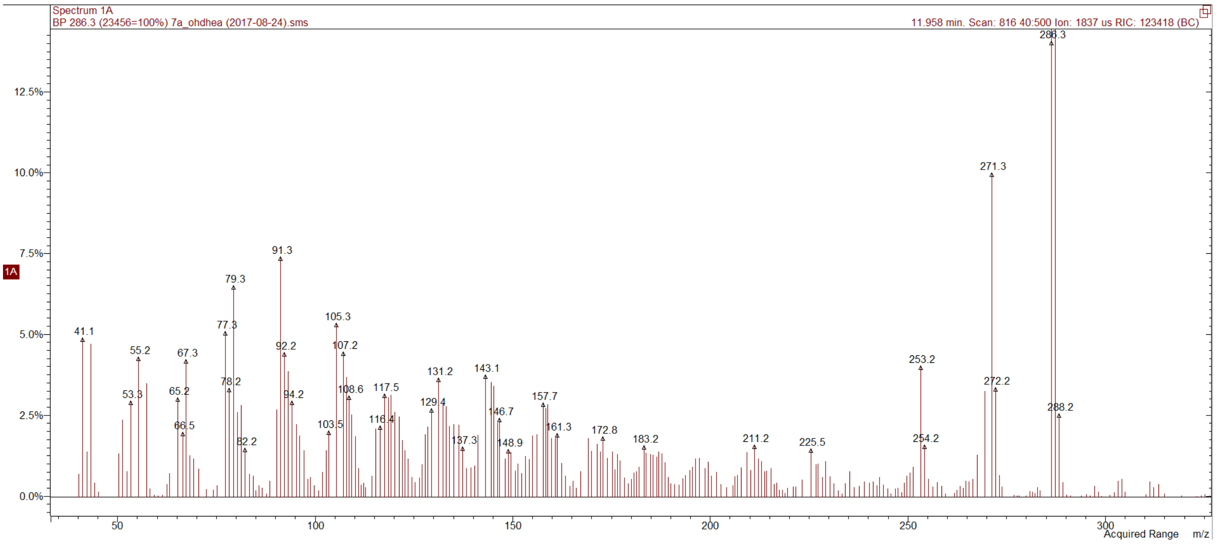

Fig.S49. GC-MS spectra of 3β,7β-dihydroxyandrost-5-ene-17-one (**7β-OH-DHEA**)

Molecular Formula = $C_{19}H_{28}O_3$
Formula Weight = 304.42382

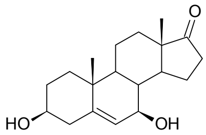

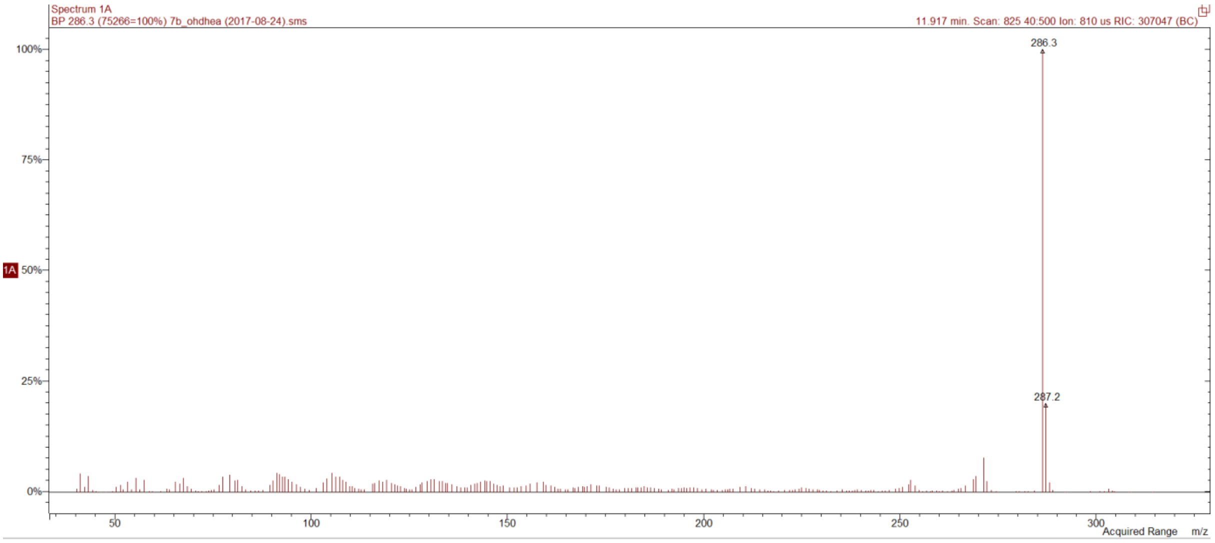

Fig.S50. Enlarged GC-MS spectra of 3β,7β-dihydroxyandrost-5-ene-17-one (**7β-OH-DHEA**)

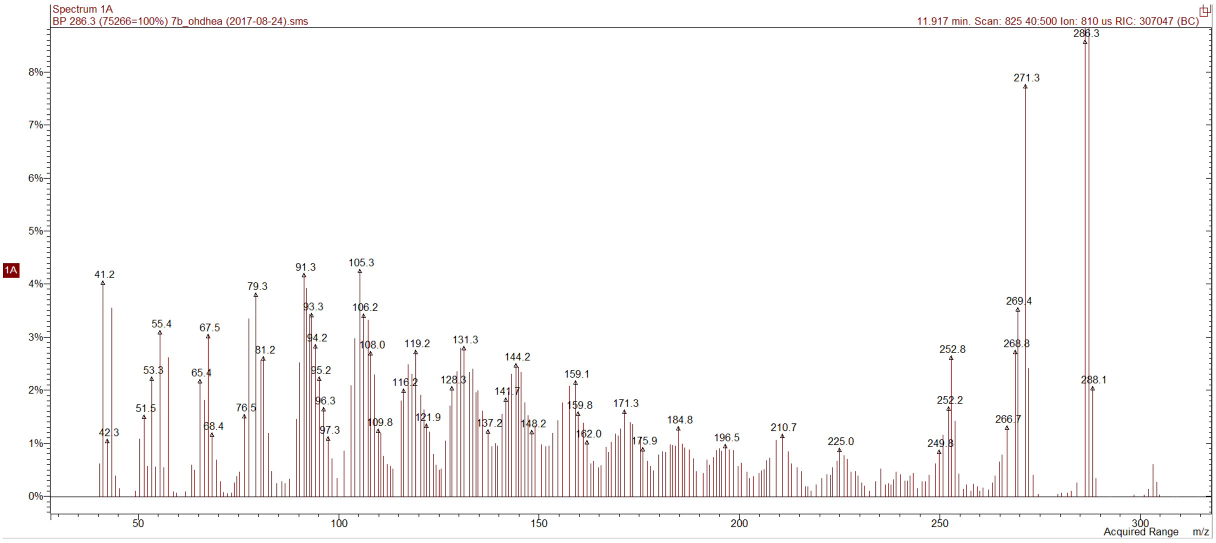

Fig.S51. GC-MS spectra of 3β-hydroxyandrost-5-ene-7,17-dione (**7-oxo-DHEA**)

Molecular Formula = $C_{19}H_{26}O_3$
Formula Weight = 302.40794

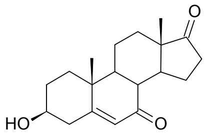

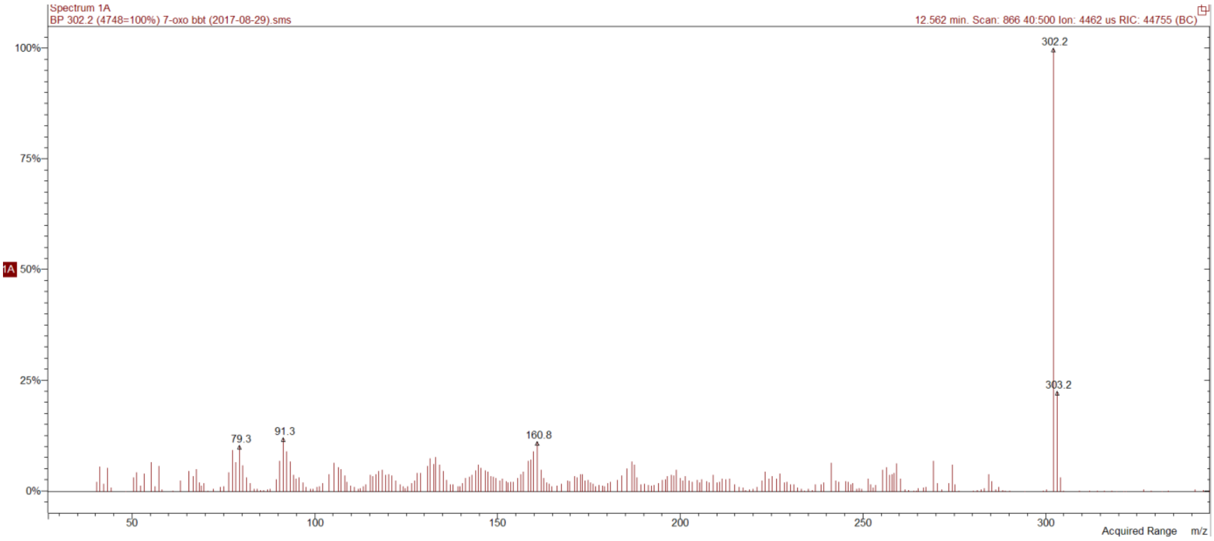

Fig.S52. Enlarged GC-MS spectra of 3β-hydroxyandrost-5-ene-7,17-dione (**7-oxo-DHEA**)

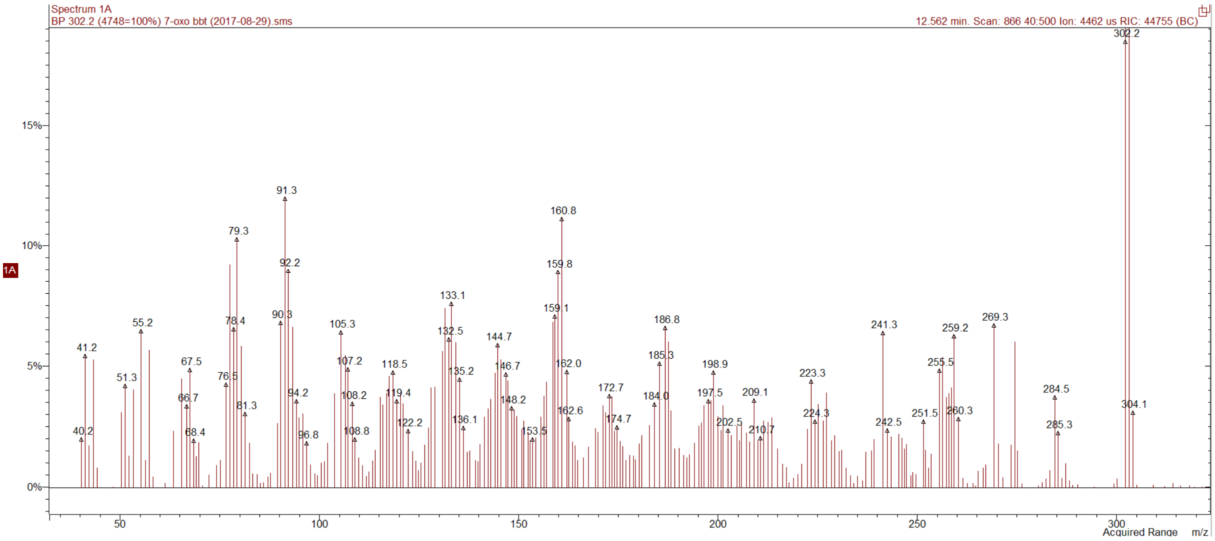

Fig.S53. GC-MS spectra of 3β,7α-dihydroxy-17a-oxa-D-homo-androst-5-en-17-one (**7α-OH-DHEA-lactone**)

Molecular Formula = $C_{19}H_{28}O_4$
Formula Weight = 320.42322

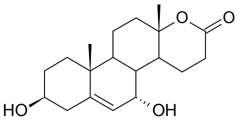

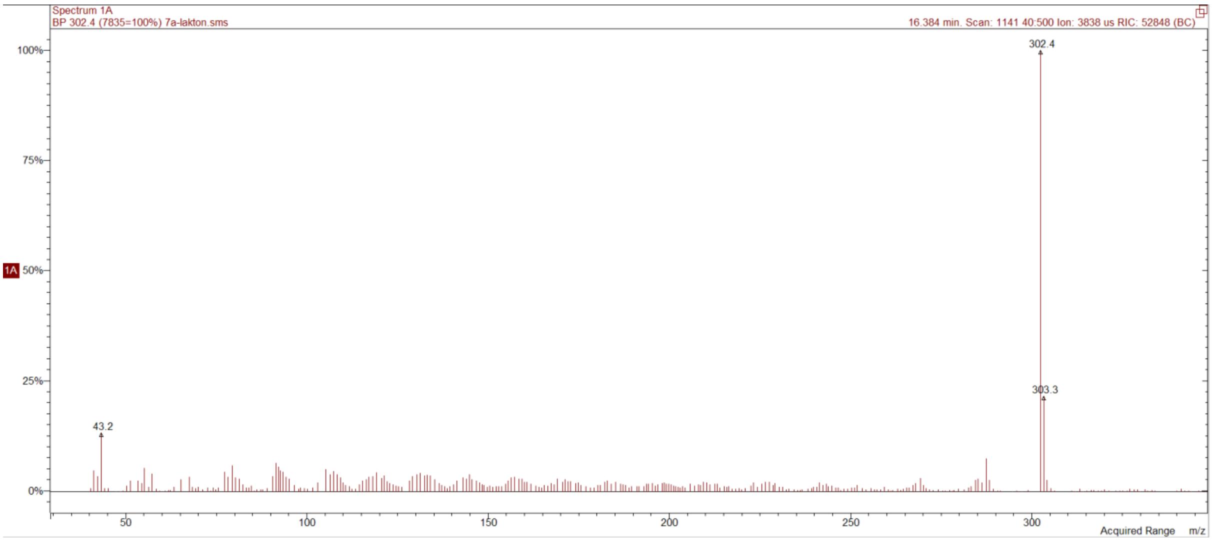

Fig.S54. Enlarged GC-MS spectra of 3β,7α-dihydroxy-17a-oxa-D-homo-androst-5-en-17-one (**7α-OH-DHEA-lactone**)

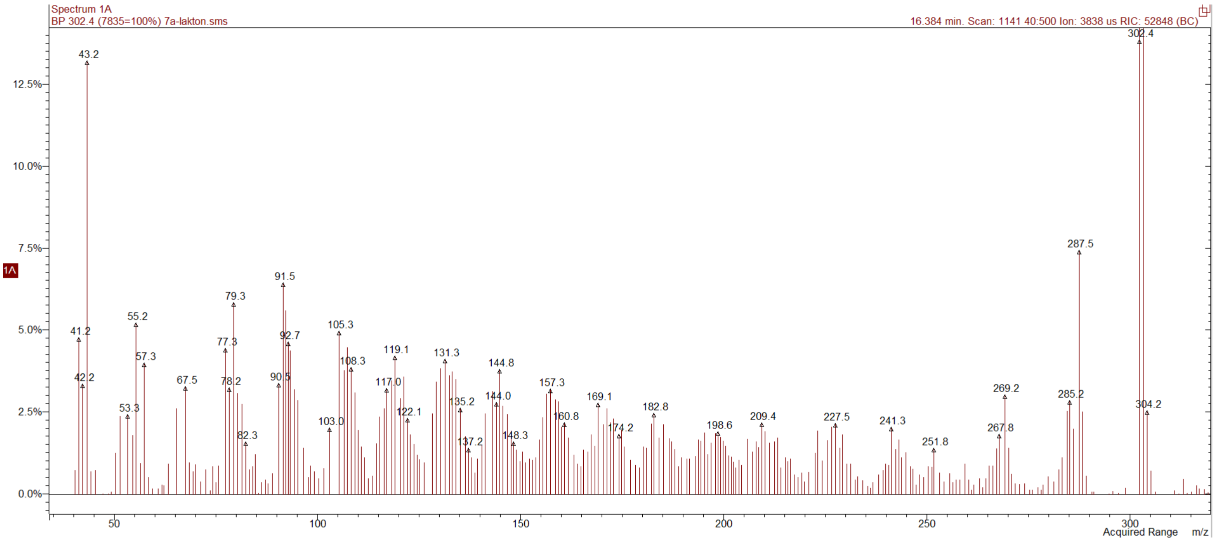

Fig.S55. GC-MS spectra of 3β,7β-dihydroxy-17a-oxa-D-homo-androst-5-en-17-one (**7β-OH-DHEA-lactone**)

Molecular Formula = $C_{19}H_{28}O_4$
Formula Weight = 320.42322

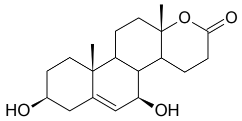

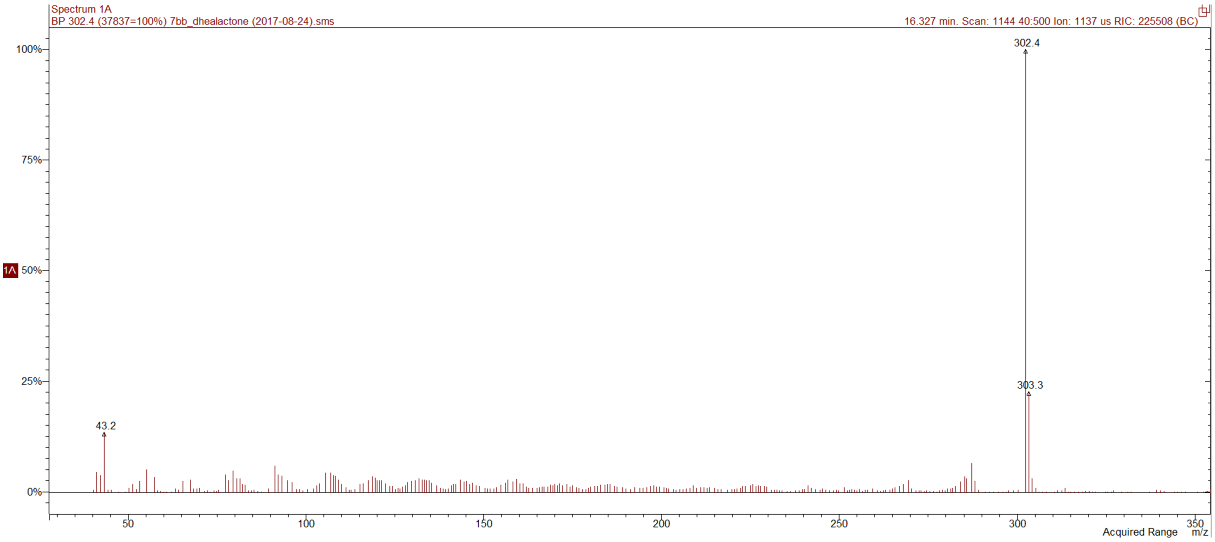

Fig.S56. Enlarged GC-MS spectra of 3β,7β-dihydroxy-17a-oxa-D-homo-androst-5-en-17-one (**7β-OH-DHEA-lactone**)

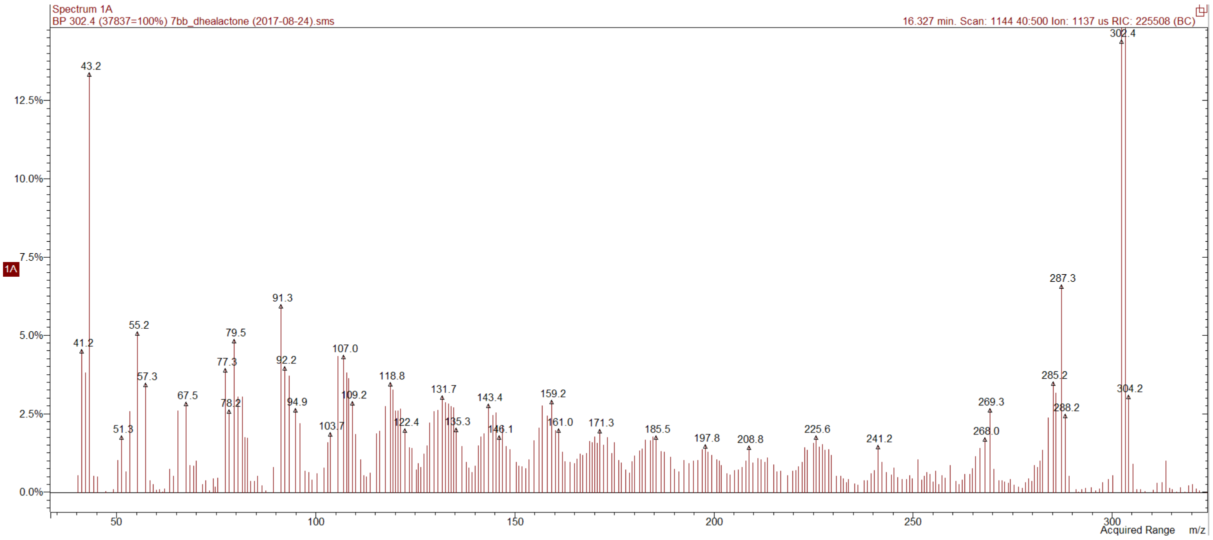

Supplement: Supplementary file 1 [file molecules-22-01511-s001.pdf]
